# Autosuppression of MdNAC18.1 endowed by a 61‐bp promoter fragment duplication delays maturity date in apple

**DOI:** 10.1111/pbi.14580

**Published:** 2025-02-26

**Authors:** Bo Zhang, Xiaofei Wang, Qianyu Yue, Weihan Zhang, Haofeng Liu, Tingting Zhang, Lingling Zhao, Qingmei Guan, Chunxiang You, Jianping An, Yuepeng Han, Liao Liao

**Affiliations:** ^1^ State Key Laboratory of Plant Diversity and Specialty Crops Wuhan Botanical Garden of Chinese Academy of Sciences Wuhan China; ^2^ Hubei Hongshan Laboratory Wuhan China; ^3^ University of Chinese Academy of Sciences Beijing China; ^4^ College of Horticulture Science and Engineering Shandong Agricultural University Tai‐An Shandong China; ^5^ State Key Laboratory for Crop Stress Resistance and High‐Efficiency Production/Shaanxi Key Laboratory of Apple, College of Horticulture Northwest A&F University Yangling Shaanxi China; ^6^ Yantai Academy of Agricultural Sciences Yantai China; ^7^ Sino‐African Joint Research Center Chinese Academy of Sciences Wuhan China

**Keywords:** Apple, Maturity date, Fruit ripening, Autoregulation, NAC transcription factor

## Abstract

Maturity date considerably influences fruit marketing period and commercial value and it is of particular importance in apple due to its association with fruit firmness that determines storage and shelf life, but the underlying mechanism remains unclear. In this study, we report a 61‐bp fragment duplication in the *MdNAC18.1* promoter that underpins maturity date variation in apple. *MdNAC18.1* is the crucial major gene for maturity date and was found to regulate fruit ripening by activating transcription of ethylene biosynthetic genes and ripening‐related transcription factors, including the MdNAC18.1 homologue MdNAC72 and the main regulator of JA signalling MdMYC2. Interestingly, MdNAC18.1 was capable of binding to the promoter itself containing an additional NAC recognition site that arose from the 61‐bp duplication to repress its own expression, but could not bind to its own promoter without the 61‐bp duplication. Thus, the *MdNAC18.1* allele with autosuppression function produces a phenotype of delayed maturity date and slower softening of fruit compared to that without autoregulation function. Our results demonstrate an autosuppression module that regulates the overall tempo of fruit ripening through fine‐tuning ethylene biosynthesis.

## Introduction

Fruit maturity date (MD) is an agriculturally important trait because it plays an important role in determining the marketing period and commercial value of the fruit. MD refers to the entire growth and development process of fruit, encompassing a series of intricate biological processes, such as fruit set, enlargement and ripening. MD is a complex quantitative trait that is influenced by internal genetic factors and external environmental factors. Genetic mapping of MD has been extensively investigated in fruit trees, with a frequent detection of large‐effect quantitative trait locus (QTL) (Baccichet *et al*., 2022; Migicovsky *et al*., 2016; Pirona *et al*., 2013). However, the genetic regulatory mechanisms of MD in fruit crops remain largely unclear.

Unlike MD, the regulatory mechanisms for fruit ripening have been well elucidated. During the ripening process, fleshy fruits undergo significant changes in colour, flavour, aroma, and texture (Seymour *et al*., 2013). Fruit ripening is attributed to the action of plant hormones such as ethylene, ABA and auxin (Liu *et al*., 2021; Ji *et al*., 2021; Yue *et al*., 2020). Fleshy fruits can be classified into climacteric and non‐climacteric types, with the former having a peak of ethylene production and respiration rate during ripening, while the latter lack the peak (Shinozaki *et al*., 2018). Abscisic acid (ABA) is the pivotal regulator in non‐climacteric fruits, and its interaction with ethylene is involved in the ripening of climacteric and non‐climacteric fruits (Chen *et al*., 2020; Qiao *et al*., 2021). Auxin regulates maturation by interacting with ethylene or ABA, respectively, in climacteric and non‐climacteric fruits (Yue *et al*., 2020; Busatto *et al*., 2021; Chen *et al*., 2023).

Transcription factors (TFs) that regulate fruit ripening through mediating ethylene biosynthesis have been well documented in tomato, including two master regulators, RIPENING INHIBITOR (RIN) and NON‐RIPENING (NOR). RIN belongs to the SEPALLATA clade MADS‐box genes which governs various aspects of fruit development and ripening. RIN is involved in the direct transcriptional regulation of ripening‐related genes (Fujisawa *et al*., 2013; Qin *et al*., 2012), and it also interacts with other ripening‐associated regulators such as Tomato AGAMOUS‐LIKE (TAGL1) and FRUITFULL 1/2 (FUL1/2) (Brumos, 2021; Li *et al*., 2020). The NAC TF NOR regulates fruit ripening through the ethylene and ABA signalling pathways (Kou *et al*., 2021). Homologues of NOR have been widely reported to regulate fruit ripening in a variety of fruit crops, such as strawberry (Li *et al*., 2023; Martin‐Pizarro *et al*., 2021), banana (Shan *et al*., 2020), and melon (Wang *et al*., 2022). In addition to their regulatory role in fruit ripening, two adjacent NAC TFs associated with MD, PpNAC1 and PpNAC5, have been shown to regulate fruit enlargement in peach (Zhang *et al*., 2024), consistent with the finding that ethylene participates in both vegetative and reproductive development in tomato (Huang *et al*., 2022; Sharma *et al*., 2021). Additionally, epigenetic regulation has been shown to play important roles in fruit ripening (Li *et al*., 2022; Lü *et al*., 2018; Zhong *et al*., 2013; Zhou *et al*., 2023).

The apple (*Malus × domestica* Borkh.) is a fleshy fruit recognized as a climacteric type of ripening. MD is a particular trait of interest in apple due to its positive correlation with fruit firmness that has a crucial role in determining storage and shelf life (Nybom *et al*., 2012). A number of quantitative trait loci (QTL) for MD in apple were identified based on linkage mapping studies in bi‐parental populations (Chagné *et al*., 2014). Whereas, genome‐wide association studies (GWAS) of unrelated individuals revealed a common large‐effect QTL on chromosome (Chr) 3, with a NAC TF designated MdNAC18.1 being a strong candidate gene for MD (Larsen *et al*., 2019; Migicovsky *et al*., 2016; Urrestarazu *et al*., 2017; Watts *et al*., 2023). The *MdNAC18.1* locus contains many DNA polymorphisms, but the causal variant(s) underlying MD remains unclear (Migicovsky *et al*., 2021).

The objective of this study was to identify causal variant(s) for MD in apple using genetic and chemical approaches. We uncovered negative autoregulation of *MdNAC18.1* transcription due to a duplication of 61‐bp fragment in the promoter, which causes a delay in maturity date in apple. Our findings provide novel insight into the molecular mechanisms related to the regulatory roles of NAC TFs in fruit development and ripening.

## Results

### Identification of candidate genes associated with fruit maturity date

**Figure 1:**
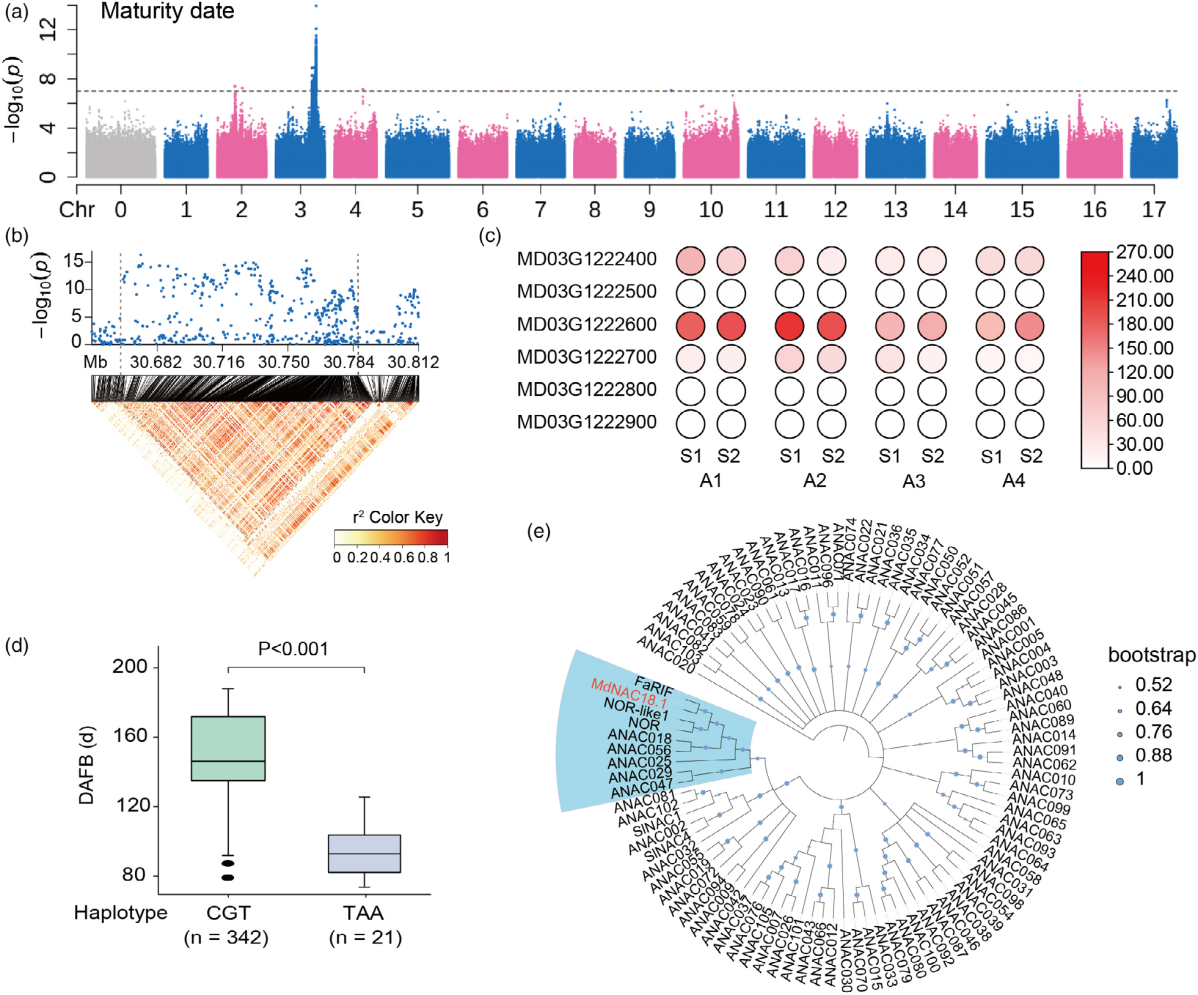
Identification of candidate genes for fruit maturity date in apple. (a) Manhattan plot for fruit maturity date. Dashed line represents the significance threshold at −log_10_(*P*) = 7.13. (b) Local Manhattan plot (top) and LD heatmap (bottom) surrounding the peak on Chr3. Vertical dashed grey lines indicate candidate region for the peak on Chr3. (c) Heat maps showing expression profiles of six genes within the candidate region at the expanding (S1) and ripening (S2) stages of different apple accessions. A1 and A2 represent early‐maturing varieties ‘Calville Rouge’ and ‘Weiqinni’, respectively, while A3 and A4 indicate late‐maturing accessions *M. coronaria* and *M. sikkimensis*. The intensity of the circle's hue corresponds to gene expression level. (d) Boxplots for fruit maturity date based on two types of haplotypes. In the box plots, box limits are the upper and lower quartiles with the median values shown by bold lines. Whiskers denote 1.5 × interquartile range and outliers are shown with solid black dots. *P* value was estimated based on Student's *t*‐test. (e) Phylogenetic tree analysis of NAC transcription factors in various species.

To identify candidate genes for MD, we conducted GWAS using our previously reported collection of 461 apple accessions (Liao *et al*., 2021). The collection samples showed a great variation in MD, with a range of 76 to 177 days after full bloom (DAFB) (Table S1). GWAS results showed a strong peak on Chr03 that covered 630 significantly associated SNPs (–log_10_ *P* > 7.13) (Figure 1a; Table S2). The most significant SNP (–log_10_ *P* = 14.71) is located in the intergenic region between *MD03G1222700* and the previously reported *MdNAC18.1* (*MD03G1222600*). Linkage disequilibrium (LD) analysis revealed a candidate region of ~120 kb spanning from 30.66 to 30.78 Mb, which encompassed the top 10 most significant MD‐associated SNPs and six annotated genes (Figure 1b). Expression profiling of the six genes was investigated in the fruits of two early‐maturing and two late‐maturing apple accessions at expanding (S1) and ripening (S2) stages. Three genes, *MD03G1222500*, *MD03G1222800*, and *MD03G1222900*, showed extremely low expression throughout fruit development (FPKM < 0.1), and two genes, *MD03G1222400* and *MD03G1222700*, exhibited consistent expression patterns across all tested accessions (Figure 1c). By contrast, *MdNAC18.1* showed higher levels of expression in the fruits of early‐maturing varieties during the expanding and ripening stages than in the fruits of late‐maturing varieties. The genomic region of *MdNAC18.1* contained abundant sequence variations (Table S2), with two nonsynonymous and one synonymous SNPs in the third exon exhibiting the strongest signals (–log_10_ *P* = 10.54). The three SNPs on the third exon formed two haplotypes, CGT and TAA, which showed a significant difference in MD (Figure 1d). *MdNAC18.1* was phylogenetically related to well‐known ripening‐related genes such as strawberry *FaRIF* and tomato *NOR* (Figure 1e). These results suggested that *MdNAC18.1* is a strong candidate gene for MD in apple.

### *MdNAC18.1* acts as a regulator of fruit ripening

**Figure 2:**
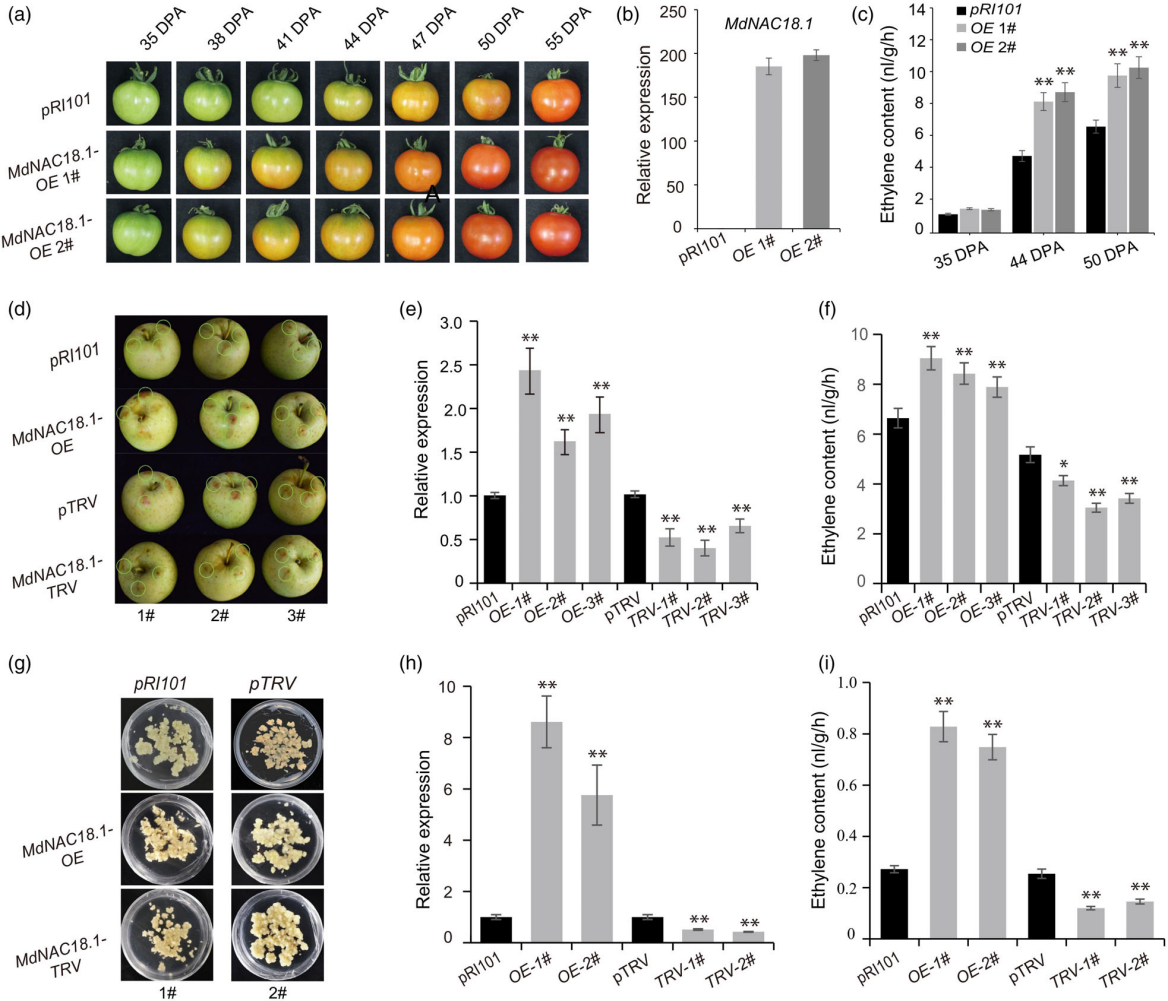
Functional analysis of *MdNAC18.1* through stable or transient transformation assays. (a) Tomato fruits of transgenic lines carrying *MdNAC18.1* or empty vector (*pRI101*) at different stages of development. DPA, day post anthesis. (b) Relative expression of *MdNAC18.1* in transgenic tomato fruits. (c) Ethylene content in transgenic tomato fruits at different stages of development. (d) Transient overexpressing and silencing *MdNAC18.1* in apple fruits of ‘gala’ at 100 DAFB. The infiltration sites are indicted with white circles. (e) Relative expression of *MdNAC18.1* in apple fruits 7 days after infiltration. (f) Ethylene content in apple fruits 7 days after infiltration. (g) Overexpressing and silencing *MdNAC18.1* in apple calli of ‘Orin’. (h) Relative expression of *MdNAC18.1* in transgenic apple calli. (i) Ethylene content in transgenic apple calli. Empty vectors, *pRI101* and *pTRV*, were used as control. Error bar means the SD values, and asterisks represent significant differences based on Student's *t*‐test. *P < 0.05, **P < 0.01.

To elucidate the role of *MdNAC18.1* in fruit ripening, its full coding sequence was introduced into tomato variety ‘Alisa Craig (AC)’ under the control of the 35S promoter. Two transgenic lines showing high levels of *MdNAC18.1* expression were selected to investigate the ripening behaviour (Figure 2b). **Transgenic** fruits showed colour break at 38 days post anthesis (DPA), with red‐coloration at 50 DPA (Figure 2a). Whereas, the fruits transformed with empty‐vector (EV) had colour break at 44 DPA, with orange coloration at 55 DPA. Ethylene contents in fruits overexpressing *MdNAC18.1* and EV were comparable at 35 DPA. However, a significant increase in ethylene content was observed in *MdNAC18.1‐*overexpressing fruits at 44 and 50 DPA compared to the EV control (Figure 2c). Overall, transgenic lines showed accelerated ripening compared to the EV control, suggesting a positive regulatory role of *MdNAC18.1* in fruit ripening.

The positive regulatory role of *MdNAC18.1* in fruit ripening was validated using transient transformation assay in apple fruits and stable transformation assay in apple calli. Ethylene content in flesh tissues overexpressing *MdNAC18.1* was significantly higher compared to the EV infiltration, whereas an opposite result was observed for *MdNAC18.1‐*silenced fruits (Figure 2d–f). Similarly, apple calli overexpressing *MdNAC18.1* exhibited significantly higher levels of ethylene accumulation compared to the EV control, while ethylene accumulation reduced significantly in *MdNAC18.1‐*silenced apple calli (Figure 2g–i). These results suggested that MdNAC18.1 promotes fruit ripening through activating ethylene biosynthesis. Notably, the expression levels of two known ethylene biosynthetic genes, *MdACO1* and *MdACS1*, were examined. The expression level of *MdACS1* was significantly upregulated and downregulated, respectively, in *MdNAC18.1‐*overexpressing and *MdNAC18.1‐*silenced apple fruits or calli, whereas, there were no significant changes in the *MdACO1* expression (Figure S1).

### MdNAC18.1 activates transcription of ethylene biosynthetic genes

**Figure 3:**
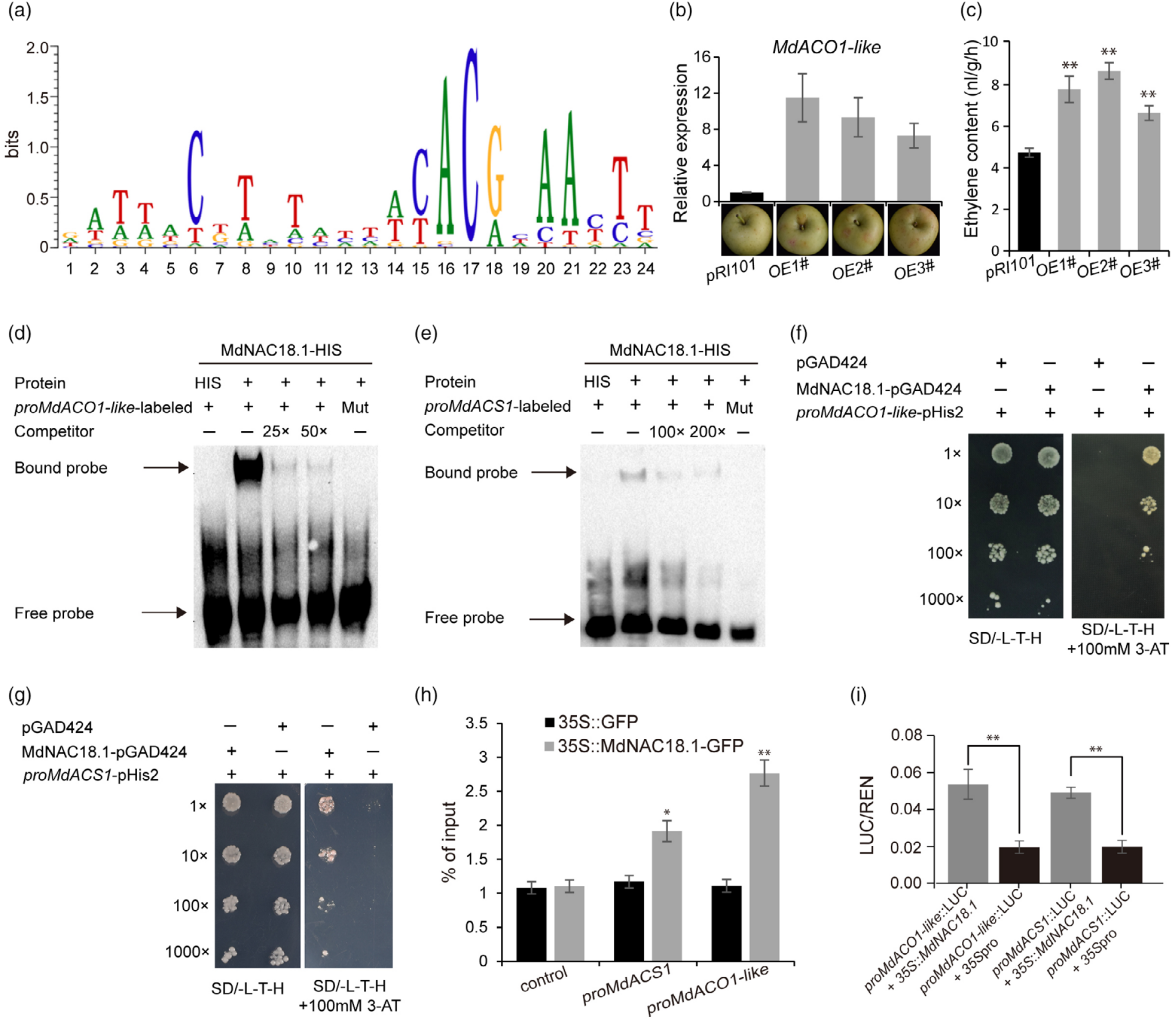
The MdNAC18.1 protein interacts with the promoter region of *MdACO1‐like* and *MdACS1* and enhances its transcriptional activity. (a) Identification of MdNAC18.1 recognition sites using the DAP‐seq method. The sequence motif was calculated using Multiple EM for Motif Elicitation (MEME), with a statistical significance (E‐value) of 5.9e‐168. (b) Transient overexpression of *MdACO1‐like* in apple fruits of ‘Gala’. (c) Ethylene content in flesh tissues of apple fruits seven days after infiltration with empty vector (pRI101) or *MdACO1‐like*. (d) EMSA assays showed that MdNAC18.1 directly binds to the promoter of *MdACO1‐like* in vitro. The MdNAC18.1‐binding motif is ‘TACACG’, while the mutated version is ‘AAAAAA’. (e) EMSA assays indicated that MdNAC18.1 directly binds to the promoter of *MdACS1* in vitro. The MdNAC18.1‐binding motif is ‘CCCCACG’, while the mutated version is ‘AAAAAAA’. (f‐g) Analysis of the interaction between MdNAC18.1 and the *MdACO1‐like* or *MdACS1* promoter using Y1H. (h) ChIP‐PCR assays showing that MdNAC18.1‐GFP facilitates the enrichment of promoter fragments of *MdACS1* and *MdACO1‐like* genes. Unbound probe serves as the control. (i) Assay of activation effect of MdNAC18.1 on the *MdACO1‐like* and *MdACS1* promoter using dual‐luciferase assay. Error bar means the SD values of three biological replicates. Asterisks represent significant differences based on Student's *t*‐test. *P < 0.05, **P < 0.01.

To get insight into the regulatory role of *MdNAC18.1* in fruit ripening, its target genes were investigated using DNA affinity purification sequencing (DAP‐seq) method. Approximately 27 million reads from two biological replicates were generated using the Illumina PE150 platform. Over 81% of the reads were uniquely mapped to the apple reference genome GDDH13 v.1.1. Multiple EM for Motif Elicitation (MEME) was used to identify MdNAC18.1 binding sites and a potential recognition motif with the consensus sequence CACG was identified (Figure 3a).

Sequencing reads containing the core sequence CACG were enriched in 2317 regions that contain 558 annotated genes (Table S3). The Gene Ontology (GO) and Kyoto Encyclopedia of Genes and Genomes (KEGG) enrichment analysis revealed these genes were associated with cell wall biogenesis, cellulose biosynthetic process and cellulose synthase activity (Figure S2). Among these enriched genes, we found two candidate genes related to ethylene synthesis: *MdACS1* and *MdACO1‐like*. The former is a well‐known gene involved in ethylene biosynthesis (Oraguzie *et al*., 2004), while the role of the latter in ethylene biosynthesis remains uncertain. Transient overexpression of *MdACO1‐like* in apple fruits resulted in accelerated red‐coloration compared to the EV control (Figure 3b), accompanied by a significant induction of ethylene biosynthesis (Figure 3c). Similarly, apple calli overexpressing *MdACO1‐like* exhibited higher levels of ethylene accumulation compared to the EV control (Figure S3). These results indicated that *MdACO1‐like* was indeed an ethylene biosynthetic gene.

The *MdACO1‐like* promoter contained three potential NAC binding sites (NACBS) with the core sequence CACG that are known as NACBS1/2 (Figure S4). We tested whether MdNAC18.1 could bind to NACBS1/2 in the *MdACO1‐like* promoter using EMSA with a purified recombinant MdNAC18.1 protein and labelled DNA probes containing the core sequence CACG. The results showed that the MdNAC18.1‐HIS fusion protein could bind to the NACBS1 recognition site, but the binding capacity was disrupted when the NACBS1 motif was mutated (Figure 3d). Unexpectedly, the MdNAC18.1‐HIS fusion protein was unable to bind the NACBS2 recognition site. In addition, five potential NAC binding sites with the core sequence CACG that correspond to NACBS3/4/5 were identified in the *MdACS1* promoter (Figure S4). EMSA assay indicated that the MdNAC18.1‐HIS fusion protein could bind to the NACBS5 recognition site, and this binding ability was lost when the NACBS5 motif was mutated (Figure 3e). However, the MdNAC18.1‐HIS fusion protein was unable to bind NACBS3 and NACBS4.

The binding affinity of MdNAC18.1 to the *MdACO1‐like* or *MdACS1* promoter was confirmed using yeast one‐hybrid (Y1H) assay (Figure 3f,g). Chromatin immunoprecipitation qPCR (ChIP‐qPCR) assays demonstrated a significant enrichment of the promoter fragments of the *MdACS1* and *MdACO1‐like* genes by immuneprecipitation. This finding indicated that MdNAC18.1 could bind to the promoters of *MdACS1* and *MdACO1‐like* in vivo (Figure 3h). Moreover, the dual luciferase assay showed that MdNAC18.1 was able to induce the *MdACO1‐like* or *MdACS1* promoter (Figure 3i). Taken together, these results indicated that MdNAC18.1 could activate transcription of ethylene biosynthetic genes through directly binding to their promoters.

### MdNAC18.1 regulates transcription of fruit ripening‐related TFs

**Figure 4:**
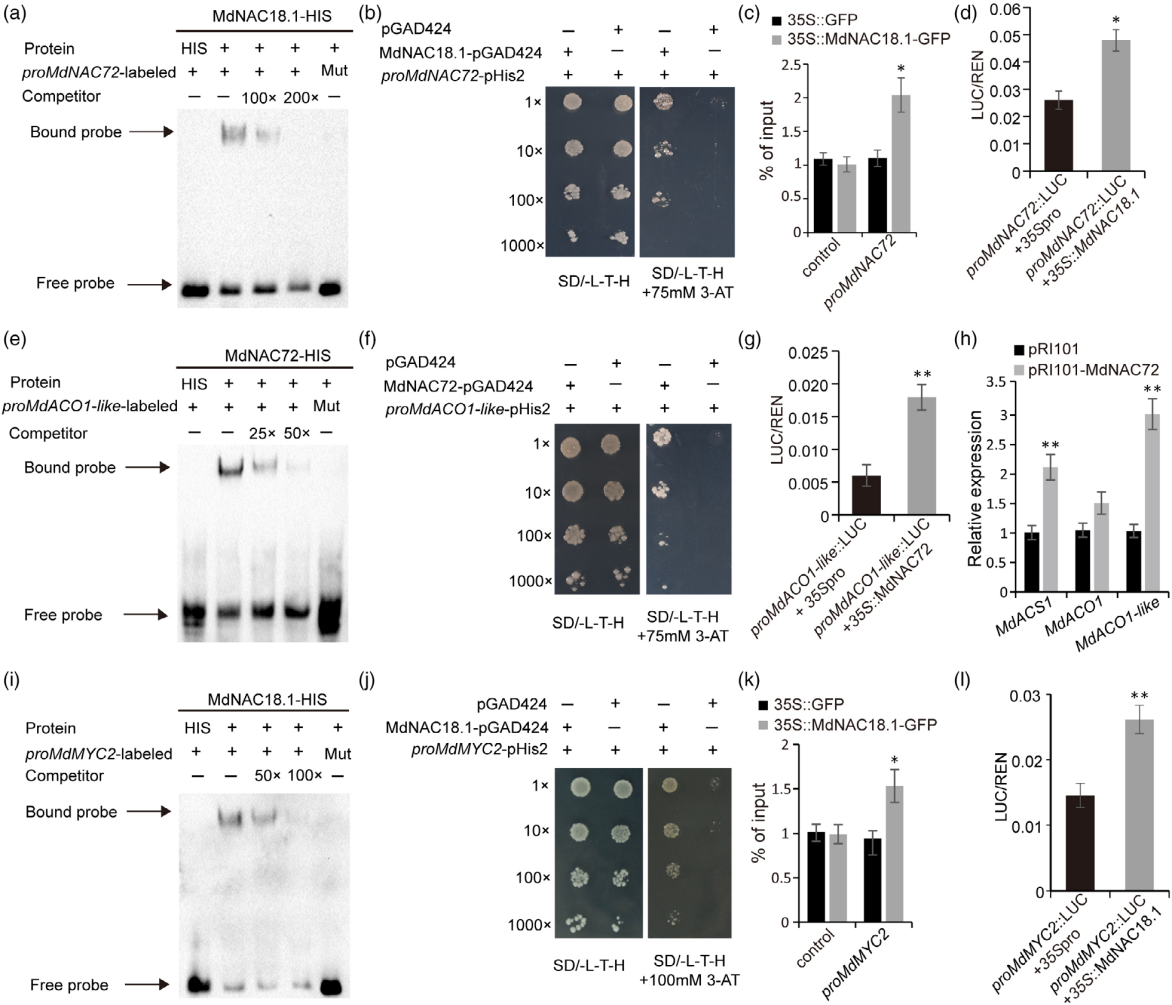
Assay of activation activity of MdNAC18.1 on ripening‐related genes in apple. (a) EMSA assay showed MdNAC18.1 binds to the promoter of *MdNAC72* in vitro. The recognition motif is ‘TGCACGT’, and the mutated version is ‘AAAAAAA’. (b) Analysis of the interaction between MdNAC72 and the *MdACS1* promoter using Y1H. (c) ChIP‐PCR assays showing that MdNAC18.1‐GFP facilitates the enrichment of promoter fragments of *MdNAC72*. Unbound probe was used as the control. (d) Assay of activation effect of MdNAC18.1 on the *MdNAC72* promoter using dual‐luciferase assay. (e) EMSA assay showed that MdNAC72 protein binds to the promoter of *MdACO1‐like* in vitro. The recognition site is ‘TACACG’, and mutated recognition site is ‘AAAAAA’. (f) Analysis of the interaction between MdNAC72 and the *MdACO1‐like* promoter using Y1H. (g) Assay of activation activity of MdNAC72 on the *MdACO1‐like* promoter using dual‐luciferase assay. (h) The expression of ripening‐associated genes in apple callus overexpressing *MdNAC72*. (i) EMSA assay showed that MdNAC18.1 directly binds to the promoter of *MdMYC2* in vitro. The recognition motif is ‘TCACG’, and the mutated version is ‘AAAAA’. (j) Analysis of the interaction between MdNAC18.1 and the *MdMYC2* promoter using Y1H. (k) ChIP‐PCR assays showing that MdNAC18.1‐GFP facilitates the enrichment of promoter fragments of *MdMYC2*. Unbound probe was used as the control. (l) Assay of activation activity of MdNAC18.1 on the *MdMYC2* promoter using dual‐luciferase assay.

*MdNAC18.1* along with *MD03G1222700* designated *MdNAC72* are closely located within a 40‐kb region. MdNAC18.1 and MdNAC72 are orthologs of two adjacent NAC genes, *PpNAC1* and *PpNAC5* (Figure S5), respectively, which are both involved in the regulation of maturity date in peach (Zhang *et al*., 2024). Since the NACBS1 motif was identified in the *MdNAC72* promoter (Figure S3), we investigated whether MdNAC18.1 could regulate transcription of *MdNAC72*. EMSA results showed that MdNAC18.1 was able to bind the NACBS1 recognition site in the *MdNAC72* promoter (Figure 4a), and the binding capacity of MdNAC18.1 to the *MdNAC72* promoter was confirmed by Y1H assay (Figure 4b). ChIP‐qPCR assays revealed a significant enrichment of the promoter fragments of *MdNAC72* by immuneprecipitation (Figure 4c). These results indicated that MdNAC18.1 could bind to the *MdNAC72* promoter. The dual luciferase assay demonstrated that MdNAC18.1 was able to activate the *MdNAC72* promoter (Figure 4d). Subsequently, we tested whether MdNAC72 could activate transcription of *MdACO1‐like* or *MdACS1*. Both EMSA and Y1H assays indicated the binding affinity of MdNAC72 to the *MdACO1‐like* promoter (Figure 4e, f). The dual luciferase assay showed that MdNAC72 had activation effect on the *MdACO1‐like* promoter (Figure 4g). Moreover, the expression of *MdACS1* and *MDACO1‐like* was significantly upregulated in apple calli overexpressing *MdNAC72* (Figure 4h). Silencing *MdNAC72* in transgenic apple callus resulted in a significant reduction in ethylene production, as well as downregulation of ripening genes such as *MdACO1* and *MdACS1* (Figure S6). These results suggested that MdNAC18.1 acts upstream of ripening‐related MdNAC72.

MdMYC2, a jasmonate‐activated transcription factor, regulates fruit ripening by activating ethylene biosynthetic genes in apple (Li *et al*., 2017). The NACBS1 motif was identified in the *MdMYC2* promoter (Figure S4), which prompted us to investigate the relationship between MdNAC18.1 and MdMYC2. EMSA results showed that MdNAC18.1 could bind to the NACBS1 recognition site in the *MdMYC2* promoter (Figure 4i), and Y1H assay validated its binding affinity to the *MdMYC2* promoter (Figure 4j). ChIP‐qPCR assays showed a significant enrichment of the promoter fragments of *MdMYC2* by immuneprecipitation (Figure 4k), suggesting that MdNAC18.1 could bind to the *MdMYC2* promoter. The dual luciferase assay showed that MdNAC18.1 had activation activity on the *MdMYC2* promoter (Figure 4l). Moreover, the expression of *MdMYC2* was significantly up‐regulated in apple calli overexpressing *MdNAC18.1* (Figure S1). These results suggested that MdNAC18.1 acts as molecular node integrating ethylene and jasmonate signalling by interacting with MdMYC2.

### A 58‐bp InDel in the promoter of *MdNAC18.1* is associated with maturity date in apple

**Figure 5:**
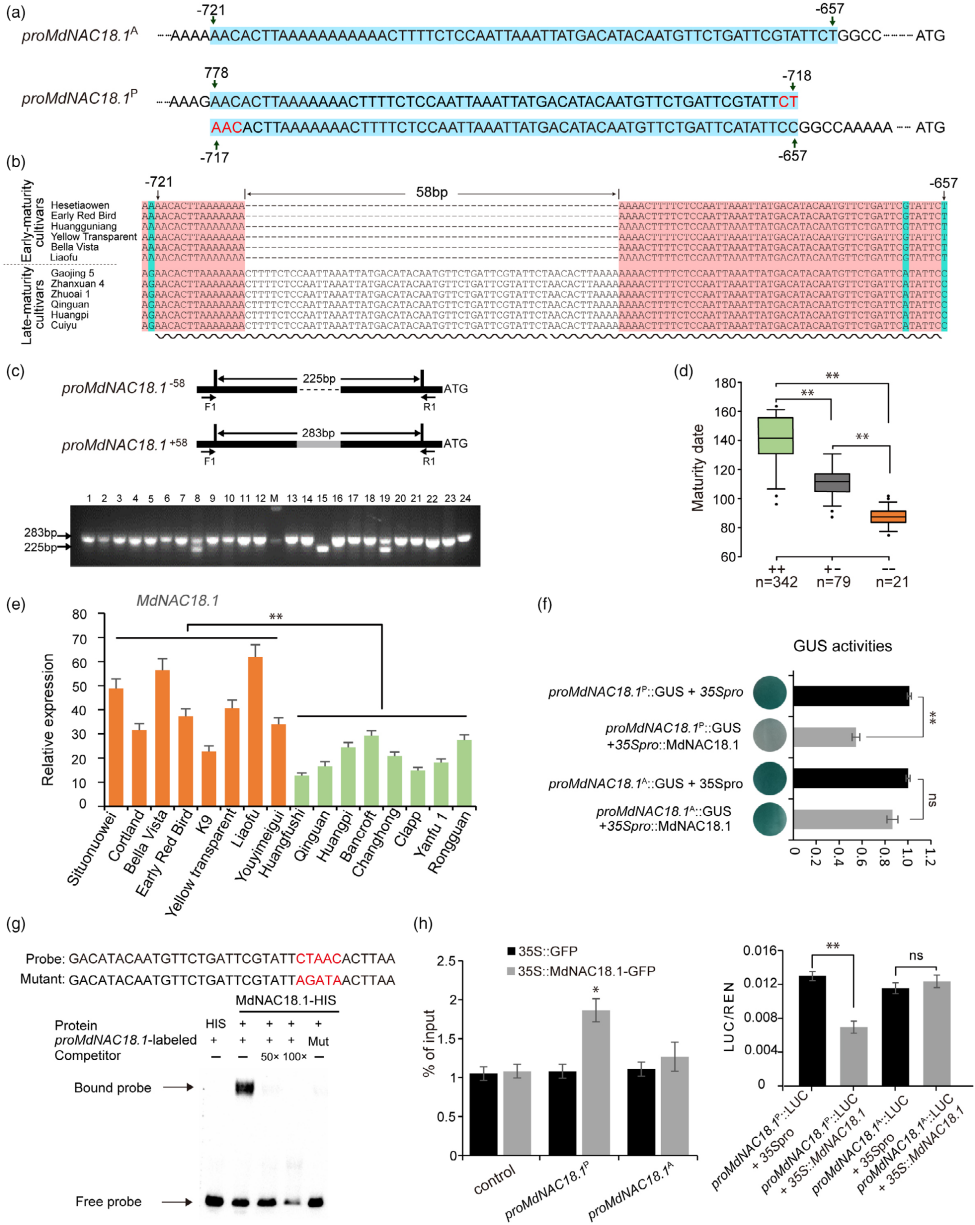
Duplication of a 61‐bp promoter fragment endows MdNAC18.1 with negative transcriptional autoregulation in apple. (a) The promoter structure of two *MdNAC18.1* alleles, *MdNAC18.1* ^A^ and *MdNAC18.1* ^P^. *proMdNAC18.1* ^P^ indicates the *MdNAC18.1* ^P^ promoter containing a 61‐bp fragment duplication, while *proMdNAC18.1* ^A^ represents the *MdNAC18.1* ^A^ promoter contains a 64‐bp fragment that is the counterpart of the 61‐bp fragment. The duplicated unit in *proMdNAC18.1* ^P^ and its counterpart in *proMdNAC18.1* ^A^ are highlighted in the blue background. The MdNAC18.1‐binding motif detected in *proMdNAC18.1* ^P^ is highlighted in red colour. (b) An InDel in the *MdNAC18.1* promoter. The dashed lines represent the 58‐bp deletion, while the wavy lines indicate the 61‐bp duplication. SNPs are indicated with the blue background. (c) An example of genotyping of apple accessions using a STS marker developed from the 58‐bp InDel in the *MdNAC18.1* promoter. F1 and R1 represent a pair of primers flanking the 58‐bp InDel. *proMdNAC18.1* ^−58^ and *proMdNAC18.1* ^+58^ indicate the absence and presence of the 58‐bp insertion, respectively. (d) Boxplots for maturity date based on the 58‐bp InDel in the *MdNAC18.1*promoter. In the box plots, box limits are the upper and lower quartiles with the median values shown by bold lines. Whiskers denote 1.5 × interquartile range and outliers are shown with solid black dots. The symbol “++” and “−−” indicates both alleles carrying the 58‐bp insertion or the 58‐bp deletion, respectively, while the symbol “+−” indicates heterozygosity at the 58‐bp InDel locus. (e) The expression of *MdNAC18.1* in apple fruits carrying either the 58‐bp insertion or the 58‐bp deletion at the ripening stage. (f) The GUS assay showing the binding affinity of MdNAC18.1 to promoters of *proMdNAC18.1* ^A^ and *proMdNAC18.1* ^P^. (g) EMSA assays showed that MdNAC18.1 specifically recognizes the CTAAC motif that was arisen from the 61‐bp promoter fragment duplication. (h) ChIP‐PCR assays showing that MdNAC18.1‐GFP facilitates the enrichment of promoter fragments of *proMdNAC18.1* ^P^, instead of *proMdNAC18.1* ^A^. (i) Assay of activation activity of MdNAC18.1 on the promoters of *proMdNAC18.1* ^A^ and *proMdNAC18.1* ^P^ using dual‐luciferase assay. Error bar means the SD values of three biological replicates. Asterisks indicate the statistical significance based on Student's *t*‐test. **P < 0.01; *P < 0.05; ns, no significance.

*MdNAC18.1* showed significantly higher levels of expression in early‐maturing apple cultivar throughout fruit development than in medium‐ and late‐maturing apple cultivars (Figure S7). To elucidate the mechanism underlying the transcriptional regulation of *MdNAC18.1*, a 2.0‐kb fragment upstream of its translational start codon was isolated and sequenced from early‐ and late‐maturing cultivars. Interestingly, a 61‐bp fragment duplication 718‐bp upstream of the *MdNAC18.1* translational start codon was identified in late‐maturing cultivars (Figure 5a). The 61‐bp fragment was the counterpart of a 64‐bp fragment in early‐maturing. For easy description, the *MdNAC18.1* alleles with or without the 61‐bp promoter fragment duplication were termed *MdNAC18.1* ^P^ and *MdNAC18.1* ^A^, respectively. Alignment of the promoter sequences of early‐ and late‐maturing cultivars revealed a 58‐bp InDel (Figure 5b). Based on the InDel, a sequence‐tagged‐site (STS) marker was developed to genotype the above‐mentioned apple collection. Two polymorphic bands of 283 and 225 bp were detected in all tested accessions (Figure 5c; Figure S8). Overall, accessions carrying a single 283‐bp band or a single 225‐bp band had the longer or shorter MD, respectively, while accessions carrying two bands of 283 and 225 bp had medium MD (Figure 5d). Majority (92.3%) of all tested late‐maturing accessions were homozygous for the 58‐bp insertion, while the homozygosity for the 58‐bp insertion was not found in early‐maturing cultivars (Table S4). These results indicated an association of the 58‐bp InDel in the *MdNAC18.1* promoter with maturity date in apple.

The expression level of *MdNAC18.1* was significantly higher in fruits of apple cultivars carrying a single 225‐bp band at the ripening stage than in those carrying a single 283‐bp band (Figure 5e). To investigate whether the 61‐bp promoter fragment duplication affects gene expression, the full‐length coding sequence of *MdNAC18.1* was fused to the *proMdNAC18.1* ^A^ or *proMdNAC18.1* ^P^ promoters, resulting in *proMdNAC18.1* ^A^::MdNAC18.1 and *proMdNAC18.1* ^P^::MdNAC18.1 constructs, respectively. Transient transformation assay indicated that the expression of *MdNAC18.1* was significantly induced in apple fruits infiltrated with *proMdNAC18.1* ^A^::MdNAC18.1 compared to the EV infiltration, while no significant induction was observed for the *proMdNAC18.1* ^P^::MdNAC18.1 infiltration (Figure S9). Consistently, ethylene content was significantly higher in apple fruits infiltrated with *proMdNAC18.1* ^A^::MdNAC18.1 than those infiltrated with *proMdNAC18.1* ^P^::MdNAC18.1 or the EV emptor. In addition, the *proMdNAC18.1* ^A^ or *proMdNAC18.1* ^P^ promoters were fused to the *GUS* gene and the constructs were transferred into apple calli. GUS activity was significantly lower in apple calli transformed with *proMdNAC18.1* ^P^::GUS and *35Spro*::MdNAC18.1 than in those transformed with *proMdNAC18.1* ^P^::GUS and the CaMV 35S promoter (Figure 5f). By contrast, there was no significant difference in GUS activity between the *proMdNAC18.1* ^A^::GUS/*35Spro*::MdNAC18.1 and *proMdNAC18.1* ^A^::GUS/*35Spro* infiltration. These results suggested a negative role of the 61‐bp promoter fragment duplication in the *MdNAC18.1* transcription.

### MdNAC18.1 has evolved a negative autoregulation system via the 61‐bp fragment duplication in the promoter region

As mentioned above, MdNAC18.1 functioned as a master regulator as it regulated expression of lots of ripening‐related genes. Since master regulators are often autoregulated to ensure their precise expression patterns (Crews and Pearson, 2009), we investigated whether the 61‐bp promoter fragment duplication has an impact on the autoregulation of *MdNAC18.1* transcription. Interestingly, the GUS activities and the dual luciferase assay showed that MdNAC18.1 had negative activation activity on the *MdNAC18.1* ^*P*^ promoter, but was unable to activate the *MdNAC18.1* ^*A*^ promoter (Figure 5f–i). Then, we investigated whether MdNAC18.1 could bind to its own promoter using EMSA assay with a series of probes developed from the promoter sequences of both *MdNAC18.1* ^*A*^ and *MdNAC18.1* ^*P*^ alleles. The results indicated that MdNAC18.1 was unable to bind the *MdNAC18.1* ^*A*^ promoter. By contrast, MdNAC18.1 could bind to probes containing a consensus sequence CTAAC that were developed from edge sequences of the two 61‐bp fragments (Figure 5a, g). However, this binding capacity was disrupted when the consensus sequence CTAAC was mutated. ChIP‐qPCR assays also showed that a significant enrichment of the *proMdNAC18.1* ^*P*^ fragments by immuneprecipitation (Figure 5h). The findings indicated that MdNAC18.1 exhibited binding affinity towards the fragments of *proMdNAC18.1* ^P^, rather than *proMdNAC18.1* ^A^. These results suggested that MdNAC18.1 had an autosuppression function by binding to an additional NAC recognition site with the core sequence CTAAC in the promoter itself which arose from the 61‐bp fragment duplication in apple.

We checked the CTAAC motif associated with the 61‐bp duplication in all 461 apple accessions tested. No mutation was found in the CTAAC motif of the *MdNAC18.1* ^*P*^ allele in all tested accessions. Then, we investigated whether the *MdNAC18.1* gene has undergone selection during apple domestication and improvement. Selective sweep analysis revealed a strong selection on *MdNAC18.1* during apple improvement, but selection signatures were not detected during apple domestication (Figure S10), consistent with the fact that most apple cultivars are of the later‐ripening type.

## Discussion

Identifying causal variants underpinning economically important traits is a crucial step for advancing the application of molecular breeding tools to accelerate breeding cycles in perennial fruit trees that is characterized by a long juvenile phase. In apple, maturity date is closely related to fruit taste and softening, with fruits of late‐ripening cultivars tending to have more sweetness and slower softening (Nybom *et al*., 2012; Watts *et al*., 2023). Therefore, maturity date is of considerable importance in apple and its genetic basis has been extensively investigated. Increasing evidence shows that *MdNAC18.1* is the curial major gene for maturity date in apple, but causal variant(s) underpinning maturity date variation remain unclear. A non‐synonymous SNP at the fifth amino acid position in MdNAC18.1 was first reported as a candidate causal variant for maturity date (Migicovsky *et al*., 2016). However, recent studies suggest that mutations in the promoter region that affect the transcription of *MdNAC18.1*, instead of non‐synonymous mutations in the protein coding region, are likely the causal variants for maturity date (Davies and Myles, 2023; Watts *et al*., 2023). In this study, our results indicate that a regulatory variant which arose from a 61‐bp fragment duplication in the promoter of *MdNAC18.1* negatively autoregulates its own expression, which is likely responsible for the delay in maturity date. In peach, activation of *PpNAC1* associated with maturity date at the exponential growth and ripening stages due to epigenetic modifications is responsible for the early‐ripening phenotype (Zhou *et al*., 2023). Similarly, up‐ and down‐regulation of strawberry *FaRIF*, an ortholog of *MdNAC18.1*, cause accelerated or delayed fruit ripening, respectively (Martin‐Pizarro *et al*., 2021). Thus, the regulation of MD‐related NAC TFs at the transcriptional level rather than at the post‐transcriptional level may play a more important role in maturity date of fleshy fruit crops.

**Figure 6:**
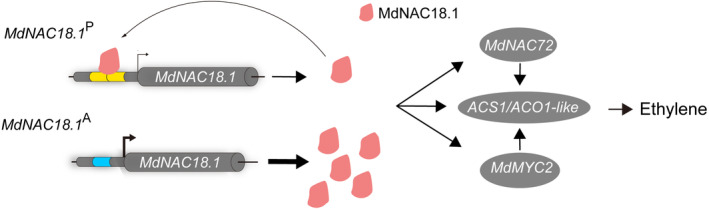
A proposed model for the regulatory role of MdNAC18.1 in maturity date in apple. MdNAC18.1 regulates maturity date by activating transcription of ripening‐related genes, including ethylene biosynthetic genes *MdACS1* and *MdACO1‐like* as well as ethylene production regulator MdNAC72 and MdMYC2 that is a fundamental regulator of the JA signalling. The *MdNAC18.1* gene has two alleles *MdNAC18.1* ^P^ and *MdNAC18.1* ^A^, with the former containing a 61‐bp promoter fragment duplication. MdNAC18.1 is unable to bind the promoter of the *MdNAC18.1* ^A^ allele. However, MdNAC18.1 can act as a negative autoregulating repressor through binding to the CTAAC motif that was arisen from the 61‐bp promoter fragment duplication in the *MdNAC18.1* ^P^ allele, leading to a delay in maturity date in apple. The yellow column in the *MdNAC18.1* promoter represents the 61‐bp duplication fragment, which is the counterpart of a 64‐bp fragment indicated with the blue column.

Ripening‐related NAC TFs mediate a cascade of ripening traits through activating transcription of genes related to ethylene biosynthesis, cell wall degradation, organic acid degradation as well as sugar and aroma accumulation (Cao *et al*., 2021; Zhang *et al*., 2024). Here, MdNAC18.1 was found capable of activating transcription of ethylene biosynthetic genes, *MdACO1‐like* and *MdACS1* that is a key gene controlling fruit firmness (Sunako *et al*., 1999). Like the peach ortholog of PpNAC1, MdNAC18.1 is able to induce transcription of its adjacent MdNAC72 that regulates expression of *MdACO1‐like*. Notably, MdNAC18.1 can activate transcription of the main regulator of JA signalling MdMYC2 that is involved in the regulation of fruit ripening (Li *et al*., 2017). Moreover, MdNAC18.1 has pleiotropic effects on fruit juiciness, sweetness and firmness (Watts *et al*., 2023). These results indicate that NAC18.1 controls large numbers of genes, suggesting it acts as a master regulator to orchestrate the overall tempo of fruit ripening. Master regulators are often autoregulated because their expression levels must be fine‐tuned (Crews and Pearson, 2009). To fulfil its master regulatory role in fruit ripening, MdNAC18.1 evolved an autosuppression module that arose from a duplication of 61‐bp fragment in the promoter region. This duplication resulted in an alternative NAC recognition site containing the core sequence CTAAC, which is enough to drive the binding of MdNAC18.1 to the promoter itself. Thus, MdNAC18.1 can act as a negative autoregulating repressor by binding to the CTAAC motif to repress its own expression, leading to a delay in maturity date. In the absence of the CTAAC motif, MdNAC18.1 is unable to bind its own promoter, and functions as a positive regulator of maturity date through activating transcription of ethylene biosynthetic genes and ripening‐related TFs such as MdNAC72 and MdMYC2. Based on the functional difference of MdNAC18.1 alleles, we propose a model for maturity date variation in apple (Figure 6). Ethylene biosynthetic genes have been reported to play key roles in fruit firmness (Schaffer *et al*., 2007). Our results showed that MdNAC18.1 is capable of activating transcription of ethylene biosynthetic genes. Therefore, autosuppression function of *MdNAC18.1* that ensures lower levels of ethylene production during the process of fruit ripening seems to be responsible for the association of the later‐ripening phenotype with fruit slower softening and long‐term storage in apple.

NAC recognition sequence is characterized as containing the CATGT or CACG core motif in plants (Tran *et al*., 2004). Here, the CACG core motif rather than the CATGT core motif was identified for MdNAC18.1 binding based on DAP‐seq data. EMSA assays revealed an additional motif of CTAAC that was recognized and bound by MdNAC18.1. These results are consistent with the previous report that each NAC TF probably has unique binding sequences (Lindemose *et al*., 2014). If this finding is true, more attention is needed to identify the binding sequence for NAC TFs. In addition, the 61‐bp duplicated sequence related to the formation of the CTAAC motif was blasted against the apple reference genome (GDDH13 V1.1) and significant hits were detected only in the *MdNAC18.1* locus. This suggests that the 61‐bp fragment belongs to non‐repetitive sequence and it has occasionally duplicated at its resident site, which could be an explanation as to why the CTAAC motif was not identified in DAP‐seq data.

Although NAC TFs are well known to act as positive or negative regulators of plant disease resistance (Guo *et al*., 2017), they usually function as positive regulators of fruit ripening. In this study, our results show that MdNAC18.1 has evolved a negative autoregulation system, thus, it can function as negative regulator of maturity date. To our knowledge, this is the first report of negative regulatory role of NAC TFs in fruit development and ripening. Moreover, autoregulation function has seldom been reported in NAC or other TFs associated with fruit traits. A well‐known autoregulatory module is associated with the red flesh trait in apple, in which multiple duplication of a 23‐bp sequence results in positive transcriptional autoregulation of *MdMYB10*, the key regulator of anthocyanin biosynthesis (Espley *et al*., 2009). In this study, we found a negative autoregulation system in another important trait in apple, e.g., maturity date. Intriguingly, MdNAC18.1 acts as positive regulator by binding to NACBS1/5, while it changes to be negative regulator when binding to the CTAAC motif in the promoter itself. More studies are needed to address the role of NAC recognition motifs in the modulation of NAC transcription function.

Maturity date encompasses fruit set, enlargement and ripening. Here, our results showed that *MdNAC18.1* is the crucial major gene for MD in apple and it fulfils its regulatory role in MD through fine‐tuning ethylene biosynthesis. Increasing evidence demonstrates that the low concentration of ethylene plays a critical role in fruit growth of tomato (Huang *et al*., 2022; Sharma *et al*., 2021). In apple, a later‐ripening bud sport ‘Autumn Gala’ shows slower fruit growth compared to its parent ‘Gala’ (Ban *et al*., 2022). The influence of changes in maturity date on fruit growth rate has also been reported in peach (Zhang *et al*., 2024). Therefore, fruit growth rate may have an important role in determining the MD phenotype. In peach, MD‐related NAC TFs have been shown to play regulatory roles in fruit enlargement by activating transcription of genes associated with cell elongation. Thus, whether and how MdNAC18.1 regulates fruit enlargement is worthy of further study.

In conclusion, our study reveals a pivotal role of MdNAC18.1 in the regulation of fruit maturity date in apple. MdNAC18.1 evolved a transcriptional autosuppression module via a 61‐bp sequence duplication in the promoter, leading to a delay in maturity date. Our results provide valuable insights into the genetic architecture controlling maturity date, and are also useful for genetic improvement of fruit quality via marker assisted selection and/or gene editing.

## Materials and methods

### Plant materials

All apple accessions used in this study are maintained at the Institute of Pomology of the Chinese Academy of Agricultural Sciences, Xingcheng, Liaoning province. Maturity date (MD) was determined by computing the interval (in days) between flowering onset and harvest date. Harvest time was determined by the following factors, including no increase in fruit size, the disappearance of background colour along with fruit blush, brown seed colour, and historical records of maturity date. Apple fruits used for transcriptome analysis and quantitative real‐time PCR (qRT‐PCR) were harvested at the expanding and ripening stages. Fruit samples of each accession had three replicates, with each containing 3–5 fruits. Fruit samples were promptly peeled, cored, diced, frozen in liquid nitrogen, and then stored at −80 °C until use. Leaf samples were frozen in liquid nitrogen and stored at −80 °C for subsequent experiments.

### Plasmid construction and transformation

The full‐length coding sequences without the terminal codon of *MdNAC18.1* were cloned from apple ‘Fuji’ and then inserted into the vector pRI101 using ClonExpress II One Step Cloning Kit C112 (Vazyme, Nangjing, China). The specific 350‐bp partial sequences of *MdNAC18.1* were cloned into the pTRV2 vector. The *pRI101‐MdNAC18.1* and *TRV‐MdNAC18.1* plasmids were transformed into *Agrobacterium tumefaciens* (strain *GV3101*) for transformation in apple or tomato ‘Ailsa Criag’. The 2‐week‐old calli induced from apple ‘Orin’ were used for transformation following the previously reported method (An *et al*., 2018). Tomato transformation was performed using *Agrobacterium*‐mediated transformation system (Gao *et al*., 2020). For transient expression assay in apple, the fruits of ‘Gala’ were bagged with perforated polyethylene bags at 30 DAFB (days after full bloom), debagged at 100 DAFB, and then subjected to *Agrobacterium*‐mediated transient transformation according to previously reported method (An *et al*., 2018). After infiltration, apple fruits were stored in an incubator at 24 °C with a light intensity of 100 μmol/m^2^/s. Seven days after infiltration, the flesh tissues around the injection sites were sampled. All the primers used for plasmid construction are listed in Table S5.

### GWAS analysis and detection of selection signatures

GWAS analysis for maturity date was conducted using a collection of 461 apple accessions with 13 603 253 high‐quality single nucleotide polymorphism (SNPs). To identify candidate genes associated with maturity date, the local linkage disequilibrium (LD) decay was calculated to determine LD blocks that encompassed the top 10 significant SNPs within a GWAS peak. Subsequently, all genes located within the LD blocks were delineated, and their expression profiles were compared based on transcriptome data. The detailed genotyping data of all apple accessions and the methods used for GWAS and detection of selection signatures were described in our previous study (Liao *et al*., 2021).

### DNA affinity purification sequencing (DAP‐seq) and data analysis

DAP‐seq was conducted according to the method described by Bartlett *et al*. (2017). Genomic DNA was extracted using a DNA Extraction Kit (Tiangen, Beijing, China), and the DAP‐Seq gDNA library was prepared following the instructions of the NEXTflex Rapid DNA‐Seq Kit (PerkinElmer, Austin). The coding sequence of *MdNAC18.1* was cloned into a pFN19K HaloTag T7 SP6 Flexi vector to facilitate the binding of proteins to magnetic beads. Subsequently, the MdNAC18.1‐HaloTag fusion protein was expressed using the TNT SP6 High‐Yield Protein Expression System (Promega, Wisconsin, USA). Expressed proteins were purified using Magne Halo Tag Beads (Promega, Madison). The MdNAC18.1‐HaloTag fusion protein and library DNA were co‐incubated in PBS buffer with a slow shaking for 1.5 h at room temperature. Beads were washed three times using the same wash buffer to remove unbound DNA fragments. Finally, beads were incubated at 98 °C for 10 min to denature the MdNAC18.1‐Halo protein and to release the bound DNA fragments. The DNA was sequenced using an Illumina NovaSeq instrument with 100‐bp single‐end reads. The mock DAP‐Seq libraries used as negative control were prepared without the addition of protein to the beads.

The raw sequencing reads underwent preprocessing steps including removal of sequencing adaptors, elimination of bases with low‐quality scores (<20), and filtering out short reads using Trimmomatic (Bolger *et al*., 2014). Subsequently, the cleaned reads were aligned to the apple reference genome GDDH13 v.1.1 using Bowtie 2 (Langmead and Salzberg, 2012) with default parameters. Genes harbouring peaks located within 2 kb upstream of the transcription start site (TSS) or downstream of the transcription termination site (TTS) were defined as target genes. KEGG pathway analysis was conducted using KOBAS 3.0 (Xie *et al*., 2011), while GO analysis was performed using PANNZER2 (Törönen *et al*., 2018). GO and KEGG enrichment analyses were carried out using the ClusterProfiler package v3.10.1 (Yu *et al*., 2012). GO terms and KEGG pathways were considered significantly enriched at a false discovery rate (FDR) of less than 0.05.

### Gene expression analysis and RNA‐seq

Total RNA was extracted using the RNApure Plant Kit (DNase I) (CW0559S, Cwbio, Beijing, China) following the manufacturer's instructions. The first‐strand cDNA was synthesized via PrimeScirptTMRT reagent Kit with gDNA Eraser (RR047A, Takara, Dalian, China). qRT‐PCR was run on the StepOne Plus Real‐Time PCR system (AB, CA, USA) by UltraSYBR Mixture (Low Rox) (CW2610M, Cwbio, Beijing, China). The 18S rRNA was used as an internal control, and the relative expression levels were calculated by 2‐^▵▵CT^ method. Sequences of the primers for qRT‐PCR are shown in Table S5. RNA‐seq data of four apple accessions used in this study were reported in our previous study (Liao *et al*., 2021).

### Ethylene production determination

Ethylene production in apple fruits and calli was measured using previously reported method (Yang *et al*., 2017). Flesh tissues surrounding the infiltrated sites or transgenic calli were collected and put in 15 mL air‐tight containers for 1 h at room temperature. Then, 1 mL headspace gas was injected into the gas chromatography (GC) system, and ethylene production was measured with ethylene standard gas as a control. Ethylene production was normalized based on fruit weight. The measurement was performed with three biological replicatess and each replicate contained at least 3 fruits or callus lines.

### Dual luciferase reporter assay

The full coding sequences of *MdNAC18.1* or *MdNAC72* were amplified and cloned into the *Sal* I‐digested vector *pRI101* to generate effectors. The plasmids *pRI101‐MdNAC18.1* and *pRI101‐MdNAC72* were transformed into *Agrobacterium tumefaciens* (strain *GV3101*). The vector pGreen II‐0800‐LUC was digested using *Hin*d III and *Bam* HI. The promoter sequences of *MdNAC18.1*, *MdACO1‐like*, *MdACS1* or *MdMYC2* were inserted into the vector pGreen II‐0800‐LUC to develop reporters. The constructed plasmids were transformed into *GV3101* strain which contained pSoup‐p19 plasmids. The *Agrobacterium* strain *GV3101* cells which carry individual effector and reporter constructed plasmids were co‐infected into four‐week‐old *Nicotiana benthamiana* leaves at the ratio of 9:1. According to the instructions of Dual‐Luciferase Reporter System (E1960, Promega, Beijing, China), the activities of LUC and REN were tested on Infinite M200 pro (TECAN, Switzerland). Primers used for vector construction are listed in Table S5.

### Protein extraction and purification

The vector pET32A containing His tag was used for protein expression. The protein expression vector pET32A was digested with *Eco*RI and *Sal*I. The coding sequences of *MdNAC18.1* and *MdNAC72* were amplified and inserted into the pET32A vector, resulting recombinant protein expression plasmids. The plasmids were transformed into *Escherichia coli* BL21 (DE3) competent cells (CD601, TransGen Biotech, Beijing, China). Then, cells which expressed protein were induced at 16 °C for 14–16 h with 0.5 mM isopropyl β‐D‐1‐thiogalactopyranoside (IPTG). The fusion protein was purified using the His‐Tagged Protein Purification Kit (CW0894S, Cwbio, Beijing, China) according to the manufacturer's protocol. Primers are listed in Table S5.

### Electrophoretic mobility shift assay (EMSA)

The promoter fragments of *MdNAC18.1*, *MdNAC72*, *MdACO1‐like*, and *MdACS1* were amplified and labelled with biotin at the 3′ end using EMSA Probe Biotin Labeling Kit (GS008, Beyotime, Shanghai, China) according to the manufacturer's instructions. The unlabeled and mutated probes were used as competitors. His‐tag protein was used as negative control. Protein‐DNA binding actions and biotin‐labelled DNA detections were carried out using Chemiluminescent EMSA Kit (GS009, Beyotime, Beijing, China). All probe sequences are listed in Table S5.

### Yeast one‐hybrid assay

For yeast one‐hybrid assay, the promoters of *MdACS1*, *MdACO1‐like*, and *MdNAC72* were inserted into the pHIS2 vector. The pGAD424 was used as negative control, and the coding sequence of *MdNAC18.1* was inserted into the pGAD424 vector. The plasmids were co‐transformed into HM4178 yeast strain. Transformed yeast cells were plated on SD‐Trp/‐Leu/‐His and SD‐Trp/‐Leu/‐His containing 3‐AT.

### ChIP‐qPCR

ChIP‐qPCR was performed following a previously reported method (Sun *et al*., 2024). Transgenic apple calli expressing *35S::GFP* and *35S::MdNAC18.1‐GFP* were subjected to ChIP‐qPCR analysis, and anti‐GFP antibodies were used for immunoprecipitation. The experiment followed the instructions from the ChIP assay kit (Beyotime, P2078). The full set of primers used for ChIP‐qPCR is listed in Table S5.

### GUS activity

The promoter sequences of *MdNAC18.1* ^*P*^ and *MdNAC18.1* ^*A*^ were amplified and cloned into the pCAMBIA1391, which carries the GUS reporter gene. The *proMdNAC18.1* ^*P*^ *‐GUS* and *proMdNAC18.1* ^*A*^ *‐GUS* constructs were transformed into *Agrobacterium tumefaciens* (strain *GV3101*). The full coding sequence of *MdNAC18.1* was fused to the *Sal*I‐digested vector pRI101 to generate effector, and the constructed plasmids were transformed into *GV3101* strain. The *Agrobacterium* strain *GV3101* cells carrying effector or reporter were co‐infected into two‐week‐old apple calli at the ratio of 1:1. The staining assay was performed at 37 °C for 3 h after treatment 36 h on MS medium. A GUS staining kit (Solarbio, G2060) was used in this study. GUS activity was detected following the method described by An *et al*. (2018). The complete list of primers used is provided in Table S5.

### Statistical analysis

All statistical analyses were conducted using SPSS Statistics version 17.0 (SPSS Inc., Chicago, IL, USA). One‐way ANOVA followed by Tukey's honestly significant difference test for multiple comparisons or Student's *t* test for two independent samples was used for statistical analysis (*P < 0.05 and **P < 0.01).

## Author contributions

L.L., Y.H. and J.‐P.A. planned and designed the experiments. B.Z., Q.Y., H.‐F.L., T.‐T.Z. and L.‐L.Z. performed experiments. X.‐F.W., L.L. and W.‐H.Z. performed data analysis. L.L., B.Z. and Y.H. wrote the manuscript. Q.‐M.G., C.‐X.Y. and Y.H. revised the manuscript.

## Conflict of interest

None declared.

## Supporting information


**Figure S1.** Expression levels of ethylene biosynthesis‐related genes in apple fruits and calli transformed with *MdNAC18.1*. (a) Relative expression of *MdACS1, MdACO1, MdNAC72* and *MdMYC2* in *MdNAC18.1‐*overexpressing or *MdNAC18.1‐*silenced calli of apple ‘Orin’. (b) Relative expression of *MdACS1, MdACO1, MdNAC72* and *MdMYC2* in *MdNAC18.1‐*overexpressing or *MdNAC18.1‐*silenced apple fruits. Transgenic fruits were developed using transient transformation assay. Empty vectors of *pRI101* or *pTRV* were used as the control. Error bar means the SD values of three biological replicates. Asterisks indicate the statistical significance based on Student's *t*‐test. *P < 0.05, **P < 0.01.
**Figure S2.** Functional annotation of potential target genes of MdNAC18.1 that were identified based on DAP‐seq. (a) Gene ontology analysis. (b) Kyoto Encyclopedia of Genes and Genomes (KEGG) analysis.
**Figure S3.** Functional analysis of *MdACO1‐like* in transgenic apple calli. (a) Overexpressing *MdACO1‐like* in ‘Orin’ apple calli. (b) Ethylene content in transgenic apple calli. The empty vector of *pRI101* was used as control. Error bar means the SD values, and asterisks represent significant differences based on Student's *t*‐test. *P < 0.05, **P < 0.01.
**Figure S4.** The distribution of NAC‐binding sites (NACBS) containing the core sequence CACG in the promoters of ripening‐related genes *MdNAC18.1*, *MdACS1*, *MdNAC72*, and *MdMYC*.
**Figure S5.** Phylogenetic tree of MdNAC18.1 protein and their homologs in other species, including peach, tomato, strawberry and *Arabidopsis thaliana*. Bootstrap values are indicated at the nodes of the branches. MdNAC18.1 in apple is highlighted in red color.
**Figure S6.** Functional analysis of *MdNAC72* in transgenic apple calli. (a) Silencing *MdNAC72* in ‘Orin’ apple calli. (b) Ethylene content in transgenic apple calli. (c) The expression levels of ripening‐related genes in NAC72‐silenced transgenic callus. The empty vector of *pTRV* was used as control. Error bar means the SD values, and asterisks represent significant differences based on Student's *t*‐test. *P < 0.05, **P < 0.01.
**Figure S7.** Relative expression of *MdNAC18.1* in fruits at three stages of early‐maturing, medium‐maturing and late‐maturing cultivars. S1 to S3 represent the fruitlet, expanding, and ripening stages, respectively. The early‐maturing, medium‐maturing and late‐maturing cultivars used in the analysis are ‘Genera Early’, ‘Golden Delicious’ and ‘Indo’. Significant differences are indicated by different lowercase letters based on One‐way ANOVA followed by Tukey's honestly significant difference test at *P*<0.05.
**Figure S8.** Genotyping of apple accessions based on the 58‐bp InDel in the *MdNAC18.1* promoters. M, DNA marker 500.
**Figure S9.** Relative expression of *MdNAC18.1* and ethylene content in apple fruits transformed with *MdNAC18* under the control of the *proMdNAC18.1* ^A^ promoter (*proMdNAC18.1* ^A^::MdNAC18.1) or the *proMdNAC18.1* ^P^ promoter (*proMdNAC18.1* ^P^::MdNAC18.1).
**Figure S10.** Local screening for selective sweeps in apple domestication (*M.sieversii* vs. Heirlooms, *M.sylvestris* vs. Heirlooms) and improvement (Heirlooms vs. cultivars). The detailed results of Genome‐wide screening of selective sweeps have been presented in the previous study as mentioned in the material part. Three methods including the nucleotide diversity (pi), Fixation index (fst) and cross‐population composite likelihood ratio (XP‐CLR) were used to identify selective sweeps. The horizontal gray dotted lines indicate genome‐wide thresholds that were estimated based on the top 5% of nucleotide diversity ratios or the top 5% of pi, fst and XP‐CLR values. The yellow blocks represent the candidate regions for maturity date according to GWAS.
**Table S1.** Statistics of the maturity date of 461 apple accessions in this study.
**Table S2.** List of the significantly associated SNPs and genes in chromosome 03 for maturity date in apple fruits.
**Table S3.** List of potential target genes identified by combining the peaks of data from two biological replicates.
**Table S4.** Polymorphic bands around the 58‐bp InDel detected in all tested accessio.
**Table S5.** List of primer sequences used in this study.

## Data Availability

The data supporting the findings of this study are available within the manuscript and its Supporting Information.
