# Supplementary material for: Autosuppression of MdNAC18.1 endowed by a 61‐bp promoter fragment duplication delays maturity date in apple
Source: Plant Biotechnol J. 2025 Feb 26;23(4):1216–29. doi: 10.1111/pbi.14580 (PMC11933844; doi:10.1111/pbi.14580)
Supplement: Supplementary file 1 — Figure S1. Expression levels of ethylene biosynthesis‐related genes in apple fruits and calli transformed with MdNAC18.1. (a) Relative expression of MdACS1, MdACO1, MdNAC72 and MdMYC2 in MdNAC18.1‐overexpressing or MdNAC18.1‐silenced calli of apple ‘Orin’. (b) Relative expression of MdACS1, MdACO1, MdNAC72 and MdMYC2 in MdNAC18.1‐overexpressing or MdNAC18.1‐silenced apple fruits. Transgenic fruits were developed using transient transformation assay. Empty vectors of pRI101 or pTRV were used as the control. Error bar means the SD values of three biological replicates. Asterisks indicate the statistical significance based on Student's t‐test. *P < 0.05, **P < 0.01. Figure S2. Functional annotation of potential target genes of MdNAC18.1 that were identified based on DAP‐seq. (a) Gene ontology analysis. (b) Kyoto Encyclopedia of Genes and Genomes (KEGG) analysis. Figure S3. Functional analysis of MdACO1‐like in transgenic apple calli. (a) Overexpressing MdACO1‐like in ‘Orin’ apple calli. (b) Ethylene content in transgenic apple calli. The empty vector of pRI101 was used as control. Error bar means the SD values, and asterisks represent significant differences based on Student's t‐test. *P < 0.05, **P < 0.01. Figure S4. The distribution of NAC‐binding sites (NACBS) containing the core sequence CACG in the promoters of ripening‐related genes MdNAC18.1, MdACS1, MdNAC72, and MdMYC. Figure S5. Phylogenetic tree of MdNAC18.1 protein and their homologs in other species, including peach, tomato, strawberry and Arabidopsis thaliana. Bootstrap values are indicated at the nodes of the branches. MdNAC18.1 in apple is highlighted in red color. Figure S6. Functional analysis of MdNAC72 in transgenic apple calli. (a) Silencing MdNAC72 in ‘Orin’ apple calli. (b) Ethylene content in transgenic apple calli. (c) The expression levels of ripening‐related genes in NAC72‐silenced transgenic callus. The empty vector of pTRV was used as control. Error bar means the SD values, and asterisks rep [file PBI-23-1216-s001.zip › pbi14580-sup-0001-Figures.docx]

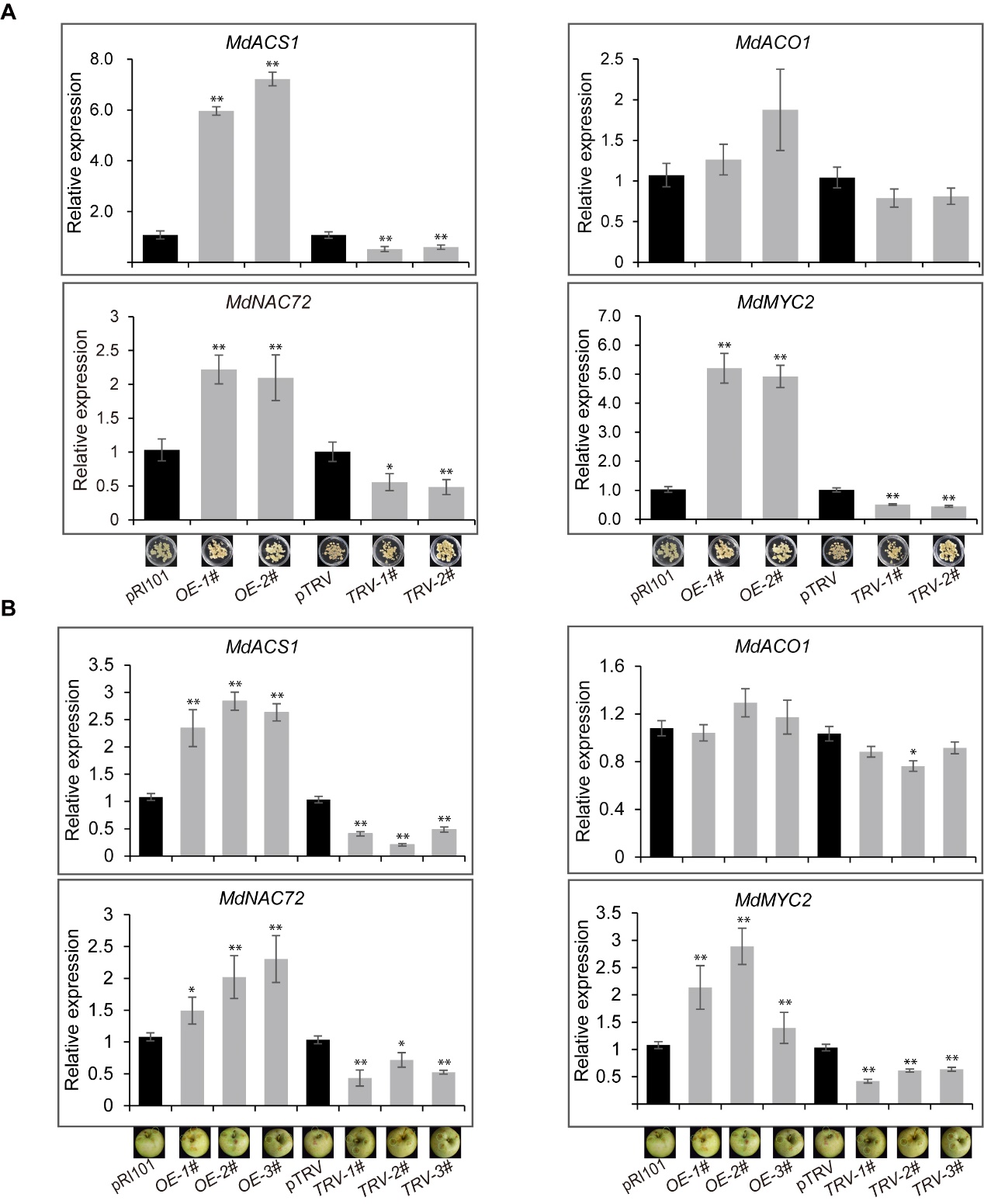


Figure S1. Expression levels of ethylene biosynthesis-related genes in apple fruits and calli transformed with *MdNAC18.1*. (A) Relative expression of *MdACS1, MdACO1, MdNAC72* and *MdMYC2* in *MdNAC18.1-*overexpressing or *MdNAC18.1-*silenced calli of apple ‘Orin’. (B) Relative expression of *MdACS1, MdACO1, MdNAC72* and *MdMYC2* in *MdNAC18.1-*overexpressing or *MdNAC18.1-*silenced apple fruits. Transgenic fruits were developed using transient transformation assay. Empty vectors of *pRI101* or *pTRV* were used as the control. Error bar means the SD values of three biological replicates. Asterisks indicate the statistical significance based on Student’s *t*-test. **P* < 0.05, ** *P* < 0.01.


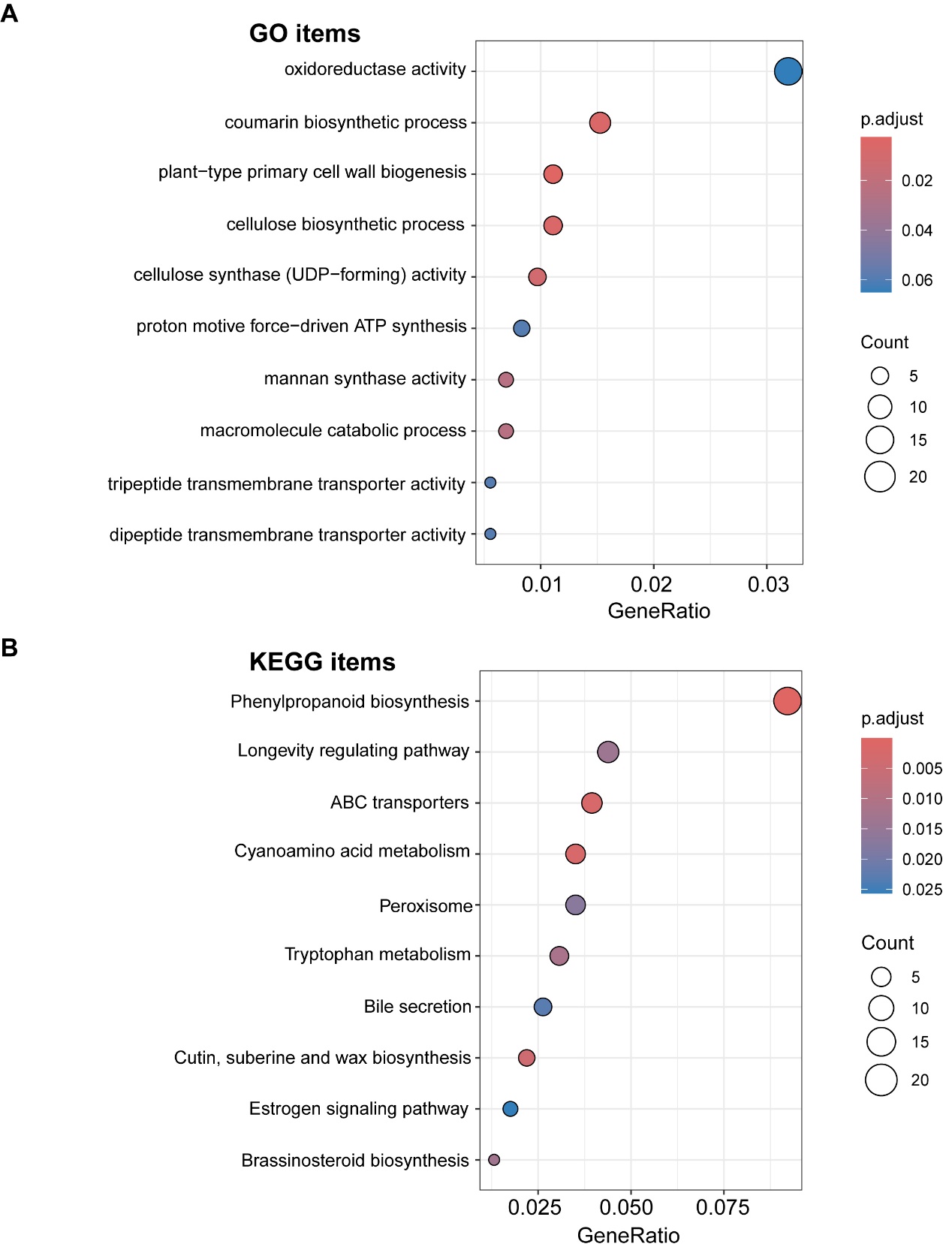


Figure S2. Functional annotation of potential target genes of MdNAC18.1 that were identified based on DAP-seq. (A) Gene ontology analysis. (B) Kyoto Encyclopedia of Genes and Genomes (KEGG) analysis.


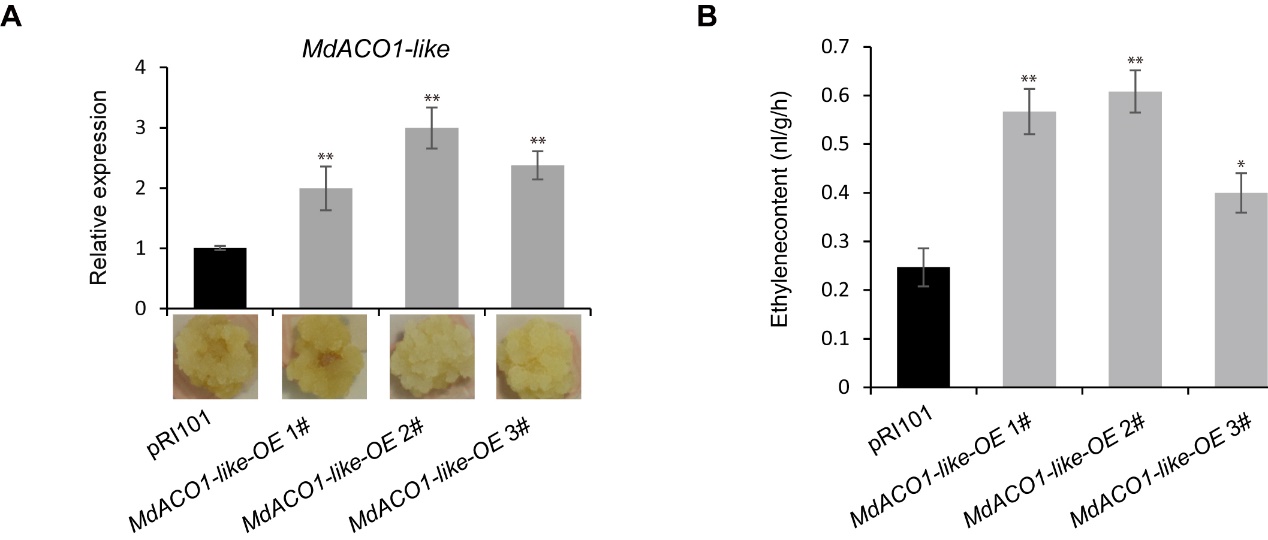


Figure S3. Functional analysis of *MdACO1-like* in transgenic apple calli. (A) Overexpressing *MdACO1-like* in ‘Orin’ apple calli. (B) Ethylene content in transgenic apple calli. The empty vector of *pRI101* was used as control. Error bar means the SD values, and asterisks represent significant differences based on Student’s *t*-test. * *P* < 0.05, ** *P* < 0.01.


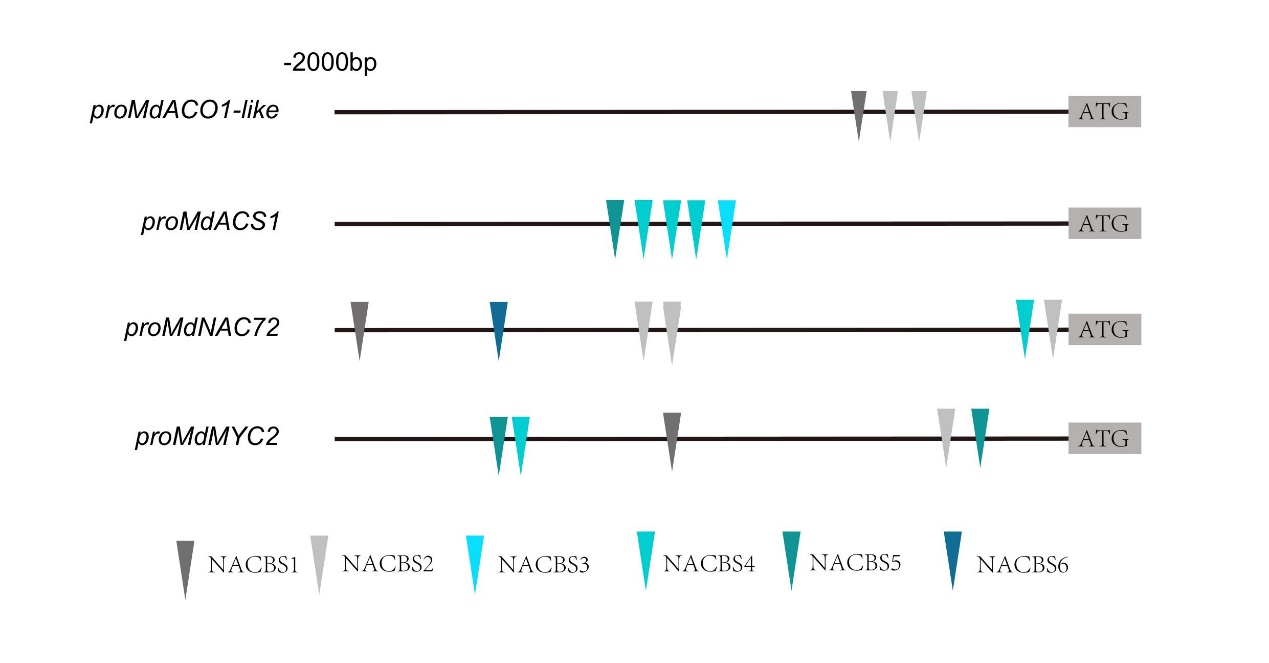


Figure S4. The distribution of NAC-binding sites (NACBS) containing the core sequence CACG in the promoters of ripening-related genes *MdNAC18.1*, *MdACS1*, *MdNAC72*, and *MdMYC*.


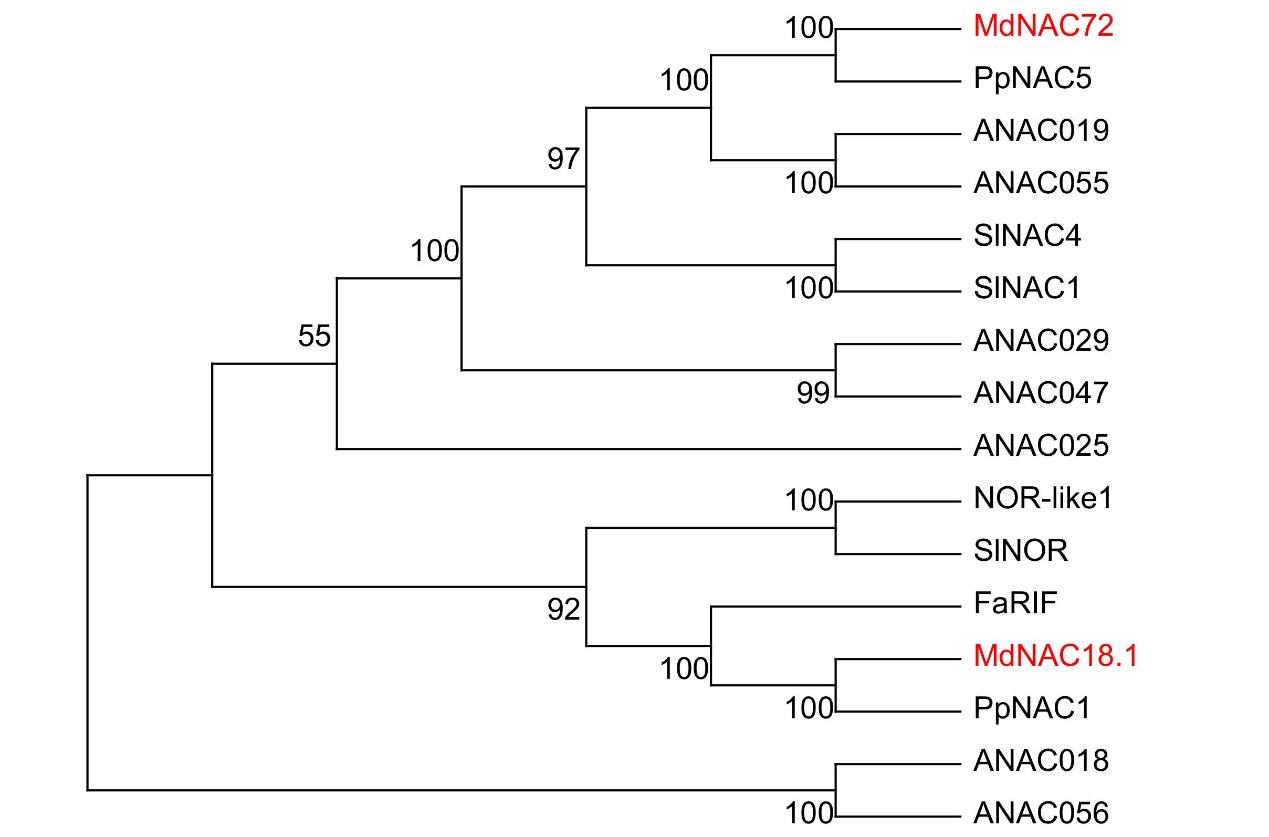


Figure S5 Phylogenetic tree of MdNAC18.1 protein and their homologs in other species, including peach, tomato, strawberry and *Arabidopsis thaliana*. Bootstrap values are indicated at the nodes of the branches. MdNAC18.1 in apple is highlighted in red color.


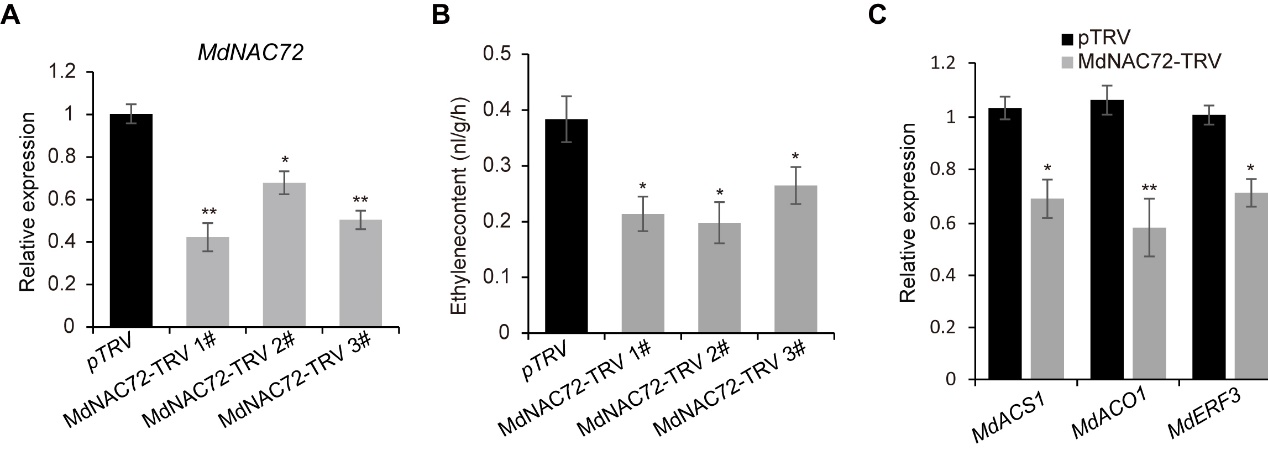


Figure S6. Functional analysis of *MdNAC72* in transgenic apple calli. (A) Silencing *MdNAC72* in ‘Orin’ apple calli. (B) Ethylene content in transgenic apple calli. (C) The expression levels of ripening-related genes in NAC72-silenced transgenic callus. The empty vector of *pTRV* was used as control. Error bar means the SD values, and asterisks represent significant differences based on Student’s *t*-test. * *P* < 0.05, ** *P* < 0.01.


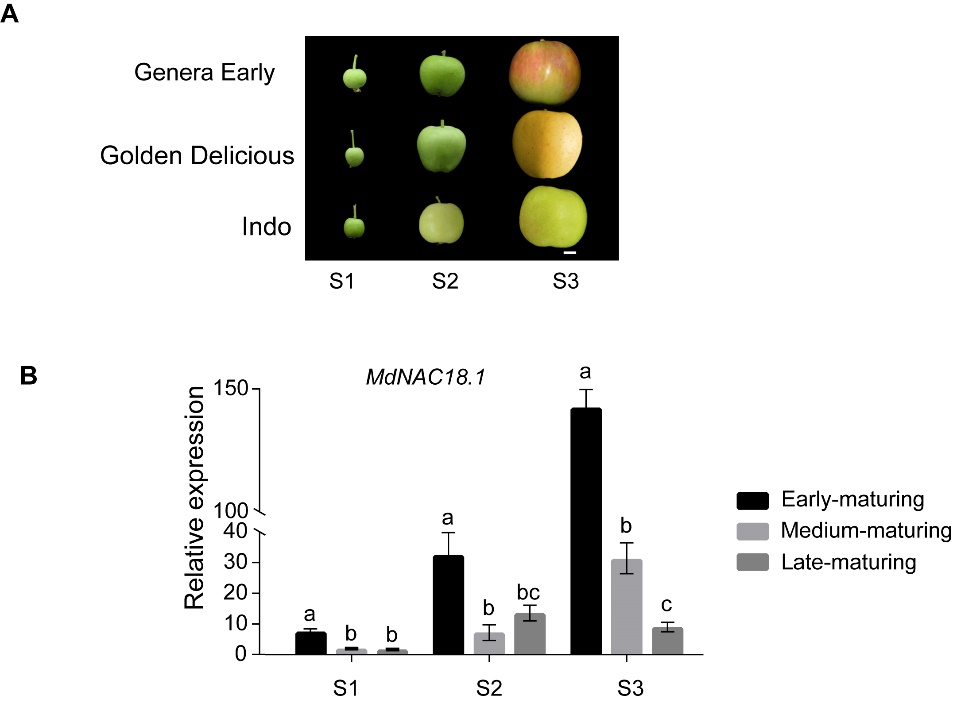


Figure S7. Relative expression of *MdNAC18.1* in fruits at three stages of early-maturing, medium-maturing and late-maturing cultivars. S1 to S3 represent the fruitlet, expanding, and ripening stages, respectively. The early-maturing, medium-maturing and late-maturing cultivars used in the analysis are ‘Genera Early’, ‘Golden Delicious’ and ‘Indo’. Significant differences are indicated by different lowercase letters based on One-way ANOVA followed by Tukey’s honestly significant difference test at *P*＜0.05.


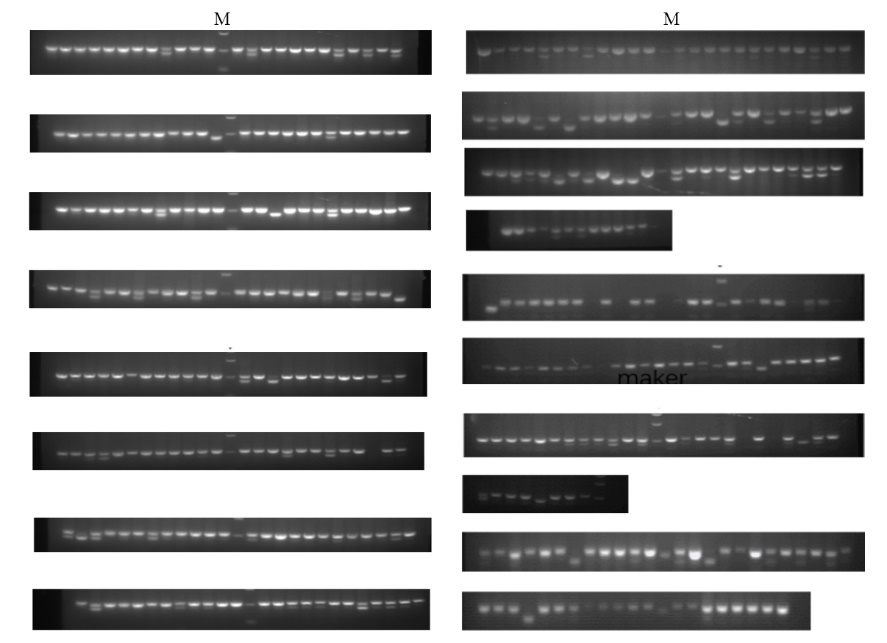


Supplemental Figure 8 Genotyping of apple accessions based on the 58-bp InDel in the *MdNAC18.1* promoters. M, DNA marker 500.


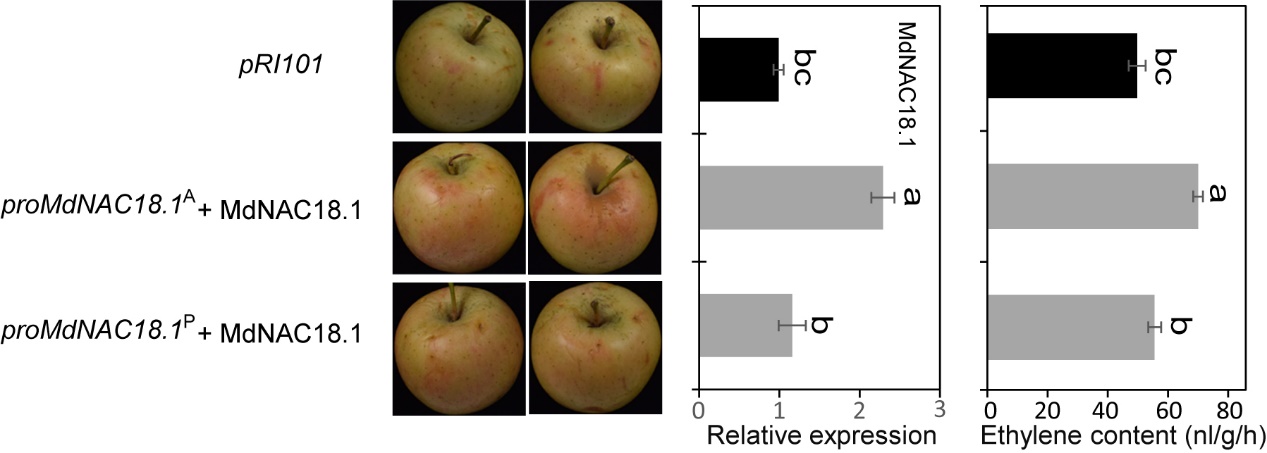
Figure S9. Relative expression of *MdNAC18.1* and ethylene content in apple fruits transformed with *MdNAC18* under the control of the *proMdNAC18.1*^A^ promoter (*proMdNAC18.1*^A^::MdNAC18.1) or the *proMdNAC18.1*^P^ promoter (*proMdNAC18.1*^P^::MdNAC18.1).


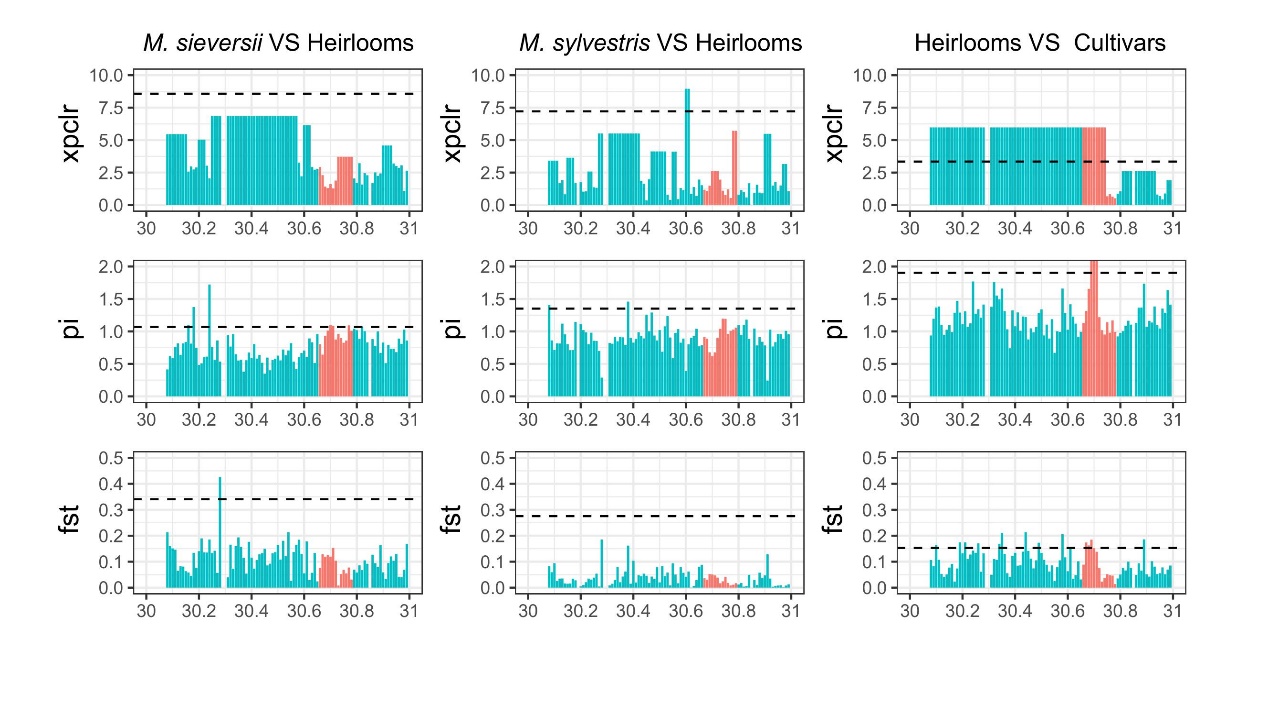


Supplemental Figure 10 Local screening for selective sweeps in apple domestication (*M.sieversii* VS Heirlooms, *M.sylvestris* VS Heirlooms) and improvement (Heirlooms VS cultivars). The detailed results of Genome-wide screening of selective sweeps have been presented in the previous study as mentioned in the material part. Three methods including the nucleotide diversity (pi), Fixation index (fst) and cross-population composite likelihood ratio (XP-CLR) were used to identify selective sweeps. The horizontal gray dotted lines indicate genome-wide thresholds that were estimated based on the top 5% of nucleotide diversity ratios or the top 5% of pi, fst and XP-CLR values. The yellow blocks represent the candidate regions for maturity date according to GWAS.
