# Supplementary material for: Autosuppression of MdNAC18.1 endowed by a 61‐bp promoter fragment duplication delays maturity date in apple
Source: Plant Biotechnol J. 2025 Feb 26;23(4):1216–29. doi: 10.1111/pbi.14580 (PMC11933844; doi:10.1111/pbi.14580)
Supplement: Supplementary file 1 — Figure S1. Expression levels of ethylene biosynthesis‐related genes in apple fruits and calli transformed with MdNAC18.1. (a) Relative expression of MdACS1, MdACO1, MdNAC72 and MdMYC2 in MdNAC18.1‐overexpressing or MdNAC18.1‐silenced calli of apple ‘Orin’. (b) Relative expression of MdACS1, MdACO1, MdNAC72 and MdMYC2 in MdNAC18.1‐overexpressing or MdNAC18.1‐silenced apple fruits. Transgenic fruits were developed using transient transformation assay. Empty vectors of pRI101 or pTRV were used as the control. Error bar means the SD values of three biological replicates. Asterisks indicate the statistical significance based on Student's t‐test. *P < 0.05, **P < 0.01. Figure S2. Functional annotation of potential target genes of MdNAC18.1 that were identified based on DAP‐seq. (a) Gene ontology analysis. (b) Kyoto Encyclopedia of Genes and Genomes (KEGG) analysis. Figure S3. Functional analysis of MdACO1‐like in transgenic apple calli. (a) Overexpressing MdACO1‐like in ‘Orin’ apple calli. (b) Ethylene content in transgenic apple calli. The empty vector of pRI101 was used as control. Error bar means the SD values, and asterisks represent significant differences based on Student's t‐test. *P < 0.05, **P < 0.01. Figure S4. The distribution of NAC‐binding sites (NACBS) containing the core sequence CACG in the promoters of ripening‐related genes MdNAC18.1, MdACS1, MdNAC72, and MdMYC. Figure S5. Phylogenetic tree of MdNAC18.1 protein and their homologs in other species, including peach, tomato, strawberry and Arabidopsis thaliana. Bootstrap values are indicated at the nodes of the branches. MdNAC18.1 in apple is highlighted in red color. Figure S6. Functional analysis of MdNAC72 in transgenic apple calli. (a) Silencing MdNAC72 in ‘Orin’ apple calli. (b) Ethylene content in transgenic apple calli. (c) The expression levels of ripening‐related genes in NAC72‐silenced transgenic callus. The empty vector of pTRV was used as control. Error bar means the SD values, and asterisks rep [file PBI-23-1216-s001.zip › pbi14580-sup-0002-Tables.docx]

| **Supplementary Table 1. Statistics of the maturity date of 461 apple accessions in this study** | | | |
| --- | --- | --- | --- |
| **Sample ID** | **Accession name** | **Botanical varieties** | **Maturity date (d)** |
| 1 | Fengcun fuji | *Malus* × *domestica* | 131 |
| 2 | Honghua | *Malus* × *domestica* | 139 |
| 3 | Gaojin 5 | *Malus* × *domestica* | 165 |
| 4 | Pingyinduanzhi | *Malus* × *domestica* | 138 |
| 5 | Xingcheng 7-7 | *Malus* × *domestica* | 129 |
| 6 | Fushan 1 | *Malus* × *domestica* | 138 |
| 7 | White Winter Pearmain | *Malus* × *domestica* | 164 |
| 8 | Stark Spur Golden Delicious | *Malus* × *domestica* | 138 |
| 10 | Saint Lawrence | *Malus* × *domestica* | 119 |
| 11 | Xinhong | *Malus* × *domestica* | 120 |
| 13 | 1951-3-1 | *Malus* × *domestica* | 151 |
| 14 | Nero 26 | *Malus* × *domestica* | 130 |
| 15 | Shinano Sweet | *Malus* × *domestica* | 111 |
| 16 | Behene | *Malus* × *domestica* | 136 |
| 17 | Honeycrisp | *Malus* × *domestica* | 116 |
| 18 | Huangfushi | *Malus* × *domestica* | 157 |
| 19 | Zhangjiakouduanzhi | *Malus* × *domestica* | 138 |
| 20 | Sishuiduanzhi | *Malus* × *domestica* | 138 |
| 21 | Kelongxieertouming | *Malus* × *domestica* | 126 |
| 22 | 132 | *Malus* × *domestica* | 138 |
| 23 | Huangjin | *Malus* × *domestica* | 121 |
| 24 | Hardi Spur Delicious | *Malus* × *domestica* | 125 |
| 25 | Kahong | *Malus* × *domestica* | 116 |
| 26 | Meize | *Malus* × *domestica* | NA |
| 27 | Laxton's superb | *Malus* × *domestica* | 151 |
| 28 | Atlas | *Malus* × *domestica* | 119 |
| 29 | Priam | *Malus* × *domestica* | 117 |
| 30 | Zhanxuan 4 | *Malus* × *domestica* | 164 |
| 31 | Prime Gold | *Malus* × *domestica* | 134 |
| 32 | Allington | *Malus* × *domestica* | 134 |
| 33 | Qinguan | *Malus* × *domestica* | 168 |
| 34 | Zhuoai 1 | *Malus* × *domestica* | 164 |
| 35 | Qianqiu | *Malus* × *domestica* | 134 |
| 36 | Cardinal | *Malus* × *domestica* | 134 |
| 37 | II10-15 | *Malus* × *domestica* | 81 |
| 39 | Xingcheng 4-21 | *Malus* × *domestica* | 165 |
| 40 | Huangpi | *Malus* × *domestica* | 159 |
| 41 | Bancroft | *Malus* × *domestica* | 170 |
| 42 | Wealthy | *Malus* × *domestica* | 121 |
| 43 | Changhong | *Malus* × *domestica* | 164 |
| 44 | Fenghuangluanhaitangguo | *Malus* × *domestica* | 120 |
| 45 | Enqi | *Malus* × *domestica* | 120 |
| 46 | Qiheduanjinguan | *Malus* × *domestica* | 139 |
| 47 | Kaisaiweilian | *Malus* × *domestica* | 139 |
| 49 | Clapp | *Malus* × *domestica* | 164 |
| 50 | Fushan 5 | *Malus* × *domestica* | 138 |
| 51 | Miller Sturdy Spur Delicious | *Malus* × *domestica* | 112 |
| 52 | Blushing Golden | *Malus* × *domestica* | 164 |
| 53 | Prima | *Malus* × *domestica* | 116 |
| 54 | Zaoqiangduanzhi | *Malus* × *domestica* | 139 |
| 55 | Youlixiang | *Malus* × *domestica* | 143 |
| 56 | Starkrimson(Holland) | *Malus* × *domestica* | 107 |
| 58 | Kangbingjinguan 51 | *Malus* × *domestica* | 134 |
| 59 | k12 | *Malus* × *domestica* | 116 |
| 60 | Changhong | *Malus* × *domestica* | 149 |
| 61 | Yanfu 1 | *Malus* × *domestica* | 165 |
| 62 | Xingcheng 9-23 | *Malus* × *domestica* | 121 |
| 63 | Guoguang_Ⅰ_11-2_ | *Malus* × *domestica* | 165 |
| 64 | Gaoqiu | *Malus* × *domestica* | NA |
| 65 | Mikilife-1 | *Malus* × *domestica* | 141 |
| 66 | Situonuowei | *Malus* × *domestica* | 78 |
| 67 | Topred fuji | *Malus* × *domestica* | 164 |
| 68 | Ningfeng | *Malus* × *domestica* | 165 |
| 69 | Hongxiezi | *Malus* × *domestica* | 164 |
| 70 | Yingjin | *Malus* × *domestica* | 125 |
| 71 | Bo 8 | *Malus* × *domestica* | 134 |
| 72 | Michinoku | *Malus* × *domestica* | 116 |
| 73 | Stark Jumbo | *Malus* × *domestica* | 139 |
| 74 | Aihuang | *Malus* × *domestica* | 164 |
| 75 | Rongguan | *Malus* × *domestica* | 165 |
| 76 | Huamei | *Malus* × *domestica* | 121 |
| 77 | Riskin | *Malus* × *domestica* | 134 |
| 79 | Baumannova reneta | *Malus* × *domestica* | 130 |
| 80 | Royal | *Malus* × *domestica* | 121 |
| 81 | Zhanxuan 16 | *Malus* × *domestica* | 164 |
| 82 | Stark Spur Supreme Red Delicious | *Malus* × *domestica* | 142 |
| 83 | Xinguang | *Malus* × *domestica* | 164 |

| 84 | Lambourne lord | *Malus* × *domestica* | 121 |
| --- | --- | --- | --- |
| 85 | Dongchengguan 13 | *Malus* × *domestica* | 134 |
| 86 | Xingcheng 10-18 | *Malus* × *domestica* | 126 |
| 87 | Cloden | *Malus* × *domestica* | 164 |
| 88 | Zaosheng 16 | *Malus* × *domestica* | 113 |
| 89 | Bo 19 | *Malus* × *domestica* | 130 |
| 90 | Shenglihongguan | *Malus* × *domestica* | 121 |
| 93 | Golden Delicious | *Malus* × *domestica* | 163 |
| 94 | Xiongyue 2 | *Malus* × *domestica* | 165 |
| 96 | Shinano RED | *Malus* × *domestica* | NA |
| 97 | May Queen | *Malus* × *domestica* | 134 |
| 98 | Inflancki | *Malus* × *domestica* | 117 |
| 99 | Hesetiaowen | *Malus* × *domestica* | 90 |
| 100 | Jinshayilamu | *Malus* × *domestica* subsp.chinesnsis. | 134 |
| 101 | Cortland | *Malus* × *domestica* | 85 |
| 102 | Guoqing | *Malus* × *domestica* | 165 |
| 103 | Dabinette | *Malus* × *domestica* | 155 |
| 105 | Xingcheng 27-2 | *Malus* × *domestica* | 165 |
| 106 | Szampion | *Malus* × *domestica* | 165 |
| 107 | Bailuosimalin | *Malus* × *domestica* | 134 |
| 108 | Nanchengaijinguan | *Malus* × *domestica* | 164 |
| 109 | Charles Ross | *Malus* × *domestica* | 134 |
| 110 | American summer pearmain | *Malus* × *domestica* | 117 |
| 112 | Generos | *Malus* × *domestica* | 155 |
| 113 | Meixiang | *Malus* × *domestica* | 92 |
| 114 | Baldwin | *Malus* × *domestica* | 144 |
| 115 | Hadibolaite | *Malus* × *domestica* | 136 |
| 116 | Baldwin | *Malus* × *domestica* | 116 |
| 117 | Red Spur Delicious | *Malus* × *domestica* | 125 |
| 118 | Qindao 1 | *Malus* × *domestica* | 138 |
| 119 | Bianqiangzi 1 | *Malus* × *domestica* | 141 |
| 120 | Miyakiji | *Malus* × *domestica* | 165 |
| 121 | Well Spur Delicious | *Malus* × *domestica* | 138 |
| 123 | De 2 | *Malus* × *domestica* | 126 |
| 124 | Liuyu yabian | *Malus* × *domestica* | 84 |
| 125 | Dajin | *Malus* × *domestica* | 164 |
| 126 | Beifangxinabo | *Malus* × *domestica* | 121 |
| 127 | B-xijinguan | *Malus* × *domestica* | 155 |
| 128 | Bella Vista | *Malus* × *domestica* | 79 |
| 129 | Bismarck | *Malus* × *domestica* | 116 |
| 130 | Juliana | *Malus* × *domestica* | 136 |
| 132 | Qingguang | *Malus* × *domestica* | 164 |
| 133 | Helasang | *Malus* × *domestica* | 134 |
| 134 | Shiai | *Malus* × *domestica* | 164 |
| 135 | Xingcheng 8-2 | *Malus* × *domestica* | 77 |
| 136 | Kelisike | *Malus sieversii* (Ledeb.) M.Roem. | 139 |
| 137 | Weiqinni | *Malus* × *domestica* subsp.chinesnsis. | 130 |
| 138 | Wangling | *Malus* × *domestica* | 143 |
| 139 | Napoleon | *Malus* × *domestica* | 165 |
| 140 | Seedling Schytte | *Malus* × *domestica* | 134 |
| 142 | Jinshiji | *Malus* × *domestica* | 135 |
| 143 | Qihuyihao 1 | *Malus* × *domestica* | 143 |
| 144 | Yoshkee | *Malus* × *domestica* | NA |
| 145 | Xinjiang 1-3 | *Malus* × *domestica* subsp.chinesnsis. | 88 |
| 146 | Golden Spur Delicious | *Malus* × *domestica* | 165 |
| 147 | Peach | *Malus* × *domestica* | 121 |
| 149 | Delicious | *Malus* × *domestica* | 155 |
| 150 | Aiwq | *Malus* × *domestica* | 155 |
| 151 | Melrose | *Malus* × *domestica* | 134 |
| 152 | Doyle | *Malus* × *domestica* | 113 |
| 153 | Kangtunduanzhi | *Malus* × *domestica* | 134 |
| 154 | xinjiang 327 | *Malus* × *domestica* | 138 |
| 155 | Bensuojinguanyouxi | *Malus* × *domestica* | 134 |
| 157 | Fuqiu | *Malus* × *domestica* | 133 |
| 158 | Doyl | *Malus* × *domestica* | 85 |
| 159 | Starkrimson | *Malus* × *domestica* | 134 |
| 160 | Xinhua 1 | *Malus* × *domestica* | 141 |
| 161 | Xinjiangmianpingguo | *Malus* × *domestica* subsp.chinesnsis. | 134 |
| 162 | Mikilife-2 | *Malus* × *domestica* | 116 |
| 163 | Lvshuai | *Malus* × *domestica* | NA |
| 165 | Lodi | *Malus* × *domestica* | 164 |
| 166 | Early red gala | *Malus* × *domestica* | 111 |
| 167 | Danding | *Malus* × *domestica* | 117 |
| 168 | Fujing | *Malus* × *domestica* | 165 |
| 169 | Herrnhut | *Malus* × *domestica* | 139 |
| 170 | Shaguo | *Malus asiatica* Nakai | 103 |
| 172 | Fuhong | *Malus* × *domestica* | 95 |
| 173 | Lobo | *Malus* × *domestica* | 134 |
| 174 | Danxia | *Malus* × *domestica* | 165 |
| 175 | Shidonghaoji | *Malus* × *domestica* | 117 |
| 176 | Early Red Bird | *Malus* × *domestica* | 85 |
| 177 | Simonffy Piros | *Malus* × *domestica* | 116 |

| 178 | Holly | *Malus* × *domestica* | 126 |
| --- | --- | --- | --- |
| 179 | Jinyu | *Malus* × *domestica* | 134 |
| 180 | Miguo | *Malus* × *domestica* | 157 |
| 181 | Qinglong | *Malus* × *domestica* | 164 |
| 182 | Jacques Lebe-l | *Malus* × *domestica* | NA |
| 183 | GS48 | *Malus* × *domestica* | 126 |
| 184 | Hongxue | *Malus* × *domestica* | 143 |
| 185 | Stark spur | *Malus* × *domestica* | 143 |
| 186 | Zaohongxia | *Malus* × *domestica* | 165 |
| 187 | K10 | *Malus* × *domestica* | 134 |
| 188 | Beijing 0201 | *Malus* × *domestica* | 139 |
| 189 | Lijiangshandingzi | *Malus rockii* Rehder | 149 |
| 190 | Megumi | *Malus* × *domestica* | 157 |
| 191 | Judestar | *Malus* × *domestica* | 155 |
| 192 | Fuji_60-22-16 | *Malus* × *domestica* | 139 |
| 193 | Qingsenzaosheng | *Malus* × *domestica* | 116 |
| 194 | Benika | *Malus* × *domestica* | 77 |
| 195 | Judaine | *Malus* × *domestica* | 143 |
| 196 | Norsan | *Malus* × *domestica* | 136 |
| 197 | Red Baron | *Malus* × *domestica* | 76 |
| 198 | Hongfushi TAC | *Malus* × *domestica* | 136 |
| 199 | Xinjiangyepingguo 20-9 | *Malus sieversii* (Ledeb.) M.Roem. | 164 |
| 200 | Qingsen 3 | *Malus* × *domestica* | 136 |
| 201 | Red Reinette Du Canada | *Malus* × *domestica* | 151 |
| 203 | Tianhongyu | *Malus* × *domestica* | 126 |
| 204 | Ⅲ19-13 | *Malus* × *domestica* | 164 |
| 205 | Fujin | *Malus* × *domestica* | 121 |
| 206 | Yanhongmi | *Malus* × *domestica* | 165 |
| 207 | Xinjiang 15-9 | *Malus* × *domestica* subsp.chinesnsis. | 163 |
| 208 | Rome Beauty | *Malus* × *domestica* | 85 |
| 209 | Huichaoduan | *Malus* × *domestica* | NA |
| 210 | Qingguan | *Malus* × *domestica* | 164 |
| 211 | Calville Rouge | *Malus* × *domestica* | 136 |
| 212 | De 6 | *Malus* × *domestica* | 120 |
| 213 | Jieba | *Malus* × *domestica* | 120 |
| 214 | Xinhongyu | *Malus* × *domestica* | 163 |
| 215 | Hahong | *Malus* × *domestica* | 143 |
| 216 | Meiguihong | *Malus* × *domestica* | 143 |
| 217 | Hebeikangbingjinguan | *Malus* × *domestica* | 130 |
| 218 | Mianpingguo | *Malus* × *domestica* subsp.chinesnsis. | 151 |
| 219 | Zhumaliya | *Malus* × *domestica* | 143 |
| 220 | Lanpengwang | *Malus* × *domestica* | 143 |
| 221 | 60-15-30 | *Malus* × *domestica* | 115 |
| 222 | Xinjiang 30-0 | *Malus* × *domestica* subsp.chinesnsis. | 117 |
| 223 | Ingram | *Malus* × *domestica* | 164 |
| 224 | Qiujin | *Malus* × *domestica* | 164 |
| 225 | Shanglin | *Malus* × *domestica* | 165 |
| 226 | Koi Hime | *Malus* × *domestica* | 116 |
| 227 | Avrolles | *Malus* × *domestica* | 165 |
| 228 | Qingsenduanzhi Fuji | *Malus* × *domestica* | 165 |
| 229 | Opalescent | *Malus* × *domestica* | 143 |
| 230 | K9 | *Malus* × *domestica* | 85 |
| 231 | Onieffnin | *Malus* × *domestica* | 155 |
| 232 | Xiboliyabaidian | *Malus* × *domestica* | 88 |
| 234 | Longdonghaitang | *Malus kansuensis* (Batal.) Schneid. | 165 |
| 235 | Fuji_80-1-70-3 | *Malus* × *domestica* | 165 |
| 236 | Florina | *Malus* × *domestica* | 136 |
| 237 | Chicheng | *Malus* × *domestica* | 120 |
| 238 | Suyisiliebo | *Malus* × *domestica* | 99 |
| 240 | Weixisanye | *Malus sieboldii* (Regel) Rehder | 165 |
| 241 | Toko | *Malus* × *domestica* | 164 |
| 242 | Tianhuangkui | *Malus* × *domestica* | 98 |
| 243 | Xishuhaitang | *Malus prattii* (Hemsl.) C.K.Schneid. | 111 |
| 244 | Shengnong 2 | *Malus* × *domestica* | 116 |
| 245 | Dalu 52 | *Malus* × *domestica* subsp.chinesnsis. | 138 |
| 246 | Xingcheng 1-14 | *Malus* × *domestica* | 112 |
| 247 | Xijinhaitang | *sikkimensis* (Wenz.) Koehne ex C.K.S | 165 |
| 248 | Huangguniang | *Malus* × *domestica* | 85 |
| 249 | Huaguanhaitang | *Malus coronaria* (L.) Mill. | 171 |
| 250 | Zhaaishandingzi | *Malus baccata* (L.) Borkh. | 165 |
| 251 | Maoshanjingzi | *Malus mandshurica* (Maxim.) Kom | 136 |
| 252 | Hongsanye | *Malus sieboldii* (Regel) Rehder | 165 |
| 253 | Nai 2 | *Malus* × *domestica* subsp.chinesnsis. | NA |
| 254 | Kuihua | *Malus* × *domestica* | 164 |
| 255 | Gudeboge | *Malus* × *domestica* | 138 |
| 258 | Haitanghua | *Malus spectabilis* (Ait.) Borkh. | 165 |
| 259 | 60-4-4 | *Malus* × *domestica* | 103 |
| 263 | Jerseymac | *Malus* × *domestica* | 103 |
| 265 | Xingcheng 21-23 | *Malus* × *domestica* | 143 |
| 268 | Xingcheng 8-8 | *Malus* × *domestica* | 136 |
| 269 | Reinette | *Malus* × *domestica* | 103 |
| 271 | Meiduan 1 | *Malus* × *domestica* | 136 |

| 273 | Fa 5 | *Malus* × *domestica* | 163 |
| --- | --- | --- | --- |
| 275 | Apple of Commerce | *Malus* × *domestica* | 133 |
| 276 | Mantanghong | *Malus* × *domestica* | 136 |
| 277 | Kosttiq | *Malus* × *domestica* | 165 |
| 279 | Xingcheng 17-10 | *Malus* × *domestica* | 121 |
| 280 | Xingcheng 23-1 | *Malus* × *domestica* | 136 |
| 281 | Jie 9 | *Malus* × *domestica* | 163 |
| 284 | Early Ortley | *Malus* × *domestica* | 136 |
| 286 | Meixiang | *Malus* × *domestica* | 165 |
| 289 | Xingcheng 23-10 | *Malus* × *domestica* | 165 |
| 290 | Benoni | *Malus* × *domestica* | 136 |
| 291 | Qihuyihao 2 | *Malus* × *domestica* | 158 |
| 292 | De 14 | *Malus* × *domestica* | 136 |
| 298 | Early Harvest | *Malus* × *domestica* | 121 |
| 312 | Stark Spur Ultra Red Delicious 1 | *Malus* × *domestica* | 136 |
| 313 | Xingcheng 16-21 | *Malus* × *domestica* | 164 |
| 314 | Fuji_Ⅰ_11-2_ | *Malus* × *domestica* | 165 |
| 315 | Stark Spur Ultra Red Delicious 2 | *Malus* × *domestica* | 136 |
| 316 | Arkansas | *Malus* × *domestica* | 136 |
| 317 | Kogetsu | *Malus* × *domestica* | 136 |
| 318 | 1465 | *Malus* × *domestica* | 164 |
| 320 | Giant Jenifon | *Malus* × *domestica* | 139 |
| 321 | Jinhong | *Malus* × *domestica* | 112 |
| 322 | Jiguan | *Malus* × *domestica* | 164 |
| 323 | Hongao | *Malus* × *domestica* | 126 |
| 325 | Qianhetiguoguang | *Malus* × *domestica* | 164 |
| 327 | Xindong | *Malus* × *domestica* | 136 |
| 328 | Ⅱ10-15 | *Malus* × *domestica* | 136 |
| 329 | Xingcheng 10-3 | *Malus* × *domestica* | 151 |
| 330 | Hongge | *Malus* × *domestica* | 110 |
| 331 | Qunfu 1 | *Malus* × *domestica* | 164 |
| 332 | Qiulimeng | *Malus* × *domestica* | 136 |
| 333 | Zhaiteng 2 | *Malus* × *domestica* | 165 |
| 334 | Sakata Tsugaru | *Malus* × *domestica* | 130 |
| 335 | Ningguan | *Malus* × *domestica* | 165 |
| 336 | Alps Otome | *Malus* × *domestica* | 164 |
| 338 | Xinhua 2 | *Malus* × *domestica* | 136 |
| 339 | Yingqiu | *Malus* × *domestica* | 136 |
| 340 | Ningqiu | *Malus* × *domestica* | 121 |
| 341 | Guoguang_60-12-23 | *Malus* × *domestica* | 136 |
| 342 | Akifu 1 | *Malus* × *domestica* | 136 |
| 343 | Xingcheng 25-16 | *Malus* × *domestica* | 165 |
| 344 | Chenango Strawberry | *Malus* × *domestica* | 130 |
| 345 | Shengli | *Malus* × *domestica* | 151 |
| 346 | Spartan | *Malus* × *domestica* | 165 |
| 347 | Xishan 1 | *Malus* × *domestica* | 139 |
| 348 | Fa 3 | *Malus* × *domestica* | 127 |
| 349 | Ben Davis | *Malus* × *domestica* | 164 |
| 350 | Rt du Mans | *Malus* × *domestica* | 164 |
| 351 | Stonetosh | *Malus* × *domestica* | 151 |
| 352 | Changye 1 | *Malus* × *domestica* | 164 |
| 353 | N2 | *Malus* × *domestica* | 136 |
| 354 | Xiaguang | *Malus* × *domestica* | 85 |
| 355 | Xingcheng 19-1 | *Malus* × *domestica* | 151 |
| 357 | Jincui | *Malus* × *domestica* | 130 |
| 358 | Bianqiangzi 2 | *Malus* × *domestica* | 125 |
| 359 | Qiufu 6 | *Malus* × *domestica* | 143 |
| 360 | Pionier | *Malus* × *domestica* | 164 |
| 361 | Pinova | *Malus* × *domestica* | 141 |
| 362 | Wuxiujinguan | *Malus* × *domestica* | 133 |
| 363 | Pacific Rose | *Malus* × *domestica* | 165 |
| 365 | Qiufu 7 | *Malus* × *domestica* | 134 |
| 366 | Rizhiwan-1 | *Malus* × *domestica* | 164 |
| 367 | Cuiyu | *Malus* × *domestica* | 164 |
| 368 | Jie 15 | *Malus* × *domestica* | 136 |
| 369 | Jinhong | *Malus* × *domestica* | 136 |
| 370 | Zaocuilv | *Malus* × *domestica* | 155 |
| 371 | Antalue | *Malus* × *domestica* | 136 |
| 372 | Dailv | *Malus* × *domestica* | 116 |
| 373 | Kufeigan | *Malus* × *domestica* | 165 |
| 374 | Melton | *Malus* × *domestica* | 143 |
| 375 | Magu | *Malus* × *domestica* | 136 |
| 376 | Debaohantang | *Malus prunifolia* (Willd.) Borkh. | 169 |
| 377 | Yellow transparent | *Malus* × *domestica* | 81 |
| 378 | Qiufu 39 | *Malus* × *domestica* | 165 |
| 379 | Donghongguo | *Malus spectabilis* (Ait.) Borkh. | 153 |
| 380 | GuoguangⅡ_8-19_ | *Malus* × *domestica* | 164 |
| 381 | Liaofu | *Malus* × *domestica* | 85 |
| 382 | Xingcheng 3-2 | *Malus* × *domestica* | 116 |
| 383 | Pingpoguo | *Malus* × *domestica* subsp.chinesnsis. | 165 |
| 385 | Heiyushandingzi | *Malus baccata* (L.) Borkh. | 167 |
| 388 | Mosiketouming | *Malus* × *domestica* | 126 |

| 389 | Bianyehaitang | *Malus toringoides* (Rehd.) Hughes | 165 |
| --- | --- | --- | --- |
| 390 | Ruby | *Malus* × *domestica* | 163 |
| 391 | Linzhihaitang | *Malus sikkimensis* (Wenzig.)Koehne | 166 |
| 392 | Zaotaideman | *Malus* × *domestica* | 103 |
| 393 | Lowtosh | *Malus* × *domestica* | 116 |
| 394 | Daihong | *Malus* × *domestica* | 143 |
| 395 | Liberty | *Malus* × *domestica* | 143 |
| 396 | Qingxiang | *Malus* × *domestica* | 155 |
| 397 | Jiabukajinguan | *Malus* × *domestica* | 133 |
| 398 | Zhongxing | *Malus* × *domestica* | 116 |
| 399 | Jacques Lebel-2 | *Malus* × *domestica* | 121 |
| 400 | Ningmenghaitang | *Malus prunifolia* (Willd.) Borkh. | 177 |
| 401 | Xingcheng 3-19 | *Malus* × *domestica* | 121 |
| 402 | Xingcheng 8-12 | *Malus* × *domestica* | 136 |
| 403 | Sichuanbianye | *Malus toringoides* (Rehd.) Hughes | 136 |
| 404 | Huadao | *Malus* × *domestica* | 112 |
| 405 | Beauty of Bath | *Malus* × *domestica* | 79 |
| 406 | Summerland | *Malus* × *domestica* | 139 |
| 407 | Ruila | *Malus* × *domestica* | 165 |
| 408 | Shalatuoni | *Malus* × *domestica* | 138 |
| 409 | Youyimeigui | *Malus* × *domestica* | 85 |
| 410 | Xifuhaitang | *Malus micromalus* Makino | 165 |
| 411 | Macoun | *Malus* × *domestica* | 165 |
| 412 | Deqinhantang | *Malus Sargentii* Rehd | 168 |
| 414 | Fushuai | *Malus* × *domestica* | 139 |
| 416 | Stark Earlibaze | *Malus* × *domestica* | 116 |
| 417 | Shajinhaitang | *Malus Sargentii* Rehd | 165 |
| 418 | Hesefengli | *Malus* × *domestica* | 93 |
| 419 | Xiaojinbianye | *Malus toringoides* (Rehd.) Hughes | 165 |
| 420 | Mengpaisi | *Malus* × *domestica* | 173 |
| 421 | Wushanbianye | *Malus toringoides* (Rehd.) Hughes | 165 |
| 422 | Yajiangbianyehaitang | *Malus toringoides* (Rehd.) Hughes | 165 |
| 423 | Binzi | *Malus asiatica* var.rinki (Koidz.)Asami. | 132 |
| 424 | Zhaojueshandingzi | *Malus baccata* (L.) Borkh. | 175 |
| 425 | Duohuahaitang | *Malus floribunda* Siebold ex Van Houtt | 165 |
| 426 | Smith Cider | *Malus* × *domestica* | 150 |
| 427 | Female Guerrillas | *Malus* × *domestica* | 119 |
| 431 | Houjiadianduanhongxin | *Malus* × *domestica* | 139 |
| 432 | Xingcheng 12-11 | *Malus* × *domestica* | 136 |
| 435 | Freyberg | *Malus* × *domestica* | 163 |
| 438 | Ralls Genet-1 | *Malus* × *domestica* | 163 |
| 442 | Ganhongyu | *Malus* × *domestica* | 121 |
| 444 | Jinguan2 | *Malus* × *domestica* | 144 |
| 445 | Xinjiangyepingguo 5 | *Malus sieversii* (Ledeb.) M.Roem. | NA |
| 448 | 60-17-17 | *Malus* × *domestica* | 121 |
| 458 | 600T | *Malus* × *domestica* | 136 |
| 459 | Budai Domokos | *Malus* × *domestica* | 163 |
| 483 | Drumbo | *Malus* × *domestica* | 164 |
| 498 | Husveti Rosmaring | *Malus* × *domestica* | 164 |
| 499 | Qiufu 1 | *Malus* × *domestica* | 164 |
| 500 | Fengyan | *Malus* × *domestica* | NA |
| 501 | Ganhongyu | *Malus* × *domestica* | 136 |
| 502 | Shengfangfu 3a | *Malus* × *domestica* | 165 |
| 503 | Shengfangfu 1 | *Malus* × *domestica* | 136 |
| 504 | Beifangxinabo | *Malus* × *domestica* | 136 |
| 505 | Cox's Orange Pippin | *Malus* × *domestica* | 136 |
| 506 | Eraly red | *Malus* × *domestica* | 128 |
| 507 | Shizishan 2 | *Malus* × *domestica* | 136 |
| 508 | Hongguoguang | *Malus* × *domestica* | 136 |
| 509 | Sky spur | *Malus* × *domestica* | 164 |
| 510 | 52-6-7 | *Malus* × *domestica* | 141 |
| 511 | Himekami | *Malus* × *domestica* | 143 |
| 512 | Ningguang | *Malus* × *domestica* | 126 |
| 513 | Youyi | *Malus* × *domestica* | 143 |
| 514 | Bo 7 | *Malus* × *domestica* | 136 |
| 515 | Cuihong | *Malus* × *domestica* | NA |
| 516 | Qiufu 5 | *Malus* × *domestica* | 126 |
| 517 | Boiken | *Malus* × *domestica* | 164 |
| 518 | Ruby Red | *Malus* × *domestica* | 165 |
| 519 | Bensuotehao | *Malus* × *domestica* | 164 |
| 521 | Early McIntosh | *Malus* × *domestica* | 91 |
| 522 | Sunggold | *Malus* × *domestica* | 157 |
| 523 | Batul-Alma | *Malus* × *domestica* | 165 |
| 524 | Tsugaru | *Malus* × *domestica* | 120 |
| 525 | Hongxijinqing | *Malus* × *domestica* | 122 |
| 526 | Shizishan | *Malus* × *domestica* | 121 |
| 527 | Melba | *Malus* × *domestica* | 112 |
| 528 | Huashuai 1 | *Malus* × *domestica* | 138 |
| 529 | Hanfu | *Malus* × *domestica* | 165 |
| 530 | Black Gilliflower | *Malus* × *domestica* | 121 |
| 531 | Zhongqiu | *Malus* × *domestica* | 136 |
| 532 | Ⅰ8-5 | *Malus* × *domestica* | 138 |

| 533 | Pingzhiguoguang | *Malus* × *domestica* | 163 |
| --- | --- | --- | --- |
| 534 | Jinguang | *Malus* × *domestica* | 120 |
| 535 | Enweierjinaisheng | *Malus* × *domestica* | 138 |
| 536 | Dongtian | *Malus* × *domestica* | 112 |
| 537 | Sipadun | *Malus* × *domestica* | 139 |
| 538 | Black Ben Davis | *Malus* × *domestica* | 136 |
| 539 | Honey Gold | *Malus* × *domestica* | 120 |
| 540 | Babusijinuo | *Malus* × *domestica* | NA |
| 541 | Red Golden Gala | *Malus* × *domestica* | 143 |
| 542 | Zhanxuan 14 | *Malus* × *domestica* | 164 |
| 543 | Xiushuiguoguang | *Malus* × *domestica* | 130 |
| 544 | Beda | *Malus* × *domestica* | 165 |
| 545 | Rizhiwan-2 | *Malus* × *domestica* | 157 |
| 546 | Scarlet Spur | *Malus* × *domestica* | 116 |
| 548 | Xingcheng 3-20 | *Malus* × *domestica* | 122 |
| 549 | Sanyehaitang | *Malus sieboldii* (Regel) Rehder | 166 |
| 550 | Michurina | *Malus* × *domestica* | 120 |
| 552 | Sharp Red | *Malus* × *domestica* | 133 |
| 554 | Evelyn | *Malus* × *domestica* | 113 |
| 559 | Jinguan 16-20 | *Malus* × *domestica* | 163 |
| 560 | Qiufu 39 | *Malus* × *domestica* | 163 |
| 562 | Kelia | *Malus* × *domestica* | 116 |
| 563 | Norland | *Malus* × *domestica* | 120 |
| 564 | Changhong 3 | *Malus* × *domestica* | 151 |
| 565 | Chuizhiguoguang | *Malus* × *domestica* | 165 |
| 566 | Sekaiichi | *Malus* × *domestica* | 164 |
| 567 | Chiefeain | *Malus* × *domestica* | 126 |
| 569 | Xingcheng 18-10 | *Malus* × *domestica* | 136 |
| 570 | Starkjambo | *Malus* × *domestica* | 133 |
| 571 | I12-10 | *Malus* × *domestica* | 78 |
| 572 | xinguoguang | *Malus* × *domestica* | 165 |
| 573 | 60-1-59 | *Malus* × *domestica* | 120 |
| 574 | Bo 5 | *Malus* × *domestica* | 163 |
| 575 | De 8 | *Malus* × *domestica* | 165 |
| 576 | Royal Red | *Malus* × *domestica* | 133 |
| 577 | Nagafu 7 | *Malus* × *domestica* | 164 |
| 578 | Judeline | *Malus* × *domestica* | 133 |
| 579 | Xinlimei | *Malus* × *domestica* | 151 |
| 580 | Calville Blanche | *Malus* × *domestica* | 144 |
| 581 | Meltosh | *Malus* × *domestica* | 164 |
| 582 | Xingcheng 18-18 | *Malus* × *domestica* | 110 |
| 601 | Mianpingguo 2 | *Malus* × *domestica* subsp.chinesnsis. | NA |
| 602 | Xinping 1 | *Malus* × *domestica* | 156 |
| 603 | Kuiping 2 | *Malus* × *domestica* | 157 |
| 604 | Xinjiang 4-10 | *Malus* × *domestica* subsp.chinesnsis. | NA |
| 605 | Huahong | *Malus asiatica* Nakai | NA |
| 606 | Nai | *Malus* × *domestica* subsp.chinesnsis. | NA |
| 607 | Oregon Spur 9 | *Malus* × *domestica* | NA |
| 608 | Xingcheng 0-9 | *Malus* × *domestica* | NA |
| 609 | Xinping 4 | *Malus* × *domestica* | NA |
| 610 | Zhumeihaitang | *Malus zumi* (Mats.) Rehder | 136 |
| 620 | Winter Banana | *Malus* × *domestica* | 117 |
| 621 | Geneva Early | *Malus* × *domestica* | 95 |
| 622 | Meiguo 8 | *Malus* × *domestica* | 143 |
| 623 | Maigold | *Malus* × *domestica* | 130 |
| 624 | Daguoshandingzi | *Malus baccata* (L.) Borkh. | 169 |

| **Supplementary Table 2. List of the significantly associated SNPs and genes in chromosome 03 for maturity date in apple fruits.** | | | | | | | |
| --- | --- | --- | --- | --- | --- | --- | --- |
| **ID** | **Chr** | **Ref** | **Alt** | **P** | **-log10P** | **Location** | **Gene** |
| Chr03_27379185 | Chr03 | A | G | 6.93022E-08 | 7.159253 | intronic | gene:MD03G1200000 |
| Chr03_27379811 | Chr03 | C | T | 5.07707E-08 | 7.294387 | intronic | gene:MD03G1200000 |
| Chr03_27379886 | Chr03 | T | C | 5.07707E-08 | 7.294387 | intronic | gene:MD03G1200000 |
| Chr03_27391177 | Chr03 | G | A | 2.50667E-09 | 8.600903 | intronic | gene:MD03G1200200 |
| Chr03_27585651 | Chr03 | C | T | 1.43577E-08 | 7.842915 | intergenic | ene:MD03G1201500(dist=10792),gene:MD03G1201600(dist=5586) |
| Chr03_27657509 | Chr03 | T | C | 2.59797E-08 | 7.585366 | intronic | gene:MD03G1202300 |
| Chr03_27666010 | Chr03 | G | A | 2.15212E-08 | 7.667133 | intronic | gene:MD03G1202300 |
| Chr03_27666051 | Chr03 | C | T | 2.15212E-08 | 7.667133 | intronic | gene:MD03G1202300 |
| Chr03_27672287 | Chr03 | A | T | 5.09889E-08 | 7.292524 | intronic | gene:MD03G1202300 |
| Chr03_27672994 | Chr03 | T | C | 1.30246E-08 | 7.885235 | intronic | gene:MD03G1202300 |
| Chr03_27673008 | Chr03 | G | A | 1.30246E-08 | 7.885235 | intronic | gene:MD03G1202300 |
| Chr03_27694229 | Chr03 | C | A | 4.44887E-10 | 9.35175 | intronic | gene:MD03G1202700 |
| Chr03_28193825 | Chr03 | C | T | 1.91682E-09 | 8.717419 | intergenic | gene:MD03G1206200(dist=6608),gene:MD03G1206300(dist=1722) |
| Chr03_28220737 | Chr03 | G | A | 1.5817E-08 | 7.800877 | intergenic | ene:MD03G1206300(dist=22196),gene:MD03G1206400(dist=22400) |
| Chr03_28224365 | Chr03 | A | G | 1.8042E-08 | 7.743717 | intergenic | ene:MD03G1206300(dist=25824),gene:MD03G1206400(dist=18772) |
| Chr03_28253643 | Chr03 | A | T | 1.5023E-09 | 8.823243 | downstream | gene:MD03G1206500(dist=414) |
| Chr03_28352468 | Chr03 | C | T | 4.35567E-08 | 7.360945 | intergenic | ene:MD03G1207200(dist=26472),gene:MD03G1207300(dist=4681) |
| Chr03_28370288 | Chr03 | G | A | 1.98244E-09 | 8.7028 | downstream | gene:MD03G1207400(dist=940) |
| Chr03_28703760 | Chr03 | G | A | 5.39536E-09 | 8.26798 | intergenic | ene:MD03G1207900(dist=250160),gene:MD03G1208000(dist=6057) |
| Chr03_28765008 | Chr03 | G | T | 2.43324E-08 | 7.613816 | intergenic | ene:MD03G1208500(dist=2707),gene:MD03G1208600(dist=11976) |
| Chr03_28765158 | Chr03 | C | T | 1.79992E-09 | 8.744747 | intergenic | ene:MD03G1208500(dist=2857),gene:MD03G1208600(dist=11826) |
| Chr03_28771287 | Chr03 | C | T | 3.4997E-10 | 9.455969 | intergenic | gene:MD03G1208500(dist=8986),gene:MD03G1208600(dist=5697) |
| Chr03_28771297 | Chr03 | G | T | 3.4997E-10 | 9.455969 | intergenic | gene:MD03G1208500(dist=8996),gene:MD03G1208600(dist=5687) |
| Chr03_28771303 | Chr03 | G | A | 2.28042E-08 | 7.641986 | intergenic | gene:MD03G1208500(dist=9002),gene:MD03G1208600(dist=5681) |
| Chr03_28775556 | Chr03 | G | A | 2.56946E-08 | 7.590158 | intergenic | ene:MD03G1208500(dist=13255),gene:MD03G1208600(dist=1428) |
| Chr03_28779587 | Chr03 | C | T | 4.5681E-10 | 9.340265 | upstream | gene:MD03G1208600(dist=805) |
| Chr03_28884002 | Chr03 | T | A | 2.03628E-09 | 8.691162 | intronic | gene:MD03G1209500 |
| Chr03_28884241 | Chr03 | C | T | 2.03628E-09 | 8.691162 | intronic | gene:MD03G1209500 |
| Chr03_28943404 | Chr03 | A | C | 2.211E-08 | 7.655411 | intronic | gene:MD03G1210300 |
| Chr03_28965495 | Chr03 | C | T | 1.00144E-08 | 7.999375 | intergenic | gene:MD03G1210500(dist=7545),gene:MD03G1210600(dist=5268) |
| Chr03_28972372 | Chr03 | C | T | 5.85008E-09 | 8.232838 | intergenic | gene:MD03G1210600(dist=1536),gene:MD03G1210700(dist=1845) |
| Chr03_29047334 | Chr03 | A | G | 4.39924E-08 | 7.356622 | downstream | gene:MD03G1211300(dist=535) |
| Chr03_29179211 | Chr03 | C | T | 5.64557E-08 | 7.248292 | intronic | gene:MD03G1212600 |
| Chr03_29190333 | Chr03 | T | C | 5.13333E-08 | 7.289601 | intergenic | ene:MD03G1212600(dist=10646),gene:MD03G1212700(dist=4427) |
| Chr03_29190391 | Chr03 | A | G | 4.04775E-09 | 8.392786 | intergenic | ene:MD03G1212600(dist=10704),gene:MD03G1212700(dist=4369) |
| Chr03_29193593 | Chr03 | T | C | 3.54896E-08 | 7.449899 | intergenic | ene:MD03G1212600(dist=13906),gene:MD03G1212700(dist=1167) |
| Chr03_29193758 | Chr03 | A | C | 5.29231E-08 | 7.276355 | intergenic | ene:MD03G1212600(dist=14071),gene:MD03G1212700(dist=1002) |
| Chr03_29194914 | Chr03 | C | A | 1.03677E-08 | 7.984319 | UTR3 | gene:MD03G1212700(mRNA:MD03G1212700:c.*189G>T) |
| Chr03_29198653 | Chr03 | A | G | 1.3304E-08 | 7.876018 | upstream | gene:MD03G1212700(dist=271) |
| Chr03_29202449 | Chr03 | G | A | 3.71297E-11 | 10.43028 | nonsynonymous SNV | e:MD03G1212900:mRNA:MD03G1212900:exon16:c.C2159T:p.S720F, |
| Chr03_29210023 | Chr03 | T | A | 1.94421E-09 | 8.711258 | intergenic | ene:MD03G1212900(dist=2745),gene:MD03G1213000(dist=10865) |
| Chr03_29213602 | Chr03 | C | A | 1.39246E-08 | 7.856218 | intergenic | gene:MD03G1212900(dist=6324),gene:MD03G1213000(dist=7286) |
| Chr03_29214779 | Chr03 | A | C | 1.04455E-08 | 7.98107 | intergenic | gene:MD03G1212900(dist=7501),gene:MD03G1213000(dist=6109) |
| Chr03_29220721 | Chr03 | G | A | 2.25499E-08 | 7.646856 | upstream | gene:MD03G1213000(dist=167) |
| Chr03_29225882 | Chr03 | C | T | 1.99537E-08 | 7.699978 | intronic | gene:MD03G1213000 |
| Chr03_29226537 | Chr03 | C | A | 3.29945E-09 | 8.481559 | intronic | gene:MD03G1213000 |
| Chr03_29239752 | Chr03 | C | T | 1.59159E-08 | 7.798168 | intergenic | ene:MD03G1213000(dist=4014),gene:MD03G1213100(dist=17149) |
| Chr03_29264044 | Chr03 | C | T | 2.79679E-09 | 8.553341 | intergenic | ene:MD03G1213100(dist=5710),gene:MD03G1213200(dist=14271) |
| Chr03_29280461 | Chr03 | G | A | 2.49674E-08 | 7.602626 | UTR5 | gene:MD03G1213300(mRNA:MD03G1213300:c.-75C>T) |
| Chr03_29317150 | Chr03 | A | G | 1.26097E-08 | 7.899294 | intronic | gene:MD03G1213800 |
| Chr03_29389274 | Chr03 | A | C | 5.93819E-08 | 7.226346 | upstream;downstream | gene:MD03G1214200(dist=337);gene:MD03G1214300(dist=781) |
| Chr03_29389275 | Chr03 | C | T | 5.93819E-08 | 7.226346 | upstream;downstream | gene:MD03G1214200(dist=338);gene:MD03G1214300(dist=780) |
| Chr03_29389513 | Chr03 | A | G | 4.4744E-09 | 8.349265 | upstream;downstream | gene:MD03G1214200(dist=576);gene:MD03G1214300(dist=542) |
| Chr03_29389524 | Chr03 | A | T | 4.4744E-09 | 8.349265 | upstream;downstream | gene:MD03G1214200(dist=587);gene:MD03G1214300(dist=531) |
| Chr03_29567042 | Chr03 | C | T | 1.5053E-09 | 8.822378 | intergenic | gene:MD03G1215200(dist=5004),gene:MD03G1215300(dist=3405) |
| Chr03_30127376 | Chr03 | G | C | 3.04818E-08 | 7.51596 | intergenic | ene:MD03G1217100(dist=2165),gene:MD03G1217200(dist=13002) |
| Chr03_30127699 | Chr03 | C | G | 9.84699E-10 | 9.006697 | intergenic | ene:MD03G1217100(dist=2488),gene:MD03G1217200(dist=12679) |
| Chr03_30164630 | Chr03 | G | A | 5.96157E-08 | 7.22464 | intergenic | ene:MD03G1217300(dist=9957),gene:MD03G1217400(dist=17210) |
| Chr03_30164653 | Chr03 | A | T | 5.96157E-08 | 7.22464 | intergenic | ene:MD03G1217300(dist=9980),gene:MD03G1217400(dist=17187) |
| Chr03_30164667 | Chr03 | A | T | 5.96157E-08 | 7.22464 | intergenic | ene:MD03G1217300(dist=9994),gene:MD03G1217400(dist=17173) |
| Chr03_30164722 | Chr03 | A | G | 1.5644E-08 | 7.805652 | intergenic | ene:MD03G1217300(dist=10049),gene:MD03G1217400(dist=17118) |
| Chr03_30181724 | Chr03 | A | G | 1.13891E-08 | 7.94351 | upstream | gene:MD03G1217400(dist=116) |
| Chr03_30263235 | Chr03 | C | T | 5.73423E-08 | 7.241525 | intronic | gene:MD03G1218200 |
| Chr03_30263477 | Chr03 | G | T | 4.46171E-08 | 7.350498 | nonsynonymous SNV | ene:MD03G1218200:mRNA:MD03G1218200:exon1:c.C71A:p.A24D, |
| Chr03_30318094 | Chr03 | T | C | 2.78623E-08 | 7.554983 | synonymous SNV | ne:MD03G1218400:mRNA:MD03G1218400:exon3:c.A468G:p.E156E, |
| Chr03_30321568 | Chr03 | C | A | 6.84069E-08 | 7.1649 | intergenic | gene:MD03G1218400(dist=2711),gene:MD03G1218500(dist=9223) |
| Chr03_30332875 | Chr03 | G | C | 4.87032E-08 | 7.312443 | ncRNA_intronic | gene:MD03G1218700 |
| Chr03_30334377 | Chr03 | A | G | 6.03767E-09 | 8.219131 | upstream;downstream | gene:MD03G1218800(dist=181);gene:MD03G1218700(dist=781) |
| Chr03_30359360 | Chr03 | G | A | 2.98868E-08 | 7.52452 | intergenic | ene:MD03G1219000(dist=7465),gene:MD03G1219100(dist=14549) |
| Chr03_30363096 | Chr03 | G | A | 5.46163E-08 | 7.262678 | intergenic | ene:MD03G1219000(dist=11201),gene:MD03G1219100(dist=10813) |
| Chr03_30365699 | Chr03 | T | C | 6.92606E-08 | 7.159513 | intergenic | ene:MD03G1219000(dist=13804),gene:MD03G1219100(dist=8210) |
| Chr03_30366294 | Chr03 | G | A | 5.87827E-08 | 7.23075 | intergenic | ene:MD03G1219000(dist=14399),gene:MD03G1219100(dist=7615) |
| Chr03_30411863 | Chr03 | T | A | 3.26349E-08 | 7.486317 | upstream | gene:MD03G1219400(dist=136) |
| Chr03_30415578 | Chr03 | T | G | 3.98487E-08 | 7.399585 | UTR5 | gene:MD03G1219500(mRNA:MD03G1219500:c.-172T>G) |
| Chr03_30453464 | Chr03 | G | A | 4.27571E-10 | 9.368992 | upstream | gene:MD03G1220300(dist=872) |
| Chr03_30476016 | Chr03 | C | T | 3.28935E-08 | 7.48289 | downstream | gene:MD03G1220800(dist=468) |
| Chr03_30570409 | Chr03 | G | A | 1.80931E-08 | 7.742487 | downstream | gene:MD03G1221700(dist=920) |
| Chr03_30572967 | Chr03 | C | T | 1.04475E-10 | 9.980986 | intergenic | gene:MD03G1221700(dist=3478),gene:MD03G1221800(dist=5159) |
| Chr03_30582696 | Chr03 | T | G | 3.04446E-08 | 7.51649 | intergenic | gene:MD03G1221800(dist=3653),gene:MD03G1221900(dist=3139) |
| Chr03_30584647 | Chr03 | C | A | 2.88215E-09 | 8.540284 | intergenic | gene:MD03G1221800(dist=5604),gene:MD03G1221900(dist=1188) |
| Chr03_30584879 | Chr03 | C | T | 3.94231E-08 | 7.40425 | upstream | gene:MD03G1221900(dist=956) |

| Chr03_30584901 | Chr03 | T | G | 1.14181E-08 | 7.942406 | upstream | gene:MD03G1221900(dist=934) |
| --- | --- | --- | --- | --- | --- | --- | --- |
| Chr03_30585300 | Chr03 | A | T | 5.88094E-09 | 8.230553 | upstream | gene:MD03G1221900(dist=535) |
| Chr03_30585461 | Chr03 | C | A | 3.16822E-08 | 7.499184 | upstream | gene:MD03G1221900(dist=374) |
| Chr03_30586633 | Chr03 | A | G | 4.50775E-08 | 7.34604 | downstream | gene:MD03G1221900(dist=724) |
| Chr03_30586814 | Chr03 | T | C | 1.10045E-08 | 7.958429 | downstream | gene:MD03G1221900(dist=905) |
| Chr03_30587009 | Chr03 | T | A | 2.77919E-10 | 9.556082 | intergenic | gene:MD03G1221900(dist=1100),gene:MD03G1222000(dist=2795) |
| Chr03_30588181 | Chr03 | G | A | 1.37345E-09 | 8.862188 | intergenic | gene:MD03G1221900(dist=2272),gene:MD03G1222000(dist=1623) |
| Chr03_30588461 | Chr03 | T | C | 1.16279E-09 | 8.934499 | intergenic | gene:MD03G1221900(dist=2552),gene:MD03G1222000(dist=1343) |
| Chr03_30588470 | Chr03 | T | C | 6.11123E-10 | 9.213872 | intergenic | gene:MD03G1221900(dist=2561),gene:MD03G1222000(dist=1334) |
| Chr03_30588472 | Chr03 | T | C | 6.11123E-10 | 9.213872 | intergenic | gene:MD03G1221900(dist=2563),gene:MD03G1222000(dist=1332) |
| Chr03_30588496 | Chr03 | C | T | 6.11123E-10 | 9.213872 | intergenic | gene:MD03G1221900(dist=2587),gene:MD03G1222000(dist=1308) |
| Chr03_30588503 | Chr03 | C | A | 4.26204E-09 | 8.370382 | intergenic | gene:MD03G1221900(dist=2594),gene:MD03G1222000(dist=1301) |
| Chr03_30588504 | Chr03 | G | A | 5.94025E-10 | 9.226196 | intergenic | gene:MD03G1221900(dist=2595),gene:MD03G1222000(dist=1300) |
| Chr03_30588508 | Chr03 | C | T | 5.94025E-10 | 9.226196 | intergenic | gene:MD03G1221900(dist=2599),gene:MD03G1222000(dist=1296) |
| Chr03_30588523 | Chr03 | A | G | 5.94025E-10 | 9.226196 | intergenic | gene:MD03G1221900(dist=2614),gene:MD03G1222000(dist=1281) |
| Chr03_30588736 | Chr03 | T | G | 8.34787E-10 | 9.078425 | intergenic | gene:MD03G1221900(dist=2827),gene:MD03G1222000(dist=1068) |
| Chr03_30588756 | Chr03 | A | G | 3.96955E-10 | 9.401259 | intergenic | gene:MD03G1221900(dist=2847),gene:MD03G1222000(dist=1048) |
| Chr03_30588834 | Chr03 | T | C | 4.46354E-09 | 8.35032 | downstream | gene:MD03G1222000(dist=970) |
| Chr03_30589691 | Chr03 | A | G | 1.08178E-10 | 9.965863 | downstream | gene:MD03G1222000(dist=113) |
| Chr03_30589789 | Chr03 | C | T | 5.8328E-11 | 10.23412 | downstream | gene:MD03G1222000(dist=15) |
| Chr03_30590223 | Chr03 | G | T | 2.34947E-09 | 8.629029 nonsynonymous SNVne:MD03G1222000:mRNA:MD03G1222000:exon7:c.C289A:p.H97N, | | |
| Chr03_30590314 | Chr03 | C | T | 1.01588E-08 | 7.993158 nonsynonymous SNVene:MD03G1222000:mRNA:MD03G1222000:exon7:c.G198A:p.M66I, | | |
| Chr03_30590315 | Chr03 | A | G | 1.01588E-08 | 7.993158 nonsynonymous SNVne:MD03G1222000:mRNA:MD03G1222000:exon7:c.T197C:p.M66T, | | |
| Chr03_30590370 | Chr03 | C | T | 7.03748E-09 | 8.152583 nonsynonymous SNVne:MD03G1222000:mRNA:MD03G1222000:exon7:c.G142A:p.D48N, | | |
| Chr03_30590904 | Chr03 | T | G | 1.61418E-09 | 8.792048 | UTR5 | gene:MD03G1222000(mRNA:MD03G1222000:c.-393A>C) |
| Chr03_30591059 | Chr03 | T | C | 7.37407E-10 | 9.132293 | intronic | gene:MD03G1222000 |
| Chr03_30615309 | Chr03 | C | T | 7.17605E-08 | 7.144115 | intergenic | ene:MD03G1222000(dist=22837),gene:MD03G1222100(dist=7905) |
| Chr03_30628309 | Chr03 | A | C | 1.05113E-08 | 7.978344 | intergenic | ene:MD03G1222100(dist=4241),gene:MD03G1222200(dist=10540) |
| Chr03_30632278 | Chr03 | C | T | 2.70215E-08 | 7.568291 | intergenic | gene:MD03G1222100(dist=8210),gene:MD03G1222200(dist=6571) |
| Chr03_30636930 | Chr03 | A | G | 9.86794E-09 | 8.005773 | intergenic | ene:MD03G1222100(dist=12862),gene:MD03G1222200(dist=1919) |
| Chr03_30637050 | Chr03 | A | T | 5.09655E-08 | 7.292724 | intergenic | ene:MD03G1222100(dist=12982),gene:MD03G1222200(dist=1799) |
| Chr03_30637052 | Chr03 | C | T | 5.09655E-08 | 7.292724 | intergenic | ene:MD03G1222100(dist=12984),gene:MD03G1222200(dist=1797) |
| Chr03_30637501 | Chr03 | G | A | 1.2351E-09 | 8.908298 | intergenic | ene:MD03G1222100(dist=13433),gene:MD03G1222200(dist=1348) |
| Chr03_30637647 | Chr03 | G | T | 2.3657E-10 | 9.62604 | intergenic | ene:MD03G1222100(dist=13579),gene:MD03G1222200(dist=1202) |
| Chr03_30637661 | Chr03 | C | T | 2.08093E-10 | 9.681741 | intergenic | ene:MD03G1222100(dist=13593),gene:MD03G1222200(dist=1188) |
| Chr03_30637685 | Chr03 | C | T | 2.11963E-10 | 9.673741 | intergenic | ene:MD03G1222100(dist=13617),gene:MD03G1222200(dist=1164) |
| Chr03_30637699 | Chr03 | C | T | 3.10747E-09 | 8.507592 | intergenic | ene:MD03G1222100(dist=13631),gene:MD03G1222200(dist=1150) |
| Chr03_30659301 | Chr03 | G | A | 1.01124E-08 | 7.995145 | intronic | gene:MD03G1222300 |
| Chr03_30661462 | Chr03 | G | A | 5.18449E-08 | 7.285294 | downstream | gene:MD03G1222300(dist=607) |
| Chr03_30675389 | Chr03 | C | T | 1.09331E-08 | 7.961257 | intergenic | gene:MD03G1222400(dist=1631),gene:MD03G1222500(dist=1569) |
| Chr03_30675960 | Chr03 | T | C | 1.61779E-08 | 7.791078 | downstream | gene:MD03G1222500(dist=998) |
| Chr03_30676012 | Chr03 | C | T | 7.2039E-09 | 8.142432 | downstream | gene:MD03G1222500(dist=946) |
| Chr03_30676317 | Chr03 | C | T | 5.11108E-08 | 7.291487 | downstream | gene:MD03G1222500(dist=641) |
| Chr03_30676371 | Chr03 | G | A | 1.46722E-08 | 7.833505 | downstream | gene:MD03G1222500(dist=587) |
| Chr03_30676411 | Chr03 | C | G | 3.67301E-10 | 9.434978 | downstream | gene:MD03G1222500(dist=547) |
| Chr03_30676947 | Chr03 | G | A | 3.80949E-09 | 8.419133 | downstream | gene:MD03G1222500(dist=11) |
| Chr03_30677144 | Chr03 | G | A | 6.76092E-10 | 9.169994 | synonymous SNV | ne:MD03G1222500:mRNA:MD03G1222500:exon1:c.C147T:p.P49P, |
| Chr03_30677337 | Chr03 | T | C | 1.67068E-09 | 8.777106 | upstream | gene:MD03G1222500(dist=47) |
| Chr03_30677855 | Chr03 | A | G | 6.11911E-09 | 8.213311 | upstream | gene:MD03G1222500(dist=565) |
| Chr03_30678091 | Chr03 | C | T | 4.47029E-10 | 9.349664 | upstream | gene:MD03G1222500(dist=801) |
| Chr03_30678176 | Chr03 | G | A | 2.69576E-09 | 8.569319 | upstream | gene:MD03G1222500(dist=886) |
| Chr03_30678471 | Chr03 | A | T | 3.20177E-08 | 7.49461 | intergenic | ene:MD03G1222500(dist=1181),gene:MD03G1222600(dist=17720) |
| Chr03_30678957 | Chr03 | C | T | 4.59699E-11 | 10.33753 | intergenic | ene:MD03G1222500(dist=1667),gene:MD03G1222600(dist=17234) |
| Chr03_30679570 | Chr03 | C | T | 3.28851E-10 | 9.483001 | intergenic | ene:MD03G1222500(dist=2280),gene:MD03G1222600(dist=16621) |
| Chr03_30679717 | Chr03 | C | G | 1.17453E-10 | 9.930137 | intergenic | ene:MD03G1222500(dist=2427),gene:MD03G1222600(dist=16474) |
| Chr03_30679719 | Chr03 | T | C | 1.17453E-10 | 9.930137 | intergenic | ene:MD03G1222500(dist=2429),gene:MD03G1222600(dist=16472) |
| Chr03_30679741 | Chr03 | A | G | 5.13989E-09 | 8.289046 | intergenic | ene:MD03G1222500(dist=2451),gene:MD03G1222600(dist=16450) |
| Chr03_30679783 | Chr03 | A | T | 5.13989E-09 | 8.289046 | intergenic | ene:MD03G1222500(dist=2493),gene:MD03G1222600(dist=16408) |
| Chr03_30679860 | Chr03 | G | C | 1.71465E-08 | 7.765825 | intergenic | ene:MD03G1222500(dist=2570),gene:MD03G1222600(dist=16331) |
| Chr03_30681793 | Chr03 | T | C | 6.82279E-09 | 8.166038 | intergenic | ene:MD03G1222500(dist=4503),gene:MD03G1222600(dist=14398) |
| Chr03_30681826 | Chr03 | C | A | 1.42459E-09 | 8.846311 | intergenic | ene:MD03G1222500(dist=4536),gene:MD03G1222600(dist=14365) |
| Chr03_30681876 | Chr03 | C | G | 1.87921E-08 | 7.726026 | intergenic | ene:MD03G1222500(dist=4586),gene:MD03G1222600(dist=14315) |
| Chr03_30681900 | Chr03 | T | C | 3.56346E-08 | 7.448128 | intergenic | ene:MD03G1222500(dist=4610),gene:MD03G1222600(dist=14291) |
| Chr03_30681908 | Chr03 | C | T | 3.56346E-08 | 7.448128 | intergenic | ene:MD03G1222500(dist=4618),gene:MD03G1222600(dist=14283) |
| Chr03_30681979 | Chr03 | A | G | 1.92079E-08 | 7.71652 | intergenic | ene:MD03G1222500(dist=4689),gene:MD03G1222600(dist=14212) |
| Chr03_30682034 | Chr03 | C | T | 1.05467E-08 | 7.976884 | intergenic | ene:MD03G1222500(dist=4744),gene:MD03G1222600(dist=14157) |
| Chr03_30682446 | Chr03 | T | C | 2.73239E-10 | 9.563458 | intergenic | ene:MD03G1222500(dist=5156),gene:MD03G1222600(dist=13745) |
| Chr03_30682456 | Chr03 | C | A | 6.55063E-11 | 10.18372 | intergenic | ene:MD03G1222500(dist=5166),gene:MD03G1222600(dist=13735) |
| Chr03_30682568 | Chr03 | G | A | 1.55238E-08 | 7.809001 | intergenic | ene:MD03G1222500(dist=5278),gene:MD03G1222600(dist=13623) |
| Chr03_30682603 | Chr03 | G | A | 1.98975E-09 | 8.701202 | intergenic | ene:MD03G1222500(dist=5313),gene:MD03G1222600(dist=13588) |
| Chr03_30684109 | Chr03 | C | T | 1.53797E-08 | 7.813053 | intergenic | ene:MD03G1222500(dist=6819),gene:MD03G1222600(dist=12082) |
| Chr03_30684526 | Chr03 | C | T | 4.17806E-08 | 7.379025 | intergenic | ene:MD03G1222500(dist=7236),gene:MD03G1222600(dist=11665) |
| Chr03_30684532 | Chr03 | C | T | 4.17806E-08 | 7.379025 | intergenic | ene:MD03G1222500(dist=7242),gene:MD03G1222600(dist=11659) |
| Chr03_30684642 | Chr03 | C | T | 1.9291E-08 | 7.714646 | intergenic | ene:MD03G1222500(dist=7352),gene:MD03G1222600(dist=11549) |
| Chr03_30684664 | Chr03 | C | T | 6.06877E-08 | 7.216899 | intergenic | ene:MD03G1222500(dist=7374),gene:MD03G1222600(dist=11527) |
| Chr03_30684688 | Chr03 | G | A | 1.06897E-08 | 7.971034 | intergenic | ene:MD03G1222500(dist=7398),gene:MD03G1222600(dist=11503) |
| Chr03_30684692 | Chr03 | A | C | 1.06897E-08 | 7.971034 | intergenic | ene:MD03G1222500(dist=7402),gene:MD03G1222600(dist=11499) |
| Chr03_30684716 | Chr03 | A | G | 3.64699E-09 | 8.438065 | intergenic | ene:MD03G1222500(dist=7426),gene:MD03G1222600(dist=11475) |
| Chr03_30684963 | Chr03 | G | A | 4.50969E-08 | 7.345853 | intergenic | ene:MD03G1222500(dist=7673),gene:MD03G1222600(dist=11228) |
| Chr03_30684981 | Chr03 | C | A | 1.74001E-08 | 7.759448 | intergenic | ene:MD03G1222500(dist=7691),gene:MD03G1222600(dist=11210) |
| Chr03_30684994 | Chr03 | G | A | 3.39073E-08 | 7.469707 | intergenic | ene:MD03G1222500(dist=7704),gene:MD03G1222600(dist=11197) |
| Chr03_30685017 | Chr03 | C | T | 5.6792E-08 | 7.245713 | intergenic | ene:MD03G1222500(dist=7727),gene:MD03G1222600(dist=11174) |
| Chr03_30685019 | Chr03 | G | T | 5.6792E-08 | 7.245713 | intergenic | ene:MD03G1222500(dist=7729),gene:MD03G1222600(dist=11172) |
| Chr03_30685197 | Chr03 | A | C | 7.09525E-10 | 9.149032 | intergenic | ene:MD03G1222500(dist=7907),gene:MD03G1222600(dist=10994) |
| Chr03_30685211 | Chr03 | A | G | 4.44633E-10 | 9.351998 | intergenic | ene:MD03G1222500(dist=7921),gene:MD03G1222600(dist=10980) |
| Chr03_30685241 | Chr03 | G | A | 8.69353E-09 | 8.060804 | intergenic | ene:MD03G1222500(dist=7951),gene:MD03G1222600(dist=10950) |

| Chr03_30685446 | Chr03 | G | A | 8.19427E-11 | 10.08649 | intergenic |  | ene:MD03G1222500(dist=8156),gene:MD03G1222600(dist=10745) |
| --- | --- | --- | --- | --- | --- | --- | --- | --- |
| Chr03_30685493 | Chr03 | C | T | 1.29478E-08 | 7.887804 | intergenic |  | ene:MD03G1222500(dist=8203),gene:MD03G1222600(dist=10698) |
| Chr03_30685496 | Chr03 | A | G | 1.29478E-08 | 7.887804 | intergenic |  | ene:MD03G1222500(dist=8206),gene:MD03G1222600(dist=10695) |
| Chr03_30685513 | Chr03 | C | T | 1.29478E-08 | 7.887804 | intergenic |  | ene:MD03G1222500(dist=8223),gene:MD03G1222600(dist=10678) |
| Chr03_30685704 | Chr03 | C | A | 3.10774E-08 | 7.507556 | intergenic |  | ene:MD03G1222500(dist=8414),gene:MD03G1222600(dist=10487) |
| Chr03_30686005 | Chr03 | G | A | 5.67912E-08 | 7.245719 | intergenic |  | ene:MD03G1222500(dist=8715),gene:MD03G1222600(dist=10186) |
| Chr03_30686030 | Chr03 | T | C | 2.38438E-08 | 7.622624 | intergenic |  | ene:MD03G1222500(dist=8740),gene:MD03G1222600(dist=10161) |
| Chr03_30686051 | Chr03 | A | G | 2.38438E-08 | 7.622624 | intergenic |  | ene:MD03G1222500(dist=8761),gene:MD03G1222600(dist=10140) |
| Chr03_30686057 | Chr03 | C | G | 2.38438E-08 | 7.622624 | intergenic |  | ene:MD03G1222500(dist=8767),gene:MD03G1222600(dist=10134) |
| Chr03_30686147 | Chr03 | G | A | 2.58103E-09 | 8.588207 | intergenic |  | ene:MD03G1222500(dist=8857),gene:MD03G1222600(dist=10044) |
| Chr03_30686209 | Chr03 | T | C | 2.25603E-08 | 7.646654 | intergenic | gene:MD03G1222500(dist=8919),gene:MD03G1222600(dist=9982) | |
| Chr03_30686220 | Chr03 | T | C | 2.58821E-09 | 8.587 | intergenic | gene:MD03G1222500(dist=8930),gene:MD03G1222600(dist=9971) | |
| Chr03_30686567 | Chr03 | G | T | 6.55668E-11 | 10.18332 | intergenic | gene:MD03G1222500(dist=9277),gene:MD03G1222600(dist=9624) | |
| Chr03_30686569 | Chr03 | C | T | 6.55668E-11 | 10.18332 | intergenic | gene:MD03G1222500(dist=9279),gene:MD03G1222600(dist=9622) | |
| Chr03_30686660 | Chr03 | C | T | 3.9651E-09 | 8.401746 | intergenic | gene:MD03G1222500(dist=9370),gene:MD03G1222600(dist=9531) | |
| Chr03_30686661 | Chr03 | A | G | 1.17824E-10 | 9.928766 | intergenic | gene:MD03G1222500(dist=9371),gene:MD03G1222600(dist=9530) | |
| Chr03_30686753 | Chr03 | A | G | 3.40691E-08 | 7.46764 | intergenic | gene:MD03G1222500(dist=9463),gene:MD03G1222600(dist=9438) | |
| Chr03_30687278 | Chr03 | C | T | 5.90899E-09 | 8.228486 | intergenic | gene:MD03G1222500(dist=9988),gene:MD03G1222600(dist=8913) | |
| Chr03_30687310 | Chr03 | A | T | 9.60749E-09 | 8.01739 | intergenic |  | ene:MD03G1222500(dist=10020),gene:MD03G1222600(dist=8881) |
| Chr03_30687376 | Chr03 | C | T | 9.71576E-10 | 9.012523 | intergenic |  | ene:MD03G1222500(dist=10086),gene:MD03G1222600(dist=8815) |
| Chr03_30687583 | Chr03 | T | A | 3.00724E-09 | 8.521832 | intergenic |  | ene:MD03G1222500(dist=10293),gene:MD03G1222600(dist=8608) |
| Chr03_30691661 | Chr03 | G | T | 5.40784E-10 | 9.266976 | intergenic |  | ene:MD03G1222500(dist=14371),gene:MD03G1222600(dist=4530) |
| Chr03_30691726 | Chr03 | T | C | 1.23795E-09 | 8.907295 | intergenic |  | ene:MD03G1222500(dist=14436),gene:MD03G1222600(dist=4465) |
| Chr03_30691727 | Chr03 | A | G | 2.1483E-10 | 9.667904 | intergenic |  | ene:MD03G1222500(dist=14437),gene:MD03G1222600(dist=4464) |
| Chr03_30691740 | Chr03 | A | T | 1.32489E-10 | 9.877819 | intergenic |  | ene:MD03G1222500(dist=14450),gene:MD03G1222600(dist=4451) |
| Chr03_30691848 | Chr03 | G | A | 1.18192E-10 | 9.927412 | intergenic |  | ene:MD03G1222500(dist=14558),gene:MD03G1222600(dist=4343) |
| Chr03_30691853 | Chr03 | T | C | 1.18192E-10 | 9.927412 | intergenic |  | ene:MD03G1222500(dist=14563),gene:MD03G1222600(dist=4338) |
| Chr03_30692265 | Chr03 | C | A | 7.48007E-09 | 8.126094 | intergenic |  | ene:MD03G1222500(dist=14975),gene:MD03G1222600(dist=3926) |
| Chr03_30692436 | Chr03 | A | C | 6.01749E-12 | 11.22058 | intergenic |  | ene:MD03G1222500(dist=15146),gene:MD03G1222600(dist=3755) |
| Chr03_30692449 | Chr03 | G | A | 3.19167E-12 | 11.49598 | intergenic |  | ene:MD03G1222500(dist=15159),gene:MD03G1222600(dist=3742) |
| Chr03_30692530 | Chr03 | C | T | 1.15458E-09 | 8.937576 | intergenic |  | ene:MD03G1222500(dist=15240),gene:MD03G1222600(dist=3661) |
| Chr03_30692539 | Chr03 | C | T | 1.01844E-11 | 10.99206 | intergenic |  | ene:MD03G1222500(dist=15249),gene:MD03G1222600(dist=3652) |
| Chr03_30692623 | Chr03 | A | G | 1.02239E-11 | 10.99038 | intergenic |  | ene:MD03G1222500(dist=15333),gene:MD03G1222600(dist=3568) |
| Chr03_30692904 | Chr03 | A | T | 3.12271E-08 | 7.505468 | intergenic |  | ene:MD03G1222500(dist=15614),gene:MD03G1222600(dist=3287) |
| Chr03_30693039 | Chr03 | G | C | 5.38033E-11 | 10.26919 | intergenic |  | ene:MD03G1222500(dist=15749),gene:MD03G1222600(dist=3152) |
| Chr03_30693466 | Chr03 | T | A | 4.20189E-11 | 10.37656 | intergenic |  | ene:MD03G1222500(dist=16176),gene:MD03G1222600(dist=2725) |
| Chr03_30693490 | Chr03 | C | A | 4.20189E-11 | 10.37656 | intergenic |  | ene:MD03G1222500(dist=16200),gene:MD03G1222600(dist=2701) |
| Chr03_30693539 | Chr03 | T | G | 4.88243E-10 | 9.311364 | intergenic |  | ene:MD03G1222500(dist=16249),gene:MD03G1222600(dist=2652) |
| Chr03_30693570 | Chr03 | G | A | 5.89875E-08 | 7.22924 | intergenic |  | ene:MD03G1222500(dist=16280),gene:MD03G1222600(dist=2621) |
| Chr03_30693649 | Chr03 | G | A | 2.00274E-08 | 7.698375 | intergenic |  | ene:MD03G1222500(dist=16359),gene:MD03G1222600(dist=2542) |
| Chr03_30693906 | Chr03 | A | T | 8.30862E-12 | 11.08047 | intergenic |  | ene:MD03G1222500(dist=16616),gene:MD03G1222600(dist=2285) |
| Chr03_30694164 | Chr03 | G | A | 2.69266E-08 | 7.569819 | intergenic |  | ene:MD03G1222500(dist=16874),gene:MD03G1222600(dist=2027) |
| Chr03_30694169 | Chr03 | G | A | 3.67974E-10 | 9.434182 | intergenic |  | ene:MD03G1222500(dist=16879),gene:MD03G1222600(dist=2022) |
| Chr03_30694170 | Chr03 | A | T | 4.58679E-10 | 9.338491 | intergenic |  | ene:MD03G1222500(dist=16880),gene:MD03G1222600(dist=2021) |
| Chr03_30694193 | Chr03 | T | A | 9.62161E-09 | 8.016752 | intergenic |  | ene:MD03G1222500(dist=16903),gene:MD03G1222600(dist=1998) |
| Chr03_30694326 | Chr03 | A | C | 1.01979E-11 | 10.99149 | intergenic |  | ene:MD03G1222500(dist=17036),gene:MD03G1222600(dist=1865) |
| Chr03_30694366 | Chr03 | C | G | 1.98215E-10 | 9.702863 | intergenic |  | ene:MD03G1222500(dist=17076),gene:MD03G1222600(dist=1825) |
| Chr03_30694370 | Chr03 | C | T | 5.76127E-10 | 9.239482 | intergenic |  | ene:MD03G1222500(dist=17080),gene:MD03G1222600(dist=1821) |
| Chr03_30694499 | Chr03 | A | G | 1.14976E-09 | 8.939394 | intergenic |  | ene:MD03G1222500(dist=17209),gene:MD03G1222600(dist=1692) |
| Chr03_30694522 | Chr03 | T | C | 1.14976E-09 | 8.939394 | intergenic |  | ene:MD03G1222500(dist=17232),gene:MD03G1222600(dist=1669) |
| Chr03_30694759 | Chr03 | G | A | 1.37094E-08 | 7.862981 | intergenic |  | ene:MD03G1222500(dist=17469),gene:MD03G1222600(dist=1432) |
| Chr03_30694778 | Chr03 | A | G | 7.00229E-10 | 9.15476 | intergenic |  | ene:MD03G1222500(dist=17488),gene:MD03G1222600(dist=1413) |
| Chr03_30695114 | Chr03 | G | T | 1.88352E-08 | 7.72503 | intergenic |  | ene:MD03G1222500(dist=17824),gene:MD03G1222600(dist=1077) |
| Chr03_30695180 | Chr03 | G | T | 2.9886E-09 | 8.524532 | intergenic |  | ene:MD03G1222500(dist=17890),gene:MD03G1222600(dist=1011) |
| Chr03_30695184 | Chr03 | G | A | 2.9886E-09 | 8.524532 | intergenic |  | ene:MD03G1222500(dist=17894),gene:MD03G1222600(dist=1007) |
| Chr03_30695272 | Chr03 | A | G | 2.45451E-08 | 7.610035 | downstream |  | gene:MD03G1222600(dist=919) |
| Chr03_30695380 | Chr03 | G | A | 1.93444E-08 | 7.713444 | downstream |  | gene:MD03G1222600(dist=811) |
| Chr03_30695392 | Chr03 | C | T | 1.93444E-08 | 7.713444 | downstream |  | gene:MD03G1222600(dist=799) |
| Chr03_30695619 | Chr03 | T | C | 2.62739E-08 | 7.580475 | downstream |  | gene:MD03G1222600(dist=572) |
| Chr03_30695666 | Chr03 | T | C | 6.73667E-08 | 7.171555 | downstream |  | gene:MD03G1222600(dist=525) |
| Chr03_30695699 | Chr03 | G | A | 8.17861E-09 | 8.08732 | downstream |  | gene:MD03G1222600(dist=492) |
| Chr03_30695748 | Chr03 | A | T | 1.79063E-09 | 8.746994 | downstream |  | gene:MD03G1222600(dist=443) |
| Chr03_30695764 | Chr03 | T | C | 2.98763E-09 | 8.524673 | downstream |  | gene:MD03G1222600(dist=427) |
| Chr03_30695852 | Chr03 | A | C | 4.456E-09 | 8.351055 | downstream |  | gene:MD03G1222600(dist=339) |
| Chr03_30695868 | Chr03 | T | A | 2.50935E-09 | 8.600439 | downstream |  | gene:MD03G1222600(dist=323) |
| Chr03_30696024 | Chr03 | A | G | 9.79804E-09 | 8.008861 | downstream |  | gene:MD03G1222600(dist=167) |
| Chr03_30696038 | Chr03 | A | G | 9.79804E-09 | 8.008861 | downstream |  | gene:MD03G1222600(dist=153) |
| Chr03_30696066 | Chr03 | A | G | 3.18835E-09 | 8.496435 | downstream |  | gene:MD03G1222600(dist=125) |
| Chr03_30696070 | Chr03 | T | C | 3.18835E-09 | 8.496435 | downstream |  | gene:MD03G1222600(dist=121) |
| Chr03_30696162 | Chr03 | T | A | 5.469E-09 | 8.262092 | downstream |  | gene:MD03G1222600(dist=29) |
| Chr03_30696170 | Chr03 | T | C | 2.81724E-09 | 8.550176 | downstream |  | gene:MD03G1222600(dist=21) |
| Chr03_30696278 | Chr03 | C | A | 3.27896E-08 | 7.484264 | UTR3 |  | gene:MD03G1222600(mRNA:MD03G1222600:c.*381G>T) |
| Chr03_30696317 | Chr03 | A | C | 1.37959E-08 | 7.860249 | UTR3 |  | gene:MD03G1222600(mRNA:MD03G1222600:c.*342T>G) |
| Chr03_30696511 | Chr03 | C | A | 1.71219E-09 | 8.766447 | UTR3 |  | gene:MD03G1222600(mRNA:MD03G1222600:c.*148G>T) |
| Chr03_30696583 | Chr03 | C | T | 1.40755E-08 | 7.851538 | UTR3 |  | gene:MD03G1222600(mRNA:MD03G1222600:c.*76G>A) |
| Chr03_30696595 | Chr03 | A | T | 1.40755E-08 | 7.851538 | UTR3 |  | gene:MD03G1222600(mRNA:MD03G1222600:c.*64T>A) |
| Chr03_30696690 | Chr03 | A | T | 2.82366E-11 | 10.54919 | nonsynonymous SNV | | e:MD03G1222600:mRNA:MD03G1222600:exon3:c.T1064A:p.M355K, |
| Chr03_30696714 | Chr03 | C | T | 2.82366E-11 | 10.54919 | nonsynonymous SNV | | e:MD03G1222600:mRNA:MD03G1222600:exon3:c.G1040A:p.R347Q, |
| Chr03_30696716 | Chr03 | G | A | 2.82366E-11 | 10.54919 | synonymous SNV | ne:MD03G1222600:mRNA:MD03G1222600:exon3:c.C1038T:p.F346F, | |
| Chr03_30696767 | Chr03 | C | A | 3.84037E-09 | 8.415627 | synonymous SNV | ne:MD03G1222600:mRNA:MD03G1222600:exon3:c.G987T:p.T329T, | |
| Chr03_30696772 | Chr03 | G | C | 3.84037E-09 | 8.415627 | nonsynonymous SNV | | ne:MD03G1222600:mRNA:MD03G1222600:exon3:c.C982G:p.Q328E, |
| Chr03_30696781 | Chr03 | G | C | 3.39353E-10 | 9.469348 | nonsynonymous SNV | | ne:MD03G1222600:mRNA:MD03G1222600:exon3:c.C973G:p.Q325E, |
| Chr03_30696805 | Chr03 | T | A | 9.94978E-11 | 10.00219 | nonsynonymous SNV | | ne:MD03G1222600:mRNA:MD03G1222600:exon3:c.A949T:p.S317C, |
| Chr03_30696840 | Chr03 | G | T | 3.0311E-10 | 9.518399 | nonsynonymous SNV | | ne:MD03G1222600:mRNA:MD03G1222600:exon3:c.C914A:p.A305D, |
| Chr03_30696903 | Chr03 | T | A | 2.20011E-11 | 10.65756 | nonsynonymous SNV | | ne:MD03G1222600:mRNA:MD03G1222600:exon3:c.A851T:p.D284V, |
| Chr03_30697286 | Chr03 | A | T | 1.00384E-10 | 9.998336 | intronic |  | gene:MD03G1222600 |

| Chr03_30697295 | Chr03 | T | C | 1.23985E-09 | 8.906631 | intronic | gene:MD03G1222600 |
| --- | --- | --- | --- | --- | --- | --- | --- |
| Chr03_30697306 | Chr03 | G | T | 7.22007E-08 | 7.141459 | intronic | gene:MD03G1222600 |
| Chr03_30697313 | Chr03 | T | C | 4.56185E-10 | 9.340859 | intronic | gene:MD03G1222600 |
| Chr03_30697376 | Chr03 | A | C | 7.56109E-10 | 9.121415 | intronic | gene:MD03G1222600 |
| Chr03_30697434 | Chr03 | G | C | 7.56109E-10 | 9.121415 | nonsynonymous SNV | ne:MD03G1222600:mRNA:MD03G1222600:exon2:c.C477G:p.N159K, |
| Chr03_30697608 | Chr03 | C | A | 3.80014E-11 | 10.4202 | synonymous SNV | ne:MD03G1222600:mRNA:MD03G1222600:exon2:c.G303T:p.A101A, |
| Chr03_30697760 | Chr03 | C | T | 7.24478E-12 | 11.13998 | intronic | gene:MD03G1222600 |
| Chr03_30697761 | Chr03 | G | T | 7.24478E-12 | 11.13998 | intronic | gene:MD03G1222600 |
| Chr03_30697907 | Chr03 | A | C | 9.61529E-11 | 10.01704 | nonsynonymous SNV | ne:MD03G1222600:mRNA:MD03G1222600:exon1:c.T145G:p.S49A, |
| Chr03_30697953 | Chr03 | C | A | 1.1394E-10 | 9.943324 | synonymous SNV | ene:MD03G1222600:mRNA:MD03G1222600:exon1:c.G99T:p.P33P, |
| Chr03_30698039 | Chr03 | C | A | 7.61798E-11 | 10.11816 | nonsynonymous SNV | ene:MD03G1222600:mRNA:MD03G1222600:exon1:c.G13T:p.D5Y, |
| Chr03_30698053 | Chr03 | C | T | 3.56389E-10 | 9.448076 | UTR5 | gene:MD03G1222600(mRNA:MD03G1222600:c.-2G>A) |
| Chr03_30698157 | Chr03 | T | C | 6.4837E-12 | 11.18818 | UTR5 | gene:MD03G1222600(mRNA:MD03G1222600:c.-106A>G) |
| Chr03_30698221 | Chr03 | C | A | 2.12402E-10 | 9.672842 | upstream | gene:MD03G1222600(dist=5) |
| Chr03_30698228 | Chr03 | A | C | 4.77745E-11 | 10.3208 | upstream | gene:MD03G1222600(dist=12) |
| Chr03_30698285 | Chr03 | C | G | 1.39215E-11 | 10.85631 | upstream | gene:MD03G1222600(dist=69) |
| Chr03_30698377 | Chr03 | A | C | 2.05692E-11 | 10.68678 | upstream | gene:MD03G1222600(dist=161) |
| Chr03_30698437 | Chr03 | G | T | 7.14716E-09 | 8.145867 | upstream | gene:MD03G1222600(dist=221) |
| Chr03_30698473 | Chr03 | G | A | 1.31357E-09 | 8.881546 | upstream | gene:MD03G1222600(dist=257) |
| Chr03_30698480 | Chr03 | T | C | 9.49914E-10 | 9.022316 | upstream | gene:MD03G1222600(dist=264) |
| Chr03_30698504 | Chr03 | G | A | 1.06235E-10 | 9.973731 | upstream | gene:MD03G1222600(dist=288) |
| Chr03_30698521 | Chr03 | C | T | 6.92789E-09 | 8.159399 | upstream | gene:MD03G1222600(dist=305) |
| Chr03_30698585 | Chr03 | A | G | 2.273E-08 | 7.6434 | upstream | gene:MD03G1222600(dist=369) |
| Chr03_30698933 | Chr03 | C | T | 4.23417E-08 | 7.373232 | upstream | gene:MD03G1222600(dist=717) |
| Chr03_30698996 | Chr03 | G | A | 4.72434E-08 | 7.325658 | upstream | gene:MD03G1222600(dist=780) |
| Chr03_30699004 | Chr03 | C | T | 3.17407E-08 | 7.498383 | upstream | gene:MD03G1222600(dist=788) |
| Chr03_30699007 | Chr03 | T | C | 9.25782E-10 | 9.033491 | upstream | gene:MD03G1222600(dist=791) |
| Chr03_30699018 | Chr03 | C | T | 1.63198E-08 | 7.787286 | upstream | gene:MD03G1222600(dist=802) |
| Chr03_30699029 | Chr03 | C | T | 1.29941E-08 | 7.886252 | upstream | gene:MD03G1222600(dist=813) |
| Chr03_30699061 | Chr03 | T | C | 8.74821E-09 | 8.058081 | upstream | gene:MD03G1222600(dist=845) |
| Chr03_30699066 | Chr03 | C | T | 1.6602E-09 | 8.77984 | upstream | gene:MD03G1222600(dist=850) |
| Chr03_30699099 | Chr03 | C | T | 2.39931E-08 | 7.619914 | upstream | gene:MD03G1222600(dist=883) |
| Chr03_30699119 | Chr03 | G | T | 3.2693E-08 | 7.485545 | upstream | gene:MD03G1222600(dist=903) |
| Chr03_30699124 | Chr03 | G | A | 3.2693E-08 | 7.485545 | upstream | gene:MD03G1222600(dist=908) |
| Chr03_30700359 | Chr03 | A | G | 1.40305E-09 | 8.852926 | intergenic | ene:MD03G1222600(dist=2143),gene:MD03G1222700(dist=38240) |
| Chr03_30700433 | Chr03 | C | T | 2.02784E-09 | 8.692965 | intergenic | ene:MD03G1222600(dist=2217),gene:MD03G1222700(dist=38166) |
| Chr03_30700572 | Chr03 | G | A | 9.5936E-11 | 10.01802 | intergenic | ene:MD03G1222600(dist=2356),gene:MD03G1222700(dist=38027) |
| Chr03_30700643 | Chr03 | C | T | 1.67035E-12 | 11.77719 | intergenic | ene:MD03G1222600(dist=2427),gene:MD03G1222700(dist=37956) |
| Chr03_30700665 | Chr03 | C | G | 7.47058E-10 | 9.126646 | intergenic | ene:MD03G1222600(dist=2449),gene:MD03G1222700(dist=37934) |
| Chr03_30700731 | Chr03 | C | T | 6.8905E-09 | 8.161749 | intergenic | ene:MD03G1222600(dist=2515),gene:MD03G1222700(dist=37868) |
| Chr03_30700733 | Chr03 | T | C | 6.8905E-09 | 8.161749 | intergenic | ene:MD03G1222600(dist=2517),gene:MD03G1222700(dist=37866) |
| Chr03_30700756 | Chr03 | G | A | 5.5071E-09 | 8.259077 | intergenic | ene:MD03G1222600(dist=2540),gene:MD03G1222700(dist=37843) |
| Chr03_30700757 | Chr03 | G | A | 5.5071E-09 | 8.259077 | intergenic | ene:MD03G1222600(dist=2541),gene:MD03G1222700(dist=37842) |
| Chr03_30700772 | Chr03 | C | T | 8.96661E-10 | 9.047372 | intergenic | ene:MD03G1222600(dist=2556),gene:MD03G1222700(dist=37827) |
| Chr03_30700774 | Chr03 | C | T | 8.96661E-10 | 9.047372 | intergenic | ene:MD03G1222600(dist=2558),gene:MD03G1222700(dist=37825) |
| Chr03_30700779 | Chr03 | C | T | 9.08263E-09 | 8.041789 | intergenic | ene:MD03G1222600(dist=2563),gene:MD03G1222700(dist=37820) |
| Chr03_30700787 | Chr03 | A | T | 2.71124E-10 | 9.566832 | intergenic | ene:MD03G1222600(dist=2571),gene:MD03G1222700(dist=37812) |
| Chr03_30700791 | Chr03 | A | T | 3.47355E-09 | 8.459226 | intergenic | ene:MD03G1222600(dist=2575),gene:MD03G1222700(dist=37808) |
| Chr03_30700799 | Chr03 | A | C | 5.22781E-12 | 11.28168 | intergenic | ene:MD03G1222600(dist=2583),gene:MD03G1222700(dist=37800) |
| Chr03_30700805 | Chr03 | T | A | 1.06782E-09 | 8.971503 | intergenic | ene:MD03G1222600(dist=2589),gene:MD03G1222700(dist=37794) |
| Chr03_30700819 | Chr03 | T | A | 2.40099E-10 | 9.619609 | intergenic | ene:MD03G1222600(dist=2603),gene:MD03G1222700(dist=37780) |
| Chr03_30700837 | Chr03 | T | C | 9.2822E-11 | 10.03235 | intergenic | ene:MD03G1222600(dist=2621),gene:MD03G1222700(dist=37762) |
| Chr03_30700875 | Chr03 | A | G | 1.63851E-08 | 7.78555 | intergenic | ene:MD03G1222600(dist=2659),gene:MD03G1222700(dist=37724) |
| Chr03_30700971 | Chr03 | G | A | 2.49013E-10 | 9.603779 | intergenic | ene:MD03G1222600(dist=2755),gene:MD03G1222700(dist=37628) |
| Chr03_30701024 | Chr03 | T | A | 2.60549E-09 | 8.584111 | intergenic | ene:MD03G1222600(dist=2808),gene:MD03G1222700(dist=37575) |
| Chr03_30701133 | Chr03 | G | A | 4.27842E-09 | 8.368716 | intergenic | ene:MD03G1222600(dist=2917),gene:MD03G1222700(dist=37466) |
| Chr03_30701184 | Chr03 | A | G | 4.27842E-09 | 8.368716 | intergenic | ene:MD03G1222600(dist=2968),gene:MD03G1222700(dist=37415) |
| Chr03_30701199 | Chr03 | C | T | 4.27842E-09 | 8.368716 | intergenic | ene:MD03G1222600(dist=2983),gene:MD03G1222700(dist=37400) |
| Chr03_30702098 | Chr03 | G | A | 2.84638E-09 | 8.545708 | intergenic | ene:MD03G1222600(dist=3882),gene:MD03G1222700(dist=36501) |
| Chr03_30702109 | Chr03 | T | C | 7.84768E-09 | 8.105259 | intergenic | ene:MD03G1222600(dist=3893),gene:MD03G1222700(dist=36490) |
| Chr03_30702121 | Chr03 | G | A | 7.84768E-09 | 8.105259 | intergenic | ene:MD03G1222600(dist=3905),gene:MD03G1222700(dist=36478) |
| Chr03_30702131 | Chr03 | A | G | 7.84768E-09 | 8.105259 | intergenic | ene:MD03G1222600(dist=3915),gene:MD03G1222700(dist=36468) |
| Chr03_30702154 | Chr03 | G | A | 7.17863E-09 | 8.143959 | intergenic | ene:MD03G1222600(dist=3938),gene:MD03G1222700(dist=36445) |
| Chr03_30702228 | Chr03 | A | G | 2.69559E-08 | 7.569347 | intergenic | ene:MD03G1222600(dist=4012),gene:MD03G1222700(dist=36371) |
| Chr03_30702234 | Chr03 | A | T | 2.69559E-08 | 7.569347 | intergenic | ene:MD03G1222600(dist=4018),gene:MD03G1222700(dist=36365) |
| Chr03_30702240 | Chr03 | C | T | 2.56342E-08 | 7.591181 | intergenic | ene:MD03G1222600(dist=4024),gene:MD03G1222700(dist=36359) |
| Chr03_30702446 | Chr03 | T | C | 4.22471E-08 | 7.374203 | intergenic | ene:MD03G1222600(dist=4230),gene:MD03G1222700(dist=36153) |
| Chr03_30702630 | Chr03 | A | G | 1.86204E-08 | 7.73001 | intergenic | ene:MD03G1222600(dist=4414),gene:MD03G1222700(dist=35969) |
| Chr03_30702648 | Chr03 | C | T | 2.70059E-08 | 7.568542 | intergenic | ene:MD03G1222600(dist=4432),gene:MD03G1222700(dist=35951) |
| Chr03_30702958 | Chr03 | A | G | 9.07871E-09 | 8.041976 | intergenic | ene:MD03G1222600(dist=4742),gene:MD03G1222700(dist=35641) |
| Chr03_30702965 | Chr03 | A | G | 9.07871E-09 | 8.041976 | intergenic | ene:MD03G1222600(dist=4749),gene:MD03G1222700(dist=35634) |
| Chr03_30702981 | Chr03 | G | T | 9.07871E-09 | 8.041976 | intergenic | ene:MD03G1222600(dist=4765),gene:MD03G1222700(dist=35618) |
| Chr03_30702986 | Chr03 | C | T | 8.37574E-09 | 8.076977 | intergenic | ene:MD03G1222600(dist=4770),gene:MD03G1222700(dist=35613) |
| Chr03_30703043 | Chr03 | C | T | 7.21805E-10 | 9.14158 | intergenic | ene:MD03G1222600(dist=4827),gene:MD03G1222700(dist=35556) |
| Chr03_30703052 | Chr03 | C | A | 5.11807E-10 | 9.290894 | intergenic | ene:MD03G1222600(dist=4836),gene:MD03G1222700(dist=35547) |
| Chr03_30703077 | Chr03 | C | A | 2.20576E-08 | 7.656443 | intergenic | ene:MD03G1222600(dist=4861),gene:MD03G1222700(dist=35522) |
| Chr03_30703088 | Chr03 | G | T | 8.83708E-09 | 8.053691 | intergenic | ene:MD03G1222600(dist=4872),gene:MD03G1222700(dist=35511) |
| Chr03_30703103 | Chr03 | C | T | 5.21264E-08 | 7.282942 | intergenic | ene:MD03G1222600(dist=4887),gene:MD03G1222700(dist=35496) |
| Chr03_30705962 | Chr03 | C | T | 3.92033E-08 | 7.406678 | intergenic | ene:MD03G1222600(dist=7746),gene:MD03G1222700(dist=32637) |
| Chr03_30706150 | Chr03 | G | A | 6.22589E-08 | 7.205799 | intergenic | ene:MD03G1222600(dist=7934),gene:MD03G1222700(dist=32449) |
| Chr03_30706153 | Chr03 | G | A | 3.70636E-08 | 7.431052 | intergenic | ene:MD03G1222600(dist=7937),gene:MD03G1222700(dist=32446) |
| Chr03_30706165 | Chr03 | T | A | 3.76198E-08 | 7.424584 | intergenic | ene:MD03G1222600(dist=7949),gene:MD03G1222700(dist=32434) |
| Chr03_30706167 | Chr03 | C | T | 3.76198E-08 | 7.424584 | intergenic | ene:MD03G1222600(dist=7951),gene:MD03G1222700(dist=32432) |
| Chr03_30706177 | Chr03 | T | A | 4.35059E-08 | 7.361451 | intergenic | ene:MD03G1222600(dist=7961),gene:MD03G1222700(dist=32422) |
| Chr03_30706326 | Chr03 | A | G | 1.99325E-10 | 9.700438 | intergenic | ene:MD03G1222600(dist=8110),gene:MD03G1222700(dist=32273) |
| Chr03_30706359 | Chr03 | C | T | 2.28069E-09 | 8.641934 | intergenic | ene:MD03G1222600(dist=8143),gene:MD03G1222700(dist=32240) |

| Chr03_30706399 | Chr03 | T | G | 1.75995E-08 | 7.7545 | intergenic | ene:MD03G1222600(dist=8183),gene:MD03G1222700(dist=32200) |
| --- | --- | --- | --- | --- | --- | --- | --- |
| Chr03_30706400 | Chr03 | A | G | 1.75995E-08 | 7.7545 | intergenic | ene:MD03G1222600(dist=8184),gene:MD03G1222700(dist=32199) |
| Chr03_30706411 | Chr03 | A | G | 1.75995E-08 | 7.7545 | intergenic | ene:MD03G1222600(dist=8195),gene:MD03G1222700(dist=32188) |
| Chr03_30706430 | Chr03 | G | A | 2.94236E-08 | 7.531304 | intergenic | ene:MD03G1222600(dist=8214),gene:MD03G1222700(dist=32169) |
| Chr03_30706538 | Chr03 | C | T | 6.11594E-12 | 11.21354 | intergenic | ene:MD03G1222600(dist=8322),gene:MD03G1222700(dist=32061) |
| Chr03_30706539 | Chr03 | C | A | 6.11594E-12 | 11.21354 | intergenic | ene:MD03G1222600(dist=8323),gene:MD03G1222700(dist=32060) |
| Chr03_30706544 | Chr03 | G | A | 3.59225E-08 | 7.444633 | intergenic | ene:MD03G1222600(dist=8328),gene:MD03G1222700(dist=32055) |
| Chr03_30706579 | Chr03 | A | T | 9.0329E-10 | 9.044173 | intergenic | ene:MD03G1222600(dist=8363),gene:MD03G1222700(dist=32020) |
| Chr03_30706604 | Chr03 | G | C | 1.23499E-09 | 8.908335 | intergenic | ene:MD03G1222600(dist=8388),gene:MD03G1222700(dist=31995) |
| Chr03_30706621 | Chr03 | G | A | 5.4727E-10 | 9.261798 | intergenic | ene:MD03G1222600(dist=8405),gene:MD03G1222700(dist=31978) |
| Chr03_30706642 | Chr03 | C | T | 8.37368E-10 | 9.077084 | intergenic | ene:MD03G1222600(dist=8426),gene:MD03G1222700(dist=31957) |
| Chr03_30706655 | Chr03 | T | C | 8.07634E-11 | 10.09279 | intergenic | ene:MD03G1222600(dist=8439),gene:MD03G1222700(dist=31944) |
| Chr03_30706656 | Chr03 | C | T | 8.07634E-11 | 10.09279 | intergenic | ene:MD03G1222600(dist=8440),gene:MD03G1222700(dist=31943) |
| Chr03_30706695 | Chr03 | G | A | 1.64472E-09 | 8.783907 | intergenic | ene:MD03G1222600(dist=8479),gene:MD03G1222700(dist=31904) |
| Chr03_30706708 | Chr03 | T | C | 1.64472E-09 | 8.783907 | intergenic | ene:MD03G1222600(dist=8492),gene:MD03G1222700(dist=31891) |
| Chr03_30706735 | Chr03 | A | G | 8.91934E-10 | 9.049667 | intergenic | ene:MD03G1222600(dist=8519),gene:MD03G1222700(dist=31864) |
| Chr03_30706742 | Chr03 | G | A | 8.91934E-10 | 9.049667 | intergenic | ene:MD03G1222600(dist=8526),gene:MD03G1222700(dist=31857) |
| Chr03_30706846 | Chr03 | T | C | 1.39317E-09 | 8.855996 | intergenic | ene:MD03G1222600(dist=8630),gene:MD03G1222700(dist=31753) |
| Chr03_30706958 | Chr03 | C | T | 2.52195E-10 | 9.598263 | intergenic | ene:MD03G1222600(dist=8742),gene:MD03G1222700(dist=31641) |
| Chr03_30706967 | Chr03 | T | A | 3.47112E-11 | 10.45953 | intergenic | ene:MD03G1222600(dist=8751),gene:MD03G1222700(dist=31632) |
| Chr03_30706979 | Chr03 | A | G | 1.93918E-10 | 9.712381 | intergenic | ene:MD03G1222600(dist=8763),gene:MD03G1222700(dist=31620) |
| Chr03_30707012 | Chr03 | T | G | 1.09756E-09 | 8.959571 | intergenic | ene:MD03G1222600(dist=8796),gene:MD03G1222700(dist=31587) |
| Chr03_30707020 | Chr03 | A | G | 1.6337E-09 | 8.786829 | intergenic | ene:MD03G1222600(dist=8804),gene:MD03G1222700(dist=31579) |
| Chr03_30707072 | Chr03 | T | C | 6.13246E-09 | 8.212365 | intergenic | ene:MD03G1222600(dist=8856),gene:MD03G1222700(dist=31527) |
| Chr03_30707093 | Chr03 | G | T | 1.29497E-09 | 8.887741 | intergenic | ene:MD03G1222600(dist=8877),gene:MD03G1222700(dist=31506) |
| Chr03_30707099 | Chr03 | A | C | 7.03397E-09 | 8.152799 | intergenic | ene:MD03G1222600(dist=8883),gene:MD03G1222700(dist=31500) |
| Chr03_30707101 | Chr03 | G | A | 7.03397E-09 | 8.152799 | intergenic | ene:MD03G1222600(dist=8885),gene:MD03G1222700(dist=31498) |
| Chr03_30707531 | Chr03 | G | A | 2.37594E-09 | 8.624164 | intergenic | ene:MD03G1222600(dist=9315),gene:MD03G1222700(dist=31068) |
| Chr03_30707532 | Chr03 | C | A | 2.37594E-09 | 8.624164 | intergenic | ene:MD03G1222600(dist=9316),gene:MD03G1222700(dist=31067) |
| Chr03_30707536 | Chr03 | G | T | 5.89811E-10 | 9.229287 | intergenic | ene:MD03G1222600(dist=9320),gene:MD03G1222700(dist=31063) |
| Chr03_30707544 | Chr03 | T | A | 5.89811E-10 | 9.229287 | intergenic | ene:MD03G1222600(dist=9328),gene:MD03G1222700(dist=31055) |
| Chr03_30707562 | Chr03 | C | T | 1.76043E-09 | 8.754381 | intergenic | ene:MD03G1222600(dist=9346),gene:MD03G1222700(dist=31037) |
| Chr03_30707567 | Chr03 | G | A | 1.76043E-09 | 8.754381 | intergenic | ene:MD03G1222600(dist=9351),gene:MD03G1222700(dist=31032) |
| Chr03_30707711 | Chr03 | A | G | 5.25246E-10 | 9.279637 | intergenic | ene:MD03G1222600(dist=9495),gene:MD03G1222700(dist=30888) |
| Chr03_30707735 | Chr03 | G | A | 4.99051E-09 | 8.301855 | intergenic | ene:MD03G1222600(dist=9519),gene:MD03G1222700(dist=30864) |
| Chr03_30707783 | Chr03 | A | G | 7.87681E-12 | 11.10365 | intergenic | ene:MD03G1222600(dist=9567),gene:MD03G1222700(dist=30816) |
| Chr03_30707786 | Chr03 | G | A | 7.87681E-12 | 11.10365 | intergenic | ene:MD03G1222600(dist=9570),gene:MD03G1222700(dist=30813) |
| Chr03_30707793 | Chr03 | A | G | 7.87681E-12 | 11.10365 | intergenic | ene:MD03G1222600(dist=9577),gene:MD03G1222700(dist=30806) |
| Chr03_30707799 | Chr03 | G | C | 2.77331E-11 | 10.557 | intergenic | ene:MD03G1222600(dist=9583),gene:MD03G1222700(dist=30800) |
| Chr03_30707812 | Chr03 | G | A | 1.50493E-11 | 10.82248 | intergenic | ene:MD03G1222600(dist=9596),gene:MD03G1222700(dist=30787) |
| Chr03_30707836 | Chr03 | A | G | 1.23496E-11 | 10.90835 | intergenic | ene:MD03G1222600(dist=9620),gene:MD03G1222700(dist=30763) |
| Chr03_30707858 | Chr03 | C | T | 6.37476E-12 | 11.19554 | intergenic | ene:MD03G1222600(dist=9642),gene:MD03G1222700(dist=30741) |
| Chr03_30707875 | Chr03 | T | C | 3.88163E-08 | 7.410986 | intergenic | ene:MD03G1222600(dist=9659),gene:MD03G1222700(dist=30724) |
| Chr03_30707878 | Chr03 | T | C | 3.01194E-08 | 7.521154 | intergenic | ene:MD03G1222600(dist=9662),gene:MD03G1222700(dist=30721) |
| Chr03_30708218 | Chr03 | A | G | 1.79336E-09 | 8.746333 | intergenic | ene:MD03G1222600(dist=10002),gene:MD03G1222700(dist=30381) |
| Chr03_30708315 | Chr03 | T | C | 4.51137E-09 | 8.345692 | intergenic | ene:MD03G1222600(dist=10099),gene:MD03G1222700(dist=30284) |
| Chr03_30708373 | Chr03 | G | A | 4.54035E-08 | 7.34291 | intergenic | ene:MD03G1222600(dist=10157),gene:MD03G1222700(dist=30226) |
| Chr03_30708378 | Chr03 | G | A | 4.54035E-08 | 7.34291 | intergenic | ene:MD03G1222600(dist=10162),gene:MD03G1222700(dist=30221) |
| Chr03_30709494 | Chr03 | A | T | 4.89247E-08 | 7.310472 | intergenic | ene:MD03G1222600(dist=11278),gene:MD03G1222700(dist=29105) |
| Chr03_30709864 | Chr03 | C | T | 1.35533E-11 | 10.86795 | intergenic | ene:MD03G1222600(dist=11648),gene:MD03G1222700(dist=28735) |
| Chr03_30709888 | Chr03 | T | A | 1.35533E-11 | 10.86795 | intergenic | ene:MD03G1222600(dist=11672),gene:MD03G1222700(dist=28711) |
| Chr03_30709890 | Chr03 | G | A | 1.35533E-11 | 10.86795 | intergenic | ene:MD03G1222600(dist=11674),gene:MD03G1222700(dist=28709) |
| Chr03_30709893 | Chr03 | T | C | 1.35533E-11 | 10.86795 | intergenic | ene:MD03G1222600(dist=11677),gene:MD03G1222700(dist=28706) |
| Chr03_30709906 | Chr03 | G | A | 2.52834E-09 | 8.597164 | intergenic | ene:MD03G1222600(dist=11690),gene:MD03G1222700(dist=28693) |
| Chr03_30709928 | Chr03 | C | T | 3.73112E-10 | 9.428161 | intergenic | ene:MD03G1222600(dist=11712),gene:MD03G1222700(dist=28671) |
| Chr03_30709939 | Chr03 | G | A | 2.17396E-09 | 8.662749 | intergenic | ene:MD03G1222600(dist=11723),gene:MD03G1222700(dist=28660) |
| Chr03_30709943 | Chr03 | T | C | 2.17396E-09 | 8.662749 | intergenic | ene:MD03G1222600(dist=11727),gene:MD03G1222700(dist=28656) |
| Chr03_30709949 | Chr03 | G | A | 1.52584E-09 | 8.816492 | intergenic | ene:MD03G1222600(dist=11733),gene:MD03G1222700(dist=28650) |
| Chr03_30710019 | Chr03 | C | T | 1.7826E-10 | 9.748947 | intergenic | ene:MD03G1222600(dist=11803),gene:MD03G1222700(dist=28580) |
| Chr03_30710052 | Chr03 | G | A | 1.11717E-10 | 9.95188 | intergenic | ene:MD03G1222600(dist=11836),gene:MD03G1222700(dist=28547) |
| Chr03_30710076 | Chr03 | G | C | 1.53822E-10 | 9.81298 | intergenic | ene:MD03G1222600(dist=11860),gene:MD03G1222700(dist=28523) |
| Chr03_30710090 | Chr03 | G | A | 1.03595E-10 | 9.984661 | intergenic | ene:MD03G1222600(dist=11874),gene:MD03G1222700(dist=28509) |
| Chr03_30710220 | Chr03 | C | A | 1.54019E-08 | 7.812426 | intergenic | ene:MD03G1222600(dist=12004),gene:MD03G1222700(dist=28379) |
| Chr03_30710221 | Chr03 | T | A | 1.54019E-08 | 7.812426 | intergenic | ene:MD03G1222600(dist=12005),gene:MD03G1222700(dist=28378) |
| Chr03_30710307 | Chr03 | G | T | 6.78441E-08 | 7.168488 | intergenic | ene:MD03G1222600(dist=12091),gene:MD03G1222700(dist=28292) |
| Chr03_30710501 | Chr03 | G | T | 1.34609E-08 | 7.870925 | intergenic | ene:MD03G1222600(dist=12285),gene:MD03G1222700(dist=28098) |
| Chr03_30710502 | Chr03 | T | G | 1.34609E-08 | 7.870925 | intergenic | ene:MD03G1222600(dist=12286),gene:MD03G1222700(dist=28097) |
| Chr03_30710781 | Chr03 | C | T | 1.28745E-08 | 7.890269 | intergenic | ene:MD03G1222600(dist=12565),gene:MD03G1222700(dist=27818) |
| Chr03_30710795 | Chr03 | C | T | 1.28745E-08 | 7.890269 | intergenic | ene:MD03G1222600(dist=12579),gene:MD03G1222700(dist=27804) |
| Chr03_30710817 | Chr03 | T | G | 1.28745E-08 | 7.890269 | intergenic | ene:MD03G1222600(dist=12601),gene:MD03G1222700(dist=27782) |
| Chr03_30711029 | Chr03 | C | T | 5.39355E-09 | 8.268126 | intergenic | ene:MD03G1222600(dist=12813),gene:MD03G1222700(dist=27570) |
| Chr03_30711045 | Chr03 | A | C | 2.9125E-08 | 7.535734 | intergenic | ene:MD03G1222600(dist=12829),gene:MD03G1222700(dist=27554) |
| Chr03_30711056 | Chr03 | G | A | 2.9125E-08 | 7.535734 | intergenic | ene:MD03G1222600(dist=12840),gene:MD03G1222700(dist=27543) |
| Chr03_30711069 | Chr03 | T | C | 2.9125E-08 | 7.535734 | intergenic | ene:MD03G1222600(dist=12853),gene:MD03G1222700(dist=27530) |
| Chr03_30711071 | Chr03 | T | A | 4.79416E-08 | 7.319287 | intergenic | ene:MD03G1222600(dist=12855),gene:MD03G1222700(dist=27528) |
| Chr03_30711320 | Chr03 | G | A | 4.24383E-09 | 8.372242 | intergenic | ene:MD03G1222600(dist=13104),gene:MD03G1222700(dist=27279) |
| Chr03_30711351 | Chr03 | C | A | 8.74685E-09 | 8.058149 | intergenic | ene:MD03G1222600(dist=13135),gene:MD03G1222700(dist=27248) |
| Chr03_30711402 | Chr03 | A | G | 2.87822E-08 | 7.540876 | intergenic | ene:MD03G1222600(dist=13186),gene:MD03G1222700(dist=27197) |
| Chr03_30711403 | Chr03 | T | C | 7.57144E-09 | 8.120822 | intergenic | ene:MD03G1222600(dist=13187),gene:MD03G1222700(dist=27196) |
| Chr03_30711419 | Chr03 | C | G | 1.20805E-08 | 7.917915 | intergenic | ene:MD03G1222600(dist=13203),gene:MD03G1222700(dist=27180) |
| Chr03_30711441 | Chr03 | T | C | 1.08757E-08 | 7.963543 | intergenic | ene:MD03G1222600(dist=13225),gene:MD03G1222700(dist=27158) |
| Chr03_30711486 | Chr03 | G | A | 1.42127E-09 | 8.847323 | intergenic | ene:MD03G1222600(dist=13270),gene:MD03G1222700(dist=27113) |
| Chr03_30711493 | Chr03 | G | A | 2.98081E-09 | 8.525666 | intergenic | ene:MD03G1222600(dist=13277),gene:MD03G1222700(dist=27106) |
| Chr03_30711517 | Chr03 | C | A | 3.14205E-10 | 9.502787 | intergenic | ene:MD03G1222600(dist=13301),gene:MD03G1222700(dist=27082) |
| Chr03_30711540 | Chr03 | G | A | 2.42736E-10 | 9.614865 | intergenic | ene:MD03G1222600(dist=13324),gene:MD03G1222700(dist=27059) |
| Chr03_30711567 | Chr03 | T | G | 5.03213E-09 | 8.298248 | intergenic | ene:MD03G1222600(dist=13351),gene:MD03G1222700(dist=27032) |

| Chr03_30711624 | Chr03 | C | T | 3.91245E-08 | 7.407551 | intergenic | ene:MD03G1222600(dist=13408),gene:MD03G1222700(dist=26975) |
| --- | --- | --- | --- | --- | --- | --- | --- |
| Chr03_30711724 | Chr03 | C | T | 5.99218E-08 | 7.222415 | intergenic | ene:MD03G1222600(dist=13508),gene:MD03G1222700(dist=26875) |
| Chr03_30711725 | Chr03 | G | A | 5.99218E-08 | 7.222415 | intergenic | ene:MD03G1222600(dist=13509),gene:MD03G1222700(dist=26874) |
| Chr03_30711758 | Chr03 | T | C | 1.21478E-09 | 8.915501 | intergenic | ene:MD03G1222600(dist=13542),gene:MD03G1222700(dist=26841) |
| Chr03_30711820 | Chr03 | A | G | 6.77416E-10 | 9.169144 | intergenic | ene:MD03G1222600(dist=13604),gene:MD03G1222700(dist=26779) |
| Chr03_30711836 | Chr03 | G | T | 2.80799E-10 | 9.551604 | intergenic | ene:MD03G1222600(dist=13620),gene:MD03G1222700(dist=26763) |
| Chr03_30711860 | Chr03 | A | G | 7.4782E-10 | 9.126203 | intergenic | ene:MD03G1222600(dist=13644),gene:MD03G1222700(dist=26739) |
| Chr03_30711866 | Chr03 | C | T | 1.36244E-10 | 9.865683 | intergenic | ene:MD03G1222600(dist=13650),gene:MD03G1222700(dist=26733) |
| Chr03_30711902 | Chr03 | G | A | 9.20765E-11 | 10.03585 | intergenic | ene:MD03G1222600(dist=13686),gene:MD03G1222700(dist=26697) |
| Chr03_30711963 | Chr03 | C | G | 4.88099E-11 | 10.31149 | intergenic | ene:MD03G1222600(dist=13747),gene:MD03G1222700(dist=26636) |
| Chr03_30711977 | Chr03 | C | T | 3.40555E-11 | 10.46781 | intergenic | ene:MD03G1222600(dist=13761),gene:MD03G1222700(dist=26622) |
| Chr03_30712002 | Chr03 | T | C | 1.51988E-11 | 10.81819 | intergenic | ene:MD03G1222600(dist=13786),gene:MD03G1222700(dist=26597) |
| Chr03_30712003 | Chr03 | G | C | 1.51988E-11 | 10.81819 | intergenic | ene:MD03G1222600(dist=13787),gene:MD03G1222700(dist=26596) |
| Chr03_30712077 | Chr03 | T | C | 1.16612E-08 | 7.933257 | intergenic | ene:MD03G1222600(dist=13861),gene:MD03G1222700(dist=26522) |
| Chr03_30712109 | Chr03 | C | T | 1.342E-11 | 10.87225 | intergenic | ene:MD03G1222600(dist=13893),gene:MD03G1222700(dist=26490) |
| Chr03_30712184 | Chr03 | T | C | 4.37356E-13 | 12.35917 | intergenic | ene:MD03G1222600(dist=13968),gene:MD03G1222700(dist=26415) |
| Chr03_30712204 | Chr03 | T | C | 4.37356E-13 | 12.35917 | intergenic | ene:MD03G1222600(dist=13988),gene:MD03G1222700(dist=26395) |
| Chr03_30712247 | Chr03 | T | C | 9.99663E-11 | 10.00015 | intergenic | ene:MD03G1222600(dist=14031),gene:MD03G1222700(dist=26352) |
| Chr03_30712248 | Chr03 | G | A | 9.99663E-11 | 10.00015 | intergenic | ene:MD03G1222600(dist=14032),gene:MD03G1222700(dist=26351) |
| Chr03_30712261 | Chr03 | A | G | 2.37438E-10 | 9.624449 | intergenic | ene:MD03G1222600(dist=14045),gene:MD03G1222700(dist=26338) |
| Chr03_30712276 | Chr03 | G | T | 2.37438E-10 | 9.624449 | intergenic | ene:MD03G1222600(dist=14060),gene:MD03G1222700(dist=26323) |
| Chr03_30712292 | Chr03 | T | C | 1.28705E-09 | 8.890405 | intergenic | ene:MD03G1222600(dist=14076),gene:MD03G1222700(dist=26307) |
| Chr03_30712304 | Chr03 | A | G | 5.84058E-10 | 9.233544 | intergenic | ene:MD03G1222600(dist=14088),gene:MD03G1222700(dist=26295) |
| Chr03_30712316 | Chr03 | G | A | 2.42073E-09 | 8.616054 | intergenic | ene:MD03G1222600(dist=14100),gene:MD03G1222700(dist=26283) |
| Chr03_30712337 | Chr03 | G | T | 1.6689E-09 | 8.77757 | intergenic | ene:MD03G1222600(dist=14121),gene:MD03G1222700(dist=26262) |
| Chr03_30712342 | Chr03 | C | T | 1.6689E-09 | 8.77757 | intergenic | ene:MD03G1222600(dist=14126),gene:MD03G1222700(dist=26257) |
| Chr03_30712354 | Chr03 | A | G | 1.6689E-09 | 8.77757 | intergenic | ene:MD03G1222600(dist=14138),gene:MD03G1222700(dist=26245) |
| Chr03_30712356 | Chr03 | T | G | 8.23297E-10 | 9.084443 | intergenic | ene:MD03G1222600(dist=14140),gene:MD03G1222700(dist=26243) |
| Chr03_30712473 | Chr03 | C | T | 3.22708E-08 | 7.49119 | intergenic | ene:MD03G1222600(dist=14257),gene:MD03G1222700(dist=26126) |
| Chr03_30712479 | Chr03 | C | T | 3.22708E-08 | 7.49119 | intergenic | ene:MD03G1222600(dist=14263),gene:MD03G1222700(dist=26120) |
| Chr03_30712497 | Chr03 | T | C | 3.22708E-08 | 7.49119 | intergenic | ene:MD03G1222600(dist=14281),gene:MD03G1222700(dist=26102) |
| Chr03_30712526 | Chr03 | A | G | 3.22708E-08 | 7.49119 | intergenic | ene:MD03G1222600(dist=14310),gene:MD03G1222700(dist=26073) |
| Chr03_30712639 | Chr03 | C | A | 3.89564E-09 | 8.409421 | intergenic | ene:MD03G1222600(dist=14423),gene:MD03G1222700(dist=25960) |
| Chr03_30712736 | Chr03 | T | C | 1.14829E-09 | 8.939949 | intergenic | ene:MD03G1222600(dist=14520),gene:MD03G1222700(dist=25863) |
| Chr03_30712738 | Chr03 | A | G | 6.90176E-08 | 7.16104 | intergenic | ene:MD03G1222600(dist=14522),gene:MD03G1222700(dist=25861) |
| Chr03_30712748 | Chr03 | C | T | 9.25956E-10 | 9.03341 | intergenic | ene:MD03G1222600(dist=14532),gene:MD03G1222700(dist=25851) |
| Chr03_30712752 | Chr03 | A | G | 9.25956E-10 | 9.03341 | intergenic | ene:MD03G1222600(dist=14536),gene:MD03G1222700(dist=25847) |
| Chr03_30712767 | Chr03 | G | A | 1.34319E-10 | 9.871864 | intergenic | ene:MD03G1222600(dist=14551),gene:MD03G1222700(dist=25832) |
| Chr03_30712771 | Chr03 | T | C | 1.34319E-10 | 9.871864 | intergenic | ene:MD03G1222600(dist=14555),gene:MD03G1222700(dist=25828) |
| Chr03_30712807 | Chr03 | C | G | 1.29659E-09 | 8.887196 | intergenic | ene:MD03G1222600(dist=14591),gene:MD03G1222700(dist=25792) |
| Chr03_30712818 | Chr03 | G | A | 1.35213E-09 | 8.868981 | intergenic | ene:MD03G1222600(dist=14602),gene:MD03G1222700(dist=25781) |
| Chr03_30712919 | Chr03 | G | A | 6.47911E-08 | 7.188484 | intergenic | ene:MD03G1222600(dist=14703),gene:MD03G1222700(dist=25680) |
| Chr03_30713197 | Chr03 | G | A | 5.18716E-10 | 9.28507 | intergenic | ene:MD03G1222600(dist=14981),gene:MD03G1222700(dist=25402) |
| Chr03_30713200 | Chr03 | G | A | 4.5459E-10 | 9.342381 | intergenic | ene:MD03G1222600(dist=14984),gene:MD03G1222700(dist=25399) |
| Chr03_30713224 | Chr03 | G | T | 6.29321E-09 | 8.201128 | intergenic | ene:MD03G1222600(dist=15008),gene:MD03G1222700(dist=25375) |
| Chr03_30713240 | Chr03 | T | C | 1.66082E-08 | 7.779676 | intergenic | ene:MD03G1222600(dist=15024),gene:MD03G1222700(dist=25359) |
| Chr03_30713255 | Chr03 | T | A | 1.66082E-08 | 7.779676 | intergenic | ene:MD03G1222600(dist=15039),gene:MD03G1222700(dist=25344) |
| Chr03_30713257 | Chr03 | T | C | 2.94397E-08 | 7.531067 | intergenic | ene:MD03G1222600(dist=15041),gene:MD03G1222700(dist=25342) |
| Chr03_30713269 | Chr03 | T | C | 2.94397E-08 | 7.531067 | intergenic | ene:MD03G1222600(dist=15053),gene:MD03G1222700(dist=25330) |
| Chr03_30713439 | Chr03 | C | T | 7.91316E-10 | 9.10165 | intergenic | ene:MD03G1222600(dist=15223),gene:MD03G1222700(dist=25160) |
| Chr03_30713453 | Chr03 | A | G | 1.25895E-10 | 9.899991 | intergenic | ene:MD03G1222600(dist=15237),gene:MD03G1222700(dist=25146) |
| Chr03_30713529 | Chr03 | T | C | 4.18068E-08 | 7.378753 | intergenic | ene:MD03G1222600(dist=15313),gene:MD03G1222700(dist=25070) |
| Chr03_30713704 | Chr03 | T | G | 6.11906E-08 | 7.213315 | intergenic | ene:MD03G1222600(dist=15488),gene:MD03G1222700(dist=24895) |
| Chr03_30713708 | Chr03 | G | A | 3.22427E-08 | 7.491569 | intergenic | ene:MD03G1222600(dist=15492),gene:MD03G1222700(dist=24891) |
| Chr03_30713745 | Chr03 | G | A | 1.98046E-08 | 7.703234 | intergenic | ene:MD03G1222600(dist=15529),gene:MD03G1222700(dist=24854) |
| Chr03_30713749 | Chr03 | C | T | 1.98046E-08 | 7.703234 | intergenic | ene:MD03G1222600(dist=15533),gene:MD03G1222700(dist=24850) |
| Chr03_30713947 | Chr03 | T | C | 4.01001E-08 | 7.396854 | intergenic | ene:MD03G1222600(dist=15731),gene:MD03G1222700(dist=24652) |
| Chr03_30713949 | Chr03 | T | C | 4.01001E-08 | 7.396854 | intergenic | ene:MD03G1222600(dist=15733),gene:MD03G1222700(dist=24650) |
| Chr03_30713990 | Chr03 | C | T | 6.29412E-10 | 9.201065 | intergenic | ene:MD03G1222600(dist=15774),gene:MD03G1222700(dist=24609) |
| Chr03_30714049 | Chr03 | G | A | 5.4749E-08 | 7.261624 | intergenic | ene:MD03G1222600(dist=15833),gene:MD03G1222700(dist=24550) |
| Chr03_30714334 | Chr03 | G | A | 3.20863E-08 | 7.49368 | intergenic | ene:MD03G1222600(dist=16118),gene:MD03G1222700(dist=24265) |
| Chr03_30714398 | Chr03 | T | C | 2.09824E-09 | 8.678144 | intergenic | ene:MD03G1222600(dist=16182),gene:MD03G1222700(dist=24201) |
| Chr03_30714409 | Chr03 | A | G | 6.0835E-09 | 8.215846 | intergenic | ene:MD03G1222600(dist=16193),gene:MD03G1222700(dist=24190) |
| Chr03_30714414 | Chr03 | G | A | 1.70452E-09 | 8.768398 | intergenic | ene:MD03G1222600(dist=16198),gene:MD03G1222700(dist=24185) |
| Chr03_30714480 | Chr03 | T | C | 1.69028E-10 | 9.772042 | intergenic | ene:MD03G1222600(dist=16264),gene:MD03G1222700(dist=24119) |
| Chr03_30714515 | Chr03 | C | T | 6.84171E-10 | 9.164835 | intergenic | ene:MD03G1222600(dist=16299),gene:MD03G1222700(dist=24084) |
| Chr03_30714528 | Chr03 | T | C | 8.19242E-10 | 9.086588 | intergenic | ene:MD03G1222600(dist=16312),gene:MD03G1222700(dist=24071) |
| Chr03_30714529 | Chr03 | G | A | 8.19242E-10 | 9.086588 | intergenic | ene:MD03G1222600(dist=16313),gene:MD03G1222700(dist=24070) |
| Chr03_30714613 | Chr03 | C | T | 8.15168E-09 | 8.088753 | intergenic | ene:MD03G1222600(dist=16397),gene:MD03G1222700(dist=23986) |
| Chr03_30714618 | Chr03 | T | G | 8.15168E-09 | 8.088753 | intergenic | ene:MD03G1222600(dist=16402),gene:MD03G1222700(dist=23981) |
| Chr03_30714632 | Chr03 | A | G | 4.94114E-08 | 7.306172 | intergenic | ene:MD03G1222600(dist=16416),gene:MD03G1222700(dist=23967) |
| Chr03_30714678 | Chr03 | A | T | 4.44038E-09 | 8.35258 | intergenic | ene:MD03G1222600(dist=16462),gene:MD03G1222700(dist=23921) |
| Chr03_30714701 | Chr03 | C | A | 4.44038E-09 | 8.35258 | intergenic | ene:MD03G1222600(dist=16485),gene:MD03G1222700(dist=23898) |
| Chr03_30714870 | Chr03 | G | A | 8.41868E-09 | 8.074756 | intergenic | ene:MD03G1222600(dist=16654),gene:MD03G1222700(dist=23729) |
| Chr03_30714883 | Chr03 | G | A | 7.89022E-09 | 8.102911 | intergenic | ene:MD03G1222600(dist=16667),gene:MD03G1222700(dist=23716) |
| Chr03_30714901 | Chr03 | T | C | 4.89158E-09 | 8.310551 | intergenic | ene:MD03G1222600(dist=16685),gene:MD03G1222700(dist=23698) |
| Chr03_30714905 | Chr03 | A | G | 4.89158E-09 | 8.310551 | intergenic | ene:MD03G1222600(dist=16689),gene:MD03G1222700(dist=23694) |
| Chr03_30714950 | Chr03 | A | G | 4.13497E-08 | 7.383527 | intergenic | ene:MD03G1222600(dist=16734),gene:MD03G1222700(dist=23649) |
| Chr03_30714990 | Chr03 | T | C | 6.26685E-09 | 8.20295 | intergenic | ene:MD03G1222600(dist=16774),gene:MD03G1222700(dist=23609) |
| Chr03_30715012 | Chr03 | C | T | 5.04085E-08 | 7.297496 | intergenic | ene:MD03G1222600(dist=16796),gene:MD03G1222700(dist=23587) |
| Chr03_30715026 | Chr03 | C | A | 5.04085E-08 | 7.297496 | intergenic | ene:MD03G1222600(dist=16810),gene:MD03G1222700(dist=23573) |
| Chr03_30715044 | Chr03 | C | T | 4.62481E-08 | 7.334906 | intergenic | ene:MD03G1222600(dist=16828),gene:MD03G1222700(dist=23555) |
| Chr03_30715087 | Chr03 | T | C | 4.85834E-09 | 8.313512 | intergenic | ene:MD03G1222600(dist=16871),gene:MD03G1222700(dist=23512) |
| Chr03_30715098 | Chr03 | C | T | 4.85834E-09 | 8.313512 | intergenic | ene:MD03G1222600(dist=16882),gene:MD03G1222700(dist=23501) |
| Chr03_30715117 | Chr03 | C | T | 3.01518E-09 | 8.520687 | intergenic | ene:MD03G1222600(dist=16901),gene:MD03G1222700(dist=23482) |
| Chr03_30715516 | Chr03 | G | A | 2.905E-08 | 7.536854 | intergenic | ene:MD03G1222600(dist=17300),gene:MD03G1222700(dist=23083) |

| Chr03_30715526 | Chr03 | A | C | 2.905E-08 | 7.536854 | intergenic | ene:MD03G1222600(dist=17310),gene:MD03G1222700(dist=23073) |
| --- | --- | --- | --- | --- | --- | --- | --- |
| Chr03_30715528 | Chr03 | C | T | 1.28298E-08 | 7.891779 | intergenic | ene:MD03G1222600(dist=17312),gene:MD03G1222700(dist=23071) |
| Chr03_30715533 | Chr03 | C | A | 1.28298E-08 | 7.891779 | intergenic | ene:MD03G1222600(dist=17317),gene:MD03G1222700(dist=23066) |
| Chr03_30715539 | Chr03 | A | G | 1.28298E-08 | 7.891779 | intergenic | ene:MD03G1222600(dist=17323),gene:MD03G1222700(dist=23060) |
| Chr03_30715540 | Chr03 | G | A | 1.28298E-08 | 7.891779 | intergenic | ene:MD03G1222600(dist=17324),gene:MD03G1222700(dist=23059) |
| Chr03_30715544 | Chr03 | G | C | 1.28298E-08 | 7.891779 | intergenic | ene:MD03G1222600(dist=17328),gene:MD03G1222700(dist=23055) |
| Chr03_30715582 | Chr03 | G | A | 3.45749E-08 | 7.461239 | intergenic | ene:MD03G1222600(dist=17366),gene:MD03G1222700(dist=23017) |
| Chr03_30715601 | Chr03 | G | A | 3.45749E-08 | 7.461239 | intergenic | ene:MD03G1222600(dist=17385),gene:MD03G1222700(dist=22998) |
| Chr03_30715729 | Chr03 | A | T | 7.63674E-12 | 11.11709 | intergenic | ene:MD03G1222600(dist=17513),gene:MD03G1222700(dist=22870) |
| Chr03_30715734 | Chr03 | A | G | 2.4433E-11 | 10.61202 | intergenic | ene:MD03G1222600(dist=17518),gene:MD03G1222700(dist=22865) |
| Chr03_30715805 | Chr03 | T | C | 3.40688E-09 | 8.467643 | intergenic | ene:MD03G1222600(dist=17589),gene:MD03G1222700(dist=22794) |
| Chr03_30715807 | Chr03 | A | G | 9.97274E-11 | 10.00119 | intergenic | ene:MD03G1222600(dist=17591),gene:MD03G1222700(dist=22792) |
| Chr03_30715810 | Chr03 | A | G | 9.97274E-11 | 10.00119 | intergenic | ene:MD03G1222600(dist=17594),gene:MD03G1222700(dist=22789) |
| Chr03_30715849 | Chr03 | C | A | 5.80807E-10 | 9.235968 | intergenic | ene:MD03G1222600(dist=17633),gene:MD03G1222700(dist=22750) |
| Chr03_30715864 | Chr03 | C | T | 5.87035E-10 | 9.231336 | intergenic | ene:MD03G1222600(dist=17648),gene:MD03G1222700(dist=22735) |
| Chr03_30715881 | Chr03 | T | C | 3.02379E-08 | 7.519449 | intergenic | ene:MD03G1222600(dist=17665),gene:MD03G1222700(dist=22718) |
| Chr03_30715889 | Chr03 | C | T | 2.43104E-10 | 9.614207 | intergenic | ene:MD03G1222600(dist=17673),gene:MD03G1222700(dist=22710) |
| Chr03_30715904 | Chr03 | G | A | 1.68788E-09 | 8.772659 | intergenic | ene:MD03G1222600(dist=17688),gene:MD03G1222700(dist=22695) |
| Chr03_30715956 | Chr03 | G | T | 1.58982E-11 | 10.79865 | intergenic | ene:MD03G1222600(dist=17740),gene:MD03G1222700(dist=22643) |
| Chr03_30715958 | Chr03 | C | T | 1.58982E-11 | 10.79865 | intergenic | ene:MD03G1222600(dist=17742),gene:MD03G1222700(dist=22641) |
| Chr03_30715964 | Chr03 | A | C | 1.58982E-11 | 10.79865 | intergenic | ene:MD03G1222600(dist=17748),gene:MD03G1222700(dist=22635) |
| Chr03_30715973 | Chr03 | A | G | 3.85387E-11 | 10.4141 | intergenic | ene:MD03G1222600(dist=17757),gene:MD03G1222700(dist=22626) |
| Chr03_30715978 | Chr03 | G | A | 3.85387E-11 | 10.4141 | intergenic | ene:MD03G1222600(dist=17762),gene:MD03G1222700(dist=22621) |
| Chr03_30715992 | Chr03 | C | T | 2.48693E-12 | 11.60434 | intergenic | ene:MD03G1222600(dist=17776),gene:MD03G1222700(dist=22607) |
| Chr03_30716009 | Chr03 | C | T | 7.091E-13 | 12.14929 | intergenic | ene:MD03G1222600(dist=17793),gene:MD03G1222700(dist=22590) |
| Chr03_30716026 | Chr03 | T | C | 1.95E-15 | 14.71103 | intergenic | ene:MD03G1222600(dist=17810),gene:MD03G1222700(dist=22573) |
| Chr03_30716083 | Chr03 | T | C | 3.3405E-08 | 7.476189 | intergenic | ene:MD03G1222600(dist=17867),gene:MD03G1222700(dist=22516) |
| Chr03_30716084 | Chr03 | T | C | 3.3405E-08 | 7.476189 | intergenic | ene:MD03G1222600(dist=17868),gene:MD03G1222700(dist=22515) |
| Chr03_30716091 | Chr03 | A | G | 6.82589E-12 | 11.16584 | intergenic | ene:MD03G1222600(dist=17875),gene:MD03G1222700(dist=22508) |
| Chr03_30716096 | Chr03 | G | T | 6.82589E-12 | 11.16584 | intergenic | ene:MD03G1222600(dist=17880),gene:MD03G1222700(dist=22503) |
| Chr03_30716097 | Chr03 | A | G | 6.82589E-12 | 11.16584 | intergenic | ene:MD03G1222600(dist=17881),gene:MD03G1222700(dist=22502) |
| Chr03_30716112 | Chr03 | A | G | 1.20606E-10 | 9.918631 | intergenic | ene:MD03G1222600(dist=17896),gene:MD03G1222700(dist=22487) |
| Chr03_30716136 | Chr03 | C | T | 1.38532E-11 | 10.85845 | intergenic | ene:MD03G1222600(dist=17920),gene:MD03G1222700(dist=22463) |
| Chr03_30716141 | Chr03 | A | G | 1.38532E-11 | 10.85845 | intergenic | ene:MD03G1222600(dist=17925),gene:MD03G1222700(dist=22458) |
| Chr03_30716151 | Chr03 | C | T | 2.67042E-11 | 10.57342 | intergenic | ene:MD03G1222600(dist=17935),gene:MD03G1222700(dist=22448) |
| Chr03_30716174 | Chr03 | C | T | 1.51149E-10 | 9.820594 | intergenic | ene:MD03G1222600(dist=17958),gene:MD03G1222700(dist=22425) |
| Chr03_30716196 | Chr03 | T | G | 1.07856E-11 | 10.96715 | intergenic | ene:MD03G1222600(dist=17980),gene:MD03G1222700(dist=22403) |
| Chr03_30716276 | Chr03 | T | C | 6.61247E-09 | 8.179636 | intergenic | ene:MD03G1222600(dist=18060),gene:MD03G1222700(dist=22323) |
| Chr03_30716277 | Chr03 | C | T | 5.14385E-09 | 8.288712 | intergenic | ene:MD03G1222600(dist=18061),gene:MD03G1222700(dist=22322) |
| Chr03_30716285 | Chr03 | T | A | 2.86698E-08 | 7.542575 | intergenic | ene:MD03G1222600(dist=18069),gene:MD03G1222700(dist=22314) |
| Chr03_30716289 | Chr03 | G | A | 4.88353E-08 | 7.311267 | intergenic | ene:MD03G1222600(dist=18073),gene:MD03G1222700(dist=22310) |
| Chr03_30716349 | Chr03 | G | A | 8.73895E-09 | 8.058541 | intergenic | ene:MD03G1222600(dist=18133),gene:MD03G1222700(dist=22250) |
| Chr03_30716370 | Chr03 | A | G | 8.73895E-09 | 8.058541 | intergenic | ene:MD03G1222600(dist=18154),gene:MD03G1222700(dist=22229) |
| Chr03_30716384 | Chr03 | G | A | 3.19471E-09 | 8.495568 | intergenic | ene:MD03G1222600(dist=18168),gene:MD03G1222700(dist=22215) |
| Chr03_30716403 | Chr03 | C | T | 3.58604E-09 | 8.445385 | intergenic | ene:MD03G1222600(dist=18187),gene:MD03G1222700(dist=22196) |
| Chr03_30716964 | Chr03 | C | T | 1.46466E-10 | 9.834264 | intergenic | ene:MD03G1222600(dist=18748),gene:MD03G1222700(dist=21635) |
| Chr03_30716971 | Chr03 | A | G | 1.46466E-10 | 9.834264 | intergenic | ene:MD03G1222600(dist=18755),gene:MD03G1222700(dist=21628) |
| Chr03_30716987 | Chr03 | C | T | 8.3683E-12 | 11.07736 | intergenic | ene:MD03G1222600(dist=18771),gene:MD03G1222700(dist=21612) |
| Chr03_30716997 | Chr03 | A | T | 8.3683E-12 | 11.07736 | intergenic | ene:MD03G1222600(dist=18781),gene:MD03G1222700(dist=21602) |
| Chr03_30717009 | Chr03 | A | C | 8.3683E-12 | 11.07736 | intergenic | ene:MD03G1222600(dist=18793),gene:MD03G1222700(dist=21590) |
| Chr03_30717844 | Chr03 | T | A | 2.92313E-08 | 7.534152 | intergenic | ene:MD03G1222600(dist=19628),gene:MD03G1222700(dist=20755) |
| Chr03_30717878 | Chr03 | A | G | 1.49518E-08 | 7.825307 | intergenic | ene:MD03G1222600(dist=19662),gene:MD03G1222700(dist=20721) |
| Chr03_30717899 | Chr03 | C | T | 7.16384E-08 | 7.144854 | intergenic | ene:MD03G1222600(dist=19683),gene:MD03G1222700(dist=20700) |
| Chr03_30717910 | Chr03 | A | G | 6.36252E-08 | 7.196371 | intergenic | ene:MD03G1222600(dist=19694),gene:MD03G1222700(dist=20689) |
| Chr03_30717919 | Chr03 | C | T | 4.95495E-08 | 7.30496 | intergenic | ene:MD03G1222600(dist=19703),gene:MD03G1222700(dist=20680) |
| Chr03_30717932 | Chr03 | A | C | 2.1526E-08 | 7.667037 | intergenic | ene:MD03G1222600(dist=19716),gene:MD03G1222700(dist=20667) |
| Chr03_30723627 | Chr03 | T | A | 1.04729E-08 | 7.979935 | intergenic | ene:MD03G1222600(dist=25411),gene:MD03G1222700(dist=14972) |
| Chr03_30723742 | Chr03 | C | T | 3.88343E-08 | 7.410784 | intergenic | ene:MD03G1222600(dist=25526),gene:MD03G1222700(dist=14857) |
| Chr03_30723746 | Chr03 | T | C | 3.88343E-08 | 7.410784 | intergenic | ene:MD03G1222600(dist=25530),gene:MD03G1222700(dist=14853) |
| Chr03_30723813 | Chr03 | T | C | 1.79477E-09 | 8.745992 | intergenic | ene:MD03G1222600(dist=25597),gene:MD03G1222700(dist=14786) |
| Chr03_30724090 | Chr03 | T | A | 2.99901E-10 | 9.523022 | intergenic | ene:MD03G1222600(dist=25874),gene:MD03G1222700(dist=14509) |
| Chr03_30724092 | Chr03 | T | C | 9.35897E-12 | 11.02877 | intergenic | ene:MD03G1222600(dist=25876),gene:MD03G1222700(dist=14507) |
| Chr03_30724107 | Chr03 | A | T | 4.9595E-12 | 11.30456 | intergenic | ene:MD03G1222600(dist=25891),gene:MD03G1222700(dist=14492) |
| Chr03_30724111 | Chr03 | T | A | 1.53257E-11 | 10.81458 | intergenic | ene:MD03G1222600(dist=25895),gene:MD03G1222700(dist=14488) |
| Chr03_30724154 | Chr03 | C | A | 5.33965E-08 | 7.272487 | intergenic | ene:MD03G1222600(dist=25938),gene:MD03G1222700(dist=14445) |
| Chr03_30724193 | Chr03 | G | A | 7.2686E-09 | 8.138549 | intergenic | ene:MD03G1222600(dist=25977),gene:MD03G1222700(dist=14406) |
| Chr03_30724217 | Chr03 | G | A | 5.20726E-08 | 7.283391 | intergenic | ene:MD03G1222600(dist=26001),gene:MD03G1222700(dist=14382) |
| Chr03_30724218 | Chr03 | C | T | 5.20726E-08 | 7.283391 | intergenic | ene:MD03G1222600(dist=26002),gene:MD03G1222700(dist=14381) |
| Chr03_30724239 | Chr03 | A | G | 6.97474E-09 | 8.156472 | intergenic | ene:MD03G1222600(dist=26023),gene:MD03G1222700(dist=14360) |
| Chr03_30724248 | Chr03 | G | A | 5.72557E-09 | 8.242181 | intergenic | ene:MD03G1222600(dist=26032),gene:MD03G1222700(dist=14351) |
| Chr03_30724260 | Chr03 | T | C | 5.72557E-09 | 8.242181 | intergenic | ene:MD03G1222600(dist=26044),gene:MD03G1222700(dist=14339) |
| Chr03_30724262 | Chr03 | T | C | 5.72557E-09 | 8.242181 | intergenic | ene:MD03G1222600(dist=26046),gene:MD03G1222700(dist=14337) |
| Chr03_30724277 | Chr03 | T | C | 3.87549E-08 | 7.411673 | intergenic | ene:MD03G1222600(dist=26061),gene:MD03G1222700(dist=14322) |
| Chr03_30724296 | Chr03 | C | T | 1.61245E-08 | 7.792514 | intergenic | ene:MD03G1222600(dist=26080),gene:MD03G1222700(dist=14303) |
| Chr03_30724300 | Chr03 | A | G | 1.61245E-08 | 7.792514 | intergenic | ene:MD03G1222600(dist=26084),gene:MD03G1222700(dist=14299) |
| Chr03_30724335 | Chr03 | C | T | 2.70014E-09 | 8.568613 | intergenic | ene:MD03G1222600(dist=26119),gene:MD03G1222700(dist=14264) |
| Chr03_30724762 | Chr03 | C | T | 2.36521E-08 | 7.62613 | intergenic | ene:MD03G1222600(dist=26546),gene:MD03G1222700(dist=13837) |
| Chr03_30724763 | Chr03 | C | T | 1.49951E-08 | 7.824052 | intergenic | ene:MD03G1222600(dist=26547),gene:MD03G1222700(dist=13836) |
| Chr03_30724770 | Chr03 | C | T | 8.19364E-09 | 8.086523 | intergenic | ene:MD03G1222600(dist=26554),gene:MD03G1222700(dist=13829) |
| Chr03_30724774 | Chr03 | C | T | 1.45732E-08 | 7.836446 | intergenic | ene:MD03G1222600(dist=26558),gene:MD03G1222700(dist=13825) |
| Chr03_30724786 | Chr03 | C | T | 1.12787E-08 | 7.947742 | intergenic | ene:MD03G1222600(dist=26570),gene:MD03G1222700(dist=13813) |
| Chr03_30724815 | Chr03 | C | T | 9.63592E-09 | 8.016107 | intergenic | ene:MD03G1222600(dist=26599),gene:MD03G1222700(dist=13784) |
| Chr03_30724884 | Chr03 | A | G | 6.11355E-09 | 8.213707 | intergenic | ene:MD03G1222600(dist=26668),gene:MD03G1222700(dist=13715) |
| Chr03_30724905 | Chr03 | A | T | 1.28406E-08 | 7.891416 | intergenic | ene:MD03G1222600(dist=26689),gene:MD03G1222700(dist=13694) |
| Chr03_30725005 | Chr03 | A | G | 4.26695E-10 | 9.369883 | intergenic | ene:MD03G1222600(dist=26789),gene:MD03G1222700(dist=13594) |
| Chr03_30725012 | Chr03 | A | C | 2.52188E-09 | 8.598276 | intergenic | ene:MD03G1222600(dist=26796),gene:MD03G1222700(dist=13587) |

| Chr03_30725181 | Chr03 | C | T | 7.35045E-09 | 8.133686 | intergenic | ene:MD03G1222600(dist=26965),gene:MD03G1222700(dist=13418) | |
| --- | --- | --- | --- | --- | --- | --- | --- | --- |
| Chr03_30725400 | Chr03 | C | A | 5.33646E-11 | 10.27275 | intergenic | ene:MD03G1222600(dist=27184),gene:MD03G1222700(dist=13199) | |
| Chr03_30725793 | Chr03 | A | C | 1.06941E-08 | 7.970857 | intergenic | ene:MD03G1222600(dist=27577),gene:MD03G1222700(dist=12806) | |
| Chr03_30725800 | Chr03 | T | G | 1.06941E-08 | 7.970857 | intergenic | ene:MD03G1222600(dist=27584),gene:MD03G1222700(dist=12799) | |
| Chr03_30725807 | Chr03 | T | C | 1.06941E-08 | 7.970857 | intergenic | ene:MD03G1222600(dist=27591),gene:MD03G1222700(dist=12792) | |
| Chr03_30726604 | Chr03 | A | G | 3.46501E-09 | 8.460295 | intergenic | ene:MD03G1222600(dist=28388),gene:MD03G1222700(dist=11995) | |
| Chr03_30726700 | Chr03 | T | G | 8.36575E-09 | 8.077495 | intergenic | ene:MD03G1222600(dist=28484),gene:MD03G1222700(dist=11899) | |
| Chr03_30726708 | Chr03 | G | A | 8.36575E-09 | 8.077495 | intergenic | ene:MD03G1222600(dist=28492),gene:MD03G1222700(dist=11891) | |
| Chr03_30726790 | Chr03 | G | A | 1.44662E-09 | 8.839646 | intergenic | ene:MD03G1222600(dist=28574),gene:MD03G1222700(dist=11809) | |
| Chr03_30726812 | Chr03 | G | A | 5.613E-10 | 9.250805 | intergenic | ene:MD03G1222600(dist=28596),gene:MD03G1222700(dist=11787) | |
| Chr03_30726864 | Chr03 | T | G | 2.26872E-09 | 8.644219 | intergenic | ene:MD03G1222600(dist=28648),gene:MD03G1222700(dist=11735) | |
| Chr03_30726866 | Chr03 | T | A | 2.67267E-08 | 7.573054 | intergenic | ene:MD03G1222600(dist=28650),gene:MD03G1222700(dist=11733) | |
| Chr03_30726886 | Chr03 | C | G | 6.65854E-08 | 7.176621 | intergenic | ene:MD03G1222600(dist=28670),gene:MD03G1222700(dist=11713) | |
| Chr03_30726985 | Chr03 | A | T | 3.3844E-11 | 10.47052 | intergenic | ene:MD03G1222600(dist=28769),gene:MD03G1222700(dist=11614) | |
| Chr03_30727012 | Chr03 | C | G | 3.06543E-11 | 10.51351 | intergenic | ene:MD03G1222600(dist=28796),gene:MD03G1222700(dist=11587) | |
| Chr03_30727013 | Chr03 | G | T | 3.06543E-11 | 10.51351 | intergenic | ene:MD03G1222600(dist=28797),gene:MD03G1222700(dist=11586) | |
| Chr03_30727070 | Chr03 | T | C | 9.09802E-10 | 9.041053 | intergenic | ene:MD03G1222600(dist=28854),gene:MD03G1222700(dist=11529) | |
| Chr03_30727081 | Chr03 | C | T | 9.09802E-10 | 9.041053 | intergenic | ene:MD03G1222600(dist=28865),gene:MD03G1222700(dist=11518) | |
| Chr03_30727235 | Chr03 | G | A | 9.46853E-11 | 10.02372 | intergenic | ene:MD03G1222600(dist=29019),gene:MD03G1222700(dist=11364) | |
| Chr03_30727468 | Chr03 | C | T | 1.81259E-10 | 9.7417 | intergenic | ene:MD03G1222600(dist=29252),gene:MD03G1222700(dist=11131) | |
| Chr03_30727486 | Chr03 | A | C | 4.26774E-08 | 7.369802 | intergenic | ene:MD03G1222600(dist=29270),gene:MD03G1222700(dist=11113) | |
| Chr03_30727511 | Chr03 | G | T | 1.39491E-08 | 7.855454 | intergenic | ene:MD03G1222600(dist=29295),gene:MD03G1222700(dist=11088) | |
| Chr03_30727933 | Chr03 | A | G | 1.91198E-08 | 7.718516 | intergenic | ene:MD03G1222600(dist=29717),gene:MD03G1222700(dist=10666) | |
| Chr03_30728013 | Chr03 | G | A | 9.87905E-09 | 8.005285 | intergenic | ene:MD03G1222600(dist=29797),gene:MD03G1222700(dist=10586) | |
| Chr03_30728014 | Chr03 | A | G | 9.87905E-09 | 8.005285 | intergenic | ene:MD03G1222600(dist=29798),gene:MD03G1222700(dist=10585) | |
| Chr03_30728143 | Chr03 | T | C | 4.78603E-09 | 8.320025 | intergenic | ene:MD03G1222600(dist=29927),gene:MD03G1222700(dist=10456) | |
| Chr03_30728176 | Chr03 | C | G | 1.31977E-10 | 9.879503 | intergenic | ene:MD03G1222600(dist=29960),gene:MD03G1222700(dist=10423) | |
| Chr03_30728236 | Chr03 | C | T | 5.89141E-09 | 8.229781 | intergenic | ene:MD03G1222600(dist=30020),gene:MD03G1222700(dist=10363) | |
| Chr03_30728312 | Chr03 | G | A | 7.90374E-12 | 11.10217 | intergenic | ene:MD03G1222600(dist=30096),gene:MD03G1222700(dist=10287) | |
| Chr03_30728528 | Chr03 | G | A | 1.96911E-09 | 8.70573 | intergenic | ene:MD03G1222600(dist=30312),gene:MD03G1222700(dist=10071) | |
| Chr03_30732243 | Chr03 | C | A | 2.03897E-08 | 7.69059 | intergenic | ene:MD03G1222600(dist=34027),gene:MD03G1222700(dist=6356) | |
| Chr03_31078433 | Chr03 | C | T | 1.22308E-08 | 7.912545 | intergenic | ene:MD03G1225000(dist=13180),gene:MD03G1225100(dist=2503) | |
| Chr03_31093384 | Chr03 | C | T | 1.97949E-08 | 7.703446 | upstream;downstream | | gene:MD03G1225300(dist=716);gene:MD03G1225200(dist=433) |

| **Supplementary Table 3. List of potential target genes identified by combining the peaks of data from two biological replicates** | | | | | |
| --- | --- | --- | --- | --- | --- |
| **Chr** | **Start** | **End** | **Chain** | **type** | **Gene and annotation** |
| Chr00 | 1595453 | 1602451 | - | gene | ID=gene:MD00G1012900;Name=MD00G1012900;Note=Glucose-methanol-choline (GMC) oxidoreductase family protein |
| Chr00 | 4046819 | 4061053 | - | gene | ID=gene:MD00G1024800;Name=MD00G1024800;Note=Root hair defective 3 GTP-binding protein (RHD3) |
| Chr00 | 4760819 | 4767160 | - | gene | ID=gene:MD00G1029100;Name=MD00G1029100;Note=aldehyde dehydrogenase 3H1 |
| Chr00 | 8246810 | 8250459 | + | gene | ID=gene:MD00G1045100;Name=MD00G1045100;Note=phospholipase A 2A |
| Chr00 | 10335761 | 10350042 | - | gene | ID=gene:MD00G1055300;Name=MD00G1055300;Note=Citrate synthase family protein |
| Chr00 | 10524980 | 10527411 | + | gene | ID=gene:MD00G1056200;Name=MD00G1056200;Note=auxin response factor 2 |
| Chr00 | 12783543 | 12783635 | + | gene | ID=gene:MD00G1066400;Name=MD00G1066400;anti_codon=GCT;product=tRNA-Ser |
| Chr00 | 16477677 | 16479108 | - | gene | ID=gene:MD00G1082800;Name=MD00G1082800;Note=Photosystem I assembly protein Ycf3 |
| Chr00 | 16477677 | 16479108 | - | gene | ID=gene:MD00G1082800;Name=MD00G1082800;Note=Photosystem I assembly protein Ycf3 |
| Chr00 | 19896941 | 19900735 | + | gene | ID=gene:MD00G1095000;Name=MD00G1095000;Note=allantoinase |
| Chr00 | 19986415 | 19988634 | - | gene | ID=gene:MD00G1096100;Name=MD00G1096100 |
| Chr00 | 26066982 | 26067135 | + | gene | ID=gene:MD00G1121700;Name=MD00G1121700;Note=NADH-Ubiquinone oxidoreductase (complex I) |
| Chr00 | 30561346 | 30561682 | - | gene | ID=gene:MD00G1140100;Name=MD00G1140100;Note=Uncharacterized protein ycf68 |
| Chr00 | 31709400 | 31709475 | + | gene | ID=gene:MD00G1144900;Name=MD00G1144900;anti_codon=TGT;product=tRNA-Thr |
| Chr00 | 35857503 | 35864060 | + | gene | ID=gene:MD00G1161300;Name=MD00G1161300;Note=RNA-binding (RRM/RBD/RNP motifs) family protein |
| Chr00 | 36867357 | 36868944 | - | gene | ID=gene:MD00G1163700;Name=MD00G1163700 |
| Chr00 | 37243999 | 37253705 | - | gene | ID=gene:MD00G1164400;Name=MD00G1164400;Note=lysophosphatidyl acyltransferase 3 |
| Chr00 | 38496455 | 38496546 | + | gene | ID=gene:MD00G1167600;Name=MD00G1167600;anti_codon=GGA;product=tRNA-Ser |
| Chr00 | 39131596 | 39134030 | + | gene | ID=gene:MD00G1169900;Name=MD00G1169900;Note=interferon-related developmental regulator family protein / IFRD protein family |
| Chr00 | 39251974 | 39256807 | - | gene | ID=gene:MD00G1170600;Name=MD00G1170600 |
| Chr00 | 40671202 | 40674296 | - | gene | ID=gene:MD00G1175500;Name=MD00G1175500 |
| Chr00 | 45412676 | 45422800 | - | gene | ID=gene:MD00G1189500;Name=MD00G1189500 |
| Chr00 | 45654149 | 45663384 | - | gene | ID=gene:MD00G1190000;Name=MD00G1190000 |
| Chr00 | 45985640 | 45988008 | + | gene | ID=gene:MD00G1190900;Name=MD00G1190900;Note=beta glucosidase 15 |
| Chr00 | 46325933 | 46326706 | + | gene | ID=gene:MD00G1191200;Name=MD00G1191200 |
| Chr00 | 48263537 | 48264539 | - | gene | ID=gene:MD00G1202200;Name=MD00G1202200;rfam_acc=RF02543;rfam_id=LSU_rRNA_eukarya |
| Chr00 | 48602820 | 48606285 | - | gene | ID=gene:MD00G1203000;Name=MD00G1203000 |
| Chr00 | 48602820 | 48606285 | - | gene | ID=gene:MD00G1203000;Name=MD00G1203000 |
| Chr00 | 49321365 | 49329101 | - | gene | ID=gene:MD00G1204300;Name=MD00G1204300 |
| Chr00 | 50796433 | 50799685 | - | gene | ID=gene:MD00G1208400;Name=MD00G1208400;Note=Family of unknown function (DUF566) |
| Chr00 | 51003185 | 51003307 | - | gene | ID=gene:MD00G1213400;Name=MD00G1213400;Note=8s_rRNA |
| Chr00 | 51003508 | 51003631 | - | gene | ID=gene:MD00G1213500;Name=MD00G1213500;Note=8s_rRNA |
| Chr00 | 52362335 | 52367181 | + | gene | ID=gene:MD00G1221400;Name=MD00G1221400;Note=cinnamate-4-hydroxylase |
| Chr00 | 52362335 | 52367181 | + | gene | ID=gene:MD00G1221400;Name=MD00G1221400;Note=cinnamate-4-hydroxylase |
| Chr01 | 1483976 | 1484198 | - | gene | ID=gene:MD01G1004700;Name=MD01G1004700;Note=Ribosomal protein S7p/S5e family protein |
| Chr01 | 1484444 | 1484518 | - | gene | ID=gene:MD01G1004800;Name=MD01G1004800;rfam_acc=RF00029;rfam_id=Intron_gpII |
| Chr01 | 2918683 | 2918941 | - | gene | ID=gene:MD01G1006600;Name=MD01G1006600;Note=RNA polymerase subunit alpha |
| Chr01 | 6769552 | 6772375 | - | gene | ID=gene:MD01G1014400;Name=MD01G1014400;Note=DNA-directed RNA polymerase family protein |
| Chr01 | 6769552 | 6772375 | - | gene | ID=gene:MD01G1014400;Name=MD01G1014400;Note=DNA-directed RNA polymerase family protein |
| Chr01 | 10947747 | 10954742 | - | gene | ID=gene:MD01G1031200;Name=MD01G1031200;Note=ubiquitin-specific protease 3 |
| Chr01 | 15590838 | 15594189 | + | gene | ID=gene:MD01G1051700;Name=MD01G1051700;Note=beta-galactosidase 8 |
| Chr01 | 16356822 | 16364269 | + | gene | ID=gene:MD01G1059700;Name=MD01G1059700;Note=Protein kinase superfamily protein |
| Chr01 | 19498359 | 19504648 | - | gene | ID=gene:MD01G1086500;Name=MD01G1086500;Note=CDPK-related kinase 3 |
| Chr01 | 21493859 | 21498329 | - | gene | ID=gene:MD01G1102800;Name=MD01G1102800;Note=RNA recognition motif and CCHC-type zinc finger domains containing protein |
| Chr01 | 23347435 | 23351576 | + | gene | ID=gene:MD01G1119700;Name=MD01G1119700;Note=alpha/beta-Hydrolases superfamily protein |
| Chr01 | 23894981 | 23900369 | + | gene | ID=gene:MD01G1127200;Name=MD01G1127200;Note=heat shock protein 70 |
| Chr01 | 25794005 | 25795637 | - | gene | ID=gene:MD01G1149200;Name=MD01G1149200;Note=Ribosomal protein L10 family protein |
| Chr01 | 25801882 | 25809265 | - | gene | ID=gene:MD01G1149300;Name=MD01G1149300;Note=O-methyltransferase family protein |
| Chr01 | 26075537 | 26080367 | + | gene | ID=gene:MD01G1152100;Name=MD01G1152100;Note=Cytochrome P450 superfamily protein |
| Chr01 | 27161571 | 27167605 | - | gene | ID=gene:MD01G1166700;Name=MD01G1166700;Note=expansin A8 |
| Chr01 | 29641947 | 29644649 | - | gene | ID=gene:MD01G1198900;Name=MD01G1198900 |
| Chr01 | 29984107 | 29989047 | + | gene | ID=gene:MD01G1204400;Name=MD01G1204400;Note=cytochrome P450 |
| Chr01 | 31286400 | 31291940 | + | gene | ID=gene:MD01G1220800;Name=MD01G1220800;Note=highly ABA-induced PP2C gene 3 |
| Chr01 | 31286400 | 31291940 | + | gene | ID=gene:MD01G1220800;Name=MD01G1220800;Note=highly ABA-induced PP2C gene 3 |
| Chr01 | 31859336 | 31862626 | - | gene | ID=gene:MD01G1229100;Name=MD01G1229100;Note=O-methyltransferase 1 |
| Chr01 | 32373034 | 32379246 | - | gene | ID=gene:MD01G1236300;Name=MD01G1236300;Note=4-coumarate:CoA ligase 3 |
| Chr02 | 189890 | 195203 | - | gene | ID=gene:MD02G1002200;Name=MD02G1002200 |
| Chr02 | 685670 | 687576 | - | gene | ID=gene:MD02G1010500;Name=MD02G1010500;Note=temperature-induced lipocalin |
| Chr02 | 2467055 | 2469905 | + | gene | ID=gene:MD02G1031700;Name=MD02G1031700 |
| Chr02 | 3229826 | 3232856 | - | gene | ID=gene:MD02G1039800;Name=MD02G1039800;Note=Homeodomain-like superfamily protein |
| Chr02 | 3869976 | 3870583 | - | gene | ID=gene:MD02G1049400;Name=MD02G1049400;Note=acetyl-CoA carboxylase carboxyl transferase subunit beta |
| Chr02 | 3870638 | 3870986 | - | gene | ID=gene:MD02G1049500;Name=MD02G1049500;Note=acetyl-CoA carboxylase carboxyl transferase subunit beta |
| Chr02 | 3871078 | 3872473 | - | gene | ID=gene:MD02G1049600;Name=MD02G1049600;Note=ribulose-bisphosphate carboxylases |
| Chr02 | 4290512 | 4293601 | - | gene | ID=gene:MD02G1052400;Name=MD02G1052400 |
| Chr02 | 5989736 | 5995852 | - | gene | ID=gene:MD02G1075200;Name=MD02G1075200;Note=NB-ARC domain-containing disease resistance protein |
| Chr02 | 6137562 | 6154707 | - | gene | ID=gene:MD02G1078000;Name=MD02G1078000;Note=cyclic nucleotide gated channel 1 |
| Chr02 | 6193303 | 6203492 | - | gene | ID=gene:MD02G1078400;Name=MD02G1078400;Note=cyclic nucleotide gated channel 1 |
| Chr02 | 7142233 | 7145315 | + | gene | ID=gene:MD02G1090000;Name=MD02G1090000;Note=Mob1/phocein family protein |
| Chr02 | 7432036 | 7432104 | - | gene | ID=gene:MD02G1093400;Name=MD02G1093400;rfam_acc=RF00029;rfam_id=Intron_gpII |
| Chr02 | 7841083 | 7853984 | + | gene | ID=gene:MD02G1099000;Name=MD02G1099000;Note=disease resistance protein (TIR-NBS-LRR class) |
| Chr02 | 7841083 | 7853984 | + | gene | ID=gene:MD02G1099000;Name=MD02G1099000;Note=disease resistance protein (TIR-NBS-LRR class) |
| Chr02 | 7880621 | 7880936 | + | gene | ID=gene:MD02G1099400;Name=MD02G1099400;Note=Photosystem I |
| Chr02 | 7997672 | 7999718 | - | gene | ID=gene:MD02G1100400;Name=MD02G1100400;Note=pectin methylesterase 2 |
| Chr02 | 9122133 | 9122812 | + | gene | ID=gene:MD02G1111900;Name=MD02G1111900;Note=disease resistance protein (TIR-NBS-LRR class) |
| Chr02 | 9894502 | 9901698 | + | gene | ID=gene:MD02G1121300;Name=MD02G1121300;Note=Regulator of Vps4 activity in the MVB pathway protein |
| Chr02 | 12586017 | 12590719 | + | gene | ID=gene:MD02G1152300;Name=MD02G1152300 |
| Chr02 | 13024356 | 13025634 | - | gene | ID=gene:MD02G1156600;Name=MD02G1156600;Note=Tetratricopeptide repeat (TPR)-like superfamily protein |
| Chr02 | 16332522 | 16335709 | - | gene | ID=gene:MD02G1181500;Name=MD02G1181500;Note=alpha/beta-Hydrolases superfamily protein |
| Chr02 | 16703728 | 16708235 | - | gene | ID=gene:MD02G1184400;Name=MD02G1184400;Note=BTB and TAZ domain protein 4 |
| Chr02 | 21344546 | 21344762 | + | gene | ID=gene:MD02G1208400;Name=MD02G1208400;Note=Plant mitochondrial ATPase |
| Chr02 | 21697189 | 21701720 | - | gene | ID=gene:MD02G1209900;Name=MD02G1209900;Note=Protein of unknown function |
| Chr02 | 22150068 | 22151965 | - | gene | ID=gene:MD02G1210700;Name=MD02G1210700 |
| Chr02 | 23242446 | 23246046 | - | gene | ID=gene:MD02G1213400;Name=MD02G1213400;Note=RWD domain-containing protein |
| Chr02 | 26216060 | 26227979 | - | gene | ID=gene:MD02G1223800;Name=MD02G1223800 |
| Chr02 | 27666885 | 27669257 | - | gene | ID=gene:MD02G1230900;Name=MD02G1230900;Note=caffeoyl-CoA 3-O-methyltransferase |
| Chr02 | 28783605 | 28795440 | + | gene | ID=gene:MD02G1239400;Name=MD02G1239400;Note=fatty acid reductase 5 |
| Chr02 | 29349491 | 29355130 | + | gene | ID=gene:MD02G1243100;Name=MD02G1243100;Note=lactate/malate dehydrogenase family protein |
| Chr02 | 30566044 | 30569530 | + | gene | ID=gene:MD02G1254100;Name=MD02G1254100 |
| Chr02 | 32056282 | 32061797 | - | gene | ID=gene:MD02G1266500;Name=MD02G1266500;Note=Plant invertase/pectin methylesterase inhibitor superfamily |
| Chr02 | 32149772 | 32154426 | + | gene | ID=gene:MD02G1267200;Name=MD02G1267200;Note=mitogen-activated protein kinase kinase kinase 19 |
| Chr02 | 34101488 | 34105686 | - | gene | ID=gene:MD02G1284600;Name=MD02G1284600;Note=cation/hydrogen exchanger 15 |
| Chr02 | 35844400 | 35846552 | + | gene | ID=gene:MD02G1305300;Name=MD02G1305300;Note=NAD(P)-binding Rossmann-fold superfamily protein |
| Chr02 | 36413636 | 36415326 | + | gene | ID=gene:MD02G1308900;Name=MD02G1308900;Note=Uncharacterized protein |
| Chr02 | 37143632 | 37144591 | + | gene | ID=gene:MD02G1317100;Name=MD02G1317100 |
| Chr03 | 1669339 | 1673844 | - | gene | ID=gene:MD03G1021400;Name=MD03G1021400;Note=beta glucosidase 17 |
| Chr03 | 2348338 | 2353124 | + | gene | ID=gene:MD03G1028800;Name=MD03G1028800;Note=cellulose synthase like E1 |
| Chr03 | 2353715 | 2358216 | - | gene | ID=gene:MD03G1028900;Name=MD03G1028900;Note=cellulose synthase like E1 |
| Chr03 | 2360998 | 2366551 | - | gene | ID=gene:MD03G1029000;Name=MD03G1029000;Note=cellulose synthase like E1 |
| Chr03 | 2716945 | 2720414 | + | gene | ID=gene:MD03G1034100;Name=MD03G1034100 |
| Chr03 | 2959613 | 2964167 | - | gene | ID=gene:MD03G1037400;Name=MD03G1037400;Note=kinesin-like protein 1 |
| Chr03 | 3329554 | 3335617 | - | gene | ID=gene:MD03G1042500;Name=MD03G1042500;Note=actin-related protein 8 |
| Chr03 | 3638667 | 3639776 | - | gene | ID=gene:MD03G1045300;Name=MD03G1045300 |
| Chr03 | 3841682 | 3843392 | + | gene | ID=gene:MD03G1047800;Name=MD03G1047800;Note=Cytochrome b-c1 complex |
| Chr03 | 3946177 | 3954382 | + | gene | ID=gene:MD03G1049200;Name=MD03G1049200;Note=NB-ARC domain-containing disease resistance protein |
| Chr03 | 8627315 | 8629354 | - | gene | ID=gene:MD03G1101200;Name=MD03G1101200;Note=cytochrome P450 |
| Chr03 | 8697171 | 8704320 | - | gene | ID=gene:MD03G1101800;Name=MD03G1101800;Note=Cytochrome P450 superfamily protein |
| Chr03 | 10901574 | 10911999 | + | gene | ID=gene:MD03G1119200;Name=MD03G1119200;Note=SIT4 phosphatase-associated family protein |
| Chr03 | 11022559 | 11022757 | + | gene | ID=gene:MD03G1120200;Name=MD03G1120200;Note=photosystem II reaction center protein F |
| Chr03 | 11351767 | 11354975 | - | gene | ID=gene:MD03G1121900;Name=MD03G1121900;Note=alpha carbonic anhydrase 7 |
| Chr03 | 11448244 | 11455437 | - | gene | ID=gene:MD03G1122400;Name=MD03G1122400;Note=BEL1-like homeodomain 8 |
| Chr03 | 15043312 | 15056383 | - | gene | ID=gene:MD03G1141800;Name=MD03G1141800;Note=Jojoba acyl CoA reductase-related male sterility protein |
| Chr03 | 16061492 | 16067732 | - | gene | ID=gene:MD03G1146300;Name=MD03G1146300;Note=Ankyrin repeat family protein |
| Chr03 | 16087239 | 16087398 | + | gene | ID=gene:MD03G1146700;Name=MD03G1146700;Note=photosystem II reaction center protein K precursor |
| Chr03 | 16726024 | 16735656 | + | gene | ID=gene:MD03G1150300;Name=MD03G1150300 |
| Chr03 | 18484937 | 18488935 | + | gene | ID=gene:MD03G1156900;Name=MD03G1156900;Note=early nodulin-like protein 18 |

| Chr03 | 24188656 | 24195461 | + | gene | ID=gene:MD03G1176400;Name=MD03G1176400;Note=transferases |
| --- | --- | --- | --- | --- | --- |
| Chr03 | 25270999 | 25271077 | + | gene | ID=gene:MD03G1185200;Name=MD03G1185200;rfam_acc=RF00029;rfam_id=Intron_gpII |
| Chr03 | 25338233 | 25341703 | - | gene | ID=gene:MD03G1185900;Name=MD03G1185900;Note=S-locus lectin protein kinase family protein |
| Chr03 | 26860436 | 26864451 | - | gene | ID=gene:MD03G1196100;Name=MD03G1196100;Note=RNAse THREE-like protein 2 |
| Chr03 | 27822092 | 27829107 | + | gene | ID=gene:MD03G1203500;Name=MD03G1203500;Note=EPS15 homology domain 1 |
| Chr03 | 28970762 | 28970836 | - | gene | ID=gene:MD03G1210600;Name=MD03G1210600;anti_codon=GGT;product=tRNA-Thr |
| Chr03 | 32261488 | 32266313 | + | gene | ID=gene:MD03G1237300;Name=MD03G1237300;Note=homogentisate 1 |
| Chr03 | 32261488 | 32266313 | + | gene | ID=gene:MD03G1237300;Name=MD03G1237300;Note=homogentisate 1 |
| Chr03 | 32261488 | 32266313 | + | gene | ID=gene:MD03G1237300;Name=MD03G1237300;Note=homogentisate 1 |
| Chr03 | 32266498 | 32272209 | + | gene | ID=gene:MD03G1237500;Name=MD03G1237500;Note=homogentisate 1 |
| Chr03 | 32266498 | 32272209 | + | gene | ID=gene:MD03G1237500;Name=MD03G1237500;Note=homogentisate 1 |
| Chr03 | 33139534 | 33144665 | - | gene | ID=gene:MD03G1244700;Name=MD03G1244700;Note=gamma-glutamyl hydrolase 2 |
| Chr03 | 33155093 | 33160059 | - | gene | ID=gene:MD03G1244800;Name=MD03G1244800;Note=gamma-glutamyl hydrolase 2 |
| Chr03 | 34665553 | 34666472 | - | gene | ID=gene:MD03G1259800;Name=MD03G1259800 |
| Chr04 | 1147245 | 1149364 | + | gene | ID=gene:MD04G1010400;Name=MD04G1010400;Note=small ubiquitin-like modifier 2 |
| Chr04 | 3808778 | 3818571 | - | gene | ID=gene:MD04G1033900;Name=MD04G1033900;Note=Cyclin/Brf1-like TBP-binding protein |
| Chr04 | 6991521 | 6994123 | + | gene | ID=gene:MD04G1057100;Name=MD04G1057100 |
| Chr04 | 10244676 | 10247610 | + | gene | ID=gene:MD04G1076000;Name=MD04G1076000;Note=C4-dicarboxylate transporter/malic acid transport protein |
| Chr04 | 10411573 | 10422336 | + | gene | ID=gene:MD04G1077000;Name=MD04G1077000;Note=armadillo repeat kinesin 3 |
| Chr04 | 17582634 | 17586393 | + | gene | ID=gene:MD04G1095600;Name=MD04G1095600 |
| Chr04 | 20750514 | 20752424 | - | gene | ID=gene:MD04G1121300;Name=MD04G1121300 |
| Chr04 | 20752566 | 20752640 | + | gene | ID=gene:MD04G1121400;Name=MD04G1121400;anti_codon=TCT;product=tRNA-Arg |
| Chr04 | 20764909 | 20764986 | + | gene | ID=gene:MD04G1121600;Name=MD04G1121600;anti_codon=GCC;product=tRNA-Gly |
| Chr04 | 20765152 | 20765220 | - | gene | ID=gene:MD04G1121700;Name=MD04G1121700;rfam_acc=RF00005;rfam_id=tRNA |
| Chr04 | 20765533 | 20765608 | - | gene | ID=gene:MD04G1121800;Name=MD04G1121800;anti_codon=GAA;product=tRNA-Phe |
| Chr04 | 20765610 | 20772364 | - | gene | ID=gene:MD04G1121900;Name=MD04G1121900;Note=arginine biosynthesis protein ArgJ family |
| Chr04 | 22231345 | 22234376 | + | gene | ID=gene:MD04G1135200;Name=MD04G1135200;Note=NAD(P)-binding Rossmann-fold superfamily protein |
| Chr04 | 22899849 | 22902446 | - | gene | ID=gene:MD04G1140700;Name=MD04G1140700;Note=UDP-glucosyl transferase 85A7 |
| Chr04 | 25331899 | 25336010 | - | gene | ID=gene:MD04G1162200;Name=MD04G1162200;Note=ammonium transporter 2 |
| Chr04 | 26053628 | 26053732 | + | gene | ID=gene:MD04G1169700;Name=MD04G1169700;anti_codon=TGA;product=tRNA-Pseudo |
| Chr04 | 26628318 | 26634026 | - | gene | ID=gene:MD04G1175100;Name=MD04G1175100;Note=vacuolar sorting receptor homolog 1 |
| Chr04 | 27172366 | 27182573 | + | gene | ID=gene:MD04G1181000;Name=MD04G1181000 |
| Chr04 | 27941758 | 27950838 | + | gene | ID=gene:MD04G1188900;Name=MD04G1188900;Note=Sec23/Sec24 protein transport family protein |
| Chr04 | 27958430 | 27959151 | - | gene | ID=gene:MD04G1189100;Name=MD04G1189100;Note=NADH-Ubiquinone/plastoquinone (complex I) protein |
| Chr04 | 28527022 | 28530855 | - | gene | ID=gene:MD04G1197100;Name=MD04G1197100;Note=Protein phosphatase 2C family protein |
| Chr04 | 30188084 | 30190619 | - | gene | ID=gene:MD04G1220800;Name=MD04G1220800;Note=Uncharacterized conserved protein (DUF2358) |
| Chr04 | 30399950 | 30402958 | - | gene | ID=gene:MD04G1223900;Name=MD04G1223900;Note=calmodulin-like 11 |
| Chr04 | 31481013 | 31487896 | + | gene | ID=gene:MD04G1235900;Name=MD04G1235900;Note=Protein of unknown function |
| Chr04 | 32100895 | 32104751 | + | gene | ID=gene:MD04G1245400;Name=MD04G1245400;Note=cytidine/deoxycytidylate deaminase family protein |
| Chr05 | 843091 | 847735 | - | gene | ID=gene:MD05G1001700;Name=MD05G1001700;Note=Exostosin family protein |
| Chr05 | 2448221 | 2451303 | - | gene | ID=gene:MD05G1012200;Name=MD05G1012200;Note=Nodulin MtN3 family protein |
| Chr05 | 3038955 | 3044064 | - | gene | ID=gene:MD05G1017200;Name=MD05G1017200;Note=glycolipid transfer protein 2 |
| Chr05 | 3892538 | 3896016 | - | gene | ID=gene:MD05G1023300;Name=MD05G1023300;Note=Ubiquinol-cytochrome C reductase hinge protein |
| Chr05 | 4049636 | 4054424 | + | gene | ID=gene:MD05G1024800;Name=MD05G1024800;Note=nodulin MtN21 /EamA-like transporter family protein |
| Chr05 | 5298814 | 5304071 | - | gene | ID=gene:MD05G1032400;Name=MD05G1032400;Note=cysteine-rich RLK (RECEPTOR-like protein kinase) 25 |
| Chr05 | 8199964 | 8200059 | + | gene | ID=gene:MD05G1048300;Name=MD05G1048300 |
| Chr05 | 9385128 | 9388096 | + | gene | ID=gene:MD05G1054700;Name=MD05G1054700;Note=3-oxo-5-alpha-steroid 4-dehydrogenase family protein |
| Chr05 | 13304795 | 13307938 | - | gene | ID=gene:MD05G1069000;Name=MD05G1069000;Note=Peroxidase superfamily protein |
| Chr05 | 16806668 | 16811324 | - | gene | ID=gene:MD05G1080300;Name=MD05G1080300;Note=Leucine-rich repeat (LRR) family protein |
| Chr05 | 17728396 | 17731351 | + | gene | ID=gene:MD05G1084700;Name=MD05G1084700;Note=O-acyltransferase (WSD1-like) family protein |
| Chr05 | 19292195 | 19295853 | + | gene | ID=gene:MD05G1091200;Name=MD05G1091200 |
| Chr05 | 21584767 | 21589885 | - | gene | ID=gene:MD05G1103600;Name=MD05G1103600;Note=serine carboxypeptidase-like 18 |
| Chr05 | 22828858 | 22842582 | - | gene | ID=gene:MD05G1111300;Name=MD05G1111300;Note=ARM repeat superfamily protein |
| Chr05 | 25253401 | 25258668 | - | gene | ID=gene:MD05G1128800;Name=MD05G1128800;Note=SKU5 similar 5 |
| Chr05 | 26167967 | 26168255 | - | gene | ID=gene:MD05G1133100;Name=MD05G1133100;Note=Ycf1 protein |
| Chr05 | 26865386 | 26868254 | - | gene | ID=gene:MD05G1138300;Name=MD05G1138300;Note=disease resistance protein (TIR-NBS-LRR class) |
| Chr05 | 27343233 | 27348445 | - | gene | ID=gene:MD05G1142300;Name=MD05G1142300;Note=Acyl-ACP thioesterase |
| Chr05 | 27599192 | 27600509 | - | gene | ID=gene:MD05G1144200;Name=MD05G1144200 |
| Chr05 | 30525087 | 30528051 | + | gene | ID=gene:MD05G1178700;Name=MD05G1178700;Note=Small nuclear ribonucleoprotein family protein |
| Chr05 | 32192339 | 32195288 | - | gene | ID=gene:MD05G1194300;Name=MD05G1194300;Note=GRAS family transcription factor |
| Chr05 | 34178563 | 34182130 | + | gene | ID=gene:MD05G1210400;Name=MD05G1210400;Note=glutathione S-transferase TAU 19 |
| Chr05 | 35298290 | 35301755 | - | gene | ID=gene:MD05G1220200;Name=MD05G1220200;Note=S-locus lectin protein kinase family protein |
| Chr05 | 35364370 | 35366978 | - | gene | ID=gene:MD05G1221100;Name=MD05G1221100;Note=NAC (No Apical Meristem) domain transcriptional regulator superfamily protein |
| Chr05 | 39553170 | 39569185 | - | gene | ID=gene:MD05G1260400;Name=MD05G1260400;Note=Leucine-rich repeat transmembrane protein kinase |
| Chr05 | 39578899 | 39589574 | - | gene | ID=gene:MD05G1260700;Name=MD05G1260700;Note=Leucine-rich repeat transmembrane protein kinase |
| Chr05 | 39636140 | 39644126 | - | gene | ID=gene:MD05G1261100;Name=MD05G1261100;Note=Leucine-rich repeat transmembrane protein kinase |
| Chr05 | 39666399 | 39675137 | + | gene | ID=gene:MD05G1261200;Name=MD05G1261200 |
| Chr05 | 40011059 | 40015621 | + | gene | ID=gene:MD05G1265200;Name=MD05G1265200;Note=WRKY DNA-binding protein 14 |
| Chr05 | 43608333 | 43611706 | - | gene | ID=gene:MD05G1303600;Name=MD05G1303600;Note=GDSL-like Lipase/Acylhydrolase superfamily protein |
| Chr05 | 44914119 | 44917254 | + | gene | ID=gene:MD05G1321600;Name=MD05G1321600;Note=alpha-galactosidase 2 |
| Chr05 | 45608486 | 45613271 | + | gene | ID=gene:MD05G1332600;Name=MD05G1332600;Note=S-locus lectin protein kinase family protein |
| Chr05 | 45669335 | 45670675 | + | gene | ID=gene:MD05G1333400;Name=MD05G1333400;Note=S-locus lectin protein kinase family protein |
| Chr05 | 46278170 | 46281081 | + | gene | ID=gene:MD05G1342400;Name=MD05G1342400;Note=glucose-6-phosphate/phosphate translocator 2 |
| Chr05 | 46597871 | 46606565 | + | gene | ID=gene:MD05G1346800;Name=MD05G1346800;Note=Homeodomain-like superfamily protein |
| Chr05 | 47150137 | 47155528 | + | gene | ID=gene:MD05G1355000;Name=MD05G1355000;Note=EPS15 homology domain 2 |
| Chr06 | 4675576 | 4679818 | - | gene | ID=gene:MD06G1036600;Name=MD06G1036600;Note=NAD(P)-binding Rossmann-fold superfamily protein |
| Chr06 | 14028191 | 14028571 | + | gene | ID=gene:MD06G1065900;Name=MD06G1065900;Note=photosystem II reaction center protein J |
| Chr06 | 17736389 | 17736990 | + | gene | ID=gene:MD06G1072100;Name=MD06G1072100 |
| Chr06 | 20567678 | 20582893 | + | gene | ID=gene:MD06G1084200;Name=MD06G1084200;Note=eukaryotic translation initiation factor 3A |
| Chr06 | 23040295 | 23046358 | - | gene | ID=gene:MD06G1096200;Name=MD06G1096200;Note=Uncharacterized protein family (UPF0016) |
| Chr06 | 23808322 | 23812936 | - | gene | ID=gene:MD06G1101600;Name=MD06G1101600;Note=Aminotransferase-like |
| Chr06 | 26558612 | 26563470 | - | gene | ID=gene:MD06G1124000;Name=MD06G1124000;Note=2-oxoglutarate (2OG) and Fe(II)-dependent oxygenase superfamily protein |
| Chr06 | 27934469 | 27946379 | - | gene | ID=gene:MD06G1134900;Name=MD06G1134900 |
| Chr06 | 27934469 | 27946379 | - | gene | ID=gene:MD06G1134900;Name=MD06G1134900 |
| Chr06 | 28675934 | 28679134 | - | gene | ID=gene:MD06G1143000;Name=MD06G1143000;Note=SWIB/MDM2 domain superfamily protein |
| Chr06 | 31725775 | 31729337 | + | gene | ID=gene:MD06G1177500;Name=MD06G1177500;Note=spermidine synthase 1 |
| Chr06 | 34590427 | 34596372 | + | gene | ID=gene:MD06G1213300;Name=MD06G1213300;Note=cytochrome P450 |
| Chr07 | 304346 | 308552 | + | gene | ID=gene:MD07G1002900;Name=MD07G1002900;Note=DNA-binding bromodomain-containing protein |
| Chr07 | 362570 | 365076 | - | gene | ID=gene:MD07G1003700;Name=MD07G1003700;Note=lipoxygenase 2 |
| Chr07 | 404603 | 412673 | - | gene | ID=gene:MD07G1003900;Name=MD07G1003900;Note=lipoxygenase 2 |
| Chr07 | 698399 | 703792 | + | gene | ID=gene:MD07G1007600;Name=MD07G1007600;Note=UDP-glucosyl transferase 73B3 |
| Chr07 | 1097857 | 1101639 | + | gene | ID=gene:MD07G1012100;Name=MD07G1012100;Note=cytochrome P450 |
| Chr07 | 2077502 | 2093906 | + | gene | ID=gene:MD07G1024900;Name=MD07G1024900;Note=transducin family protein / WD-40 repeat family protein;Alias=MD07G0019400 |
| Chr07 | 3272733 | 3276872 | - | gene | ID=gene:MD07G1038900;Name=MD07G1038900;Note=Major facilitator superfamily protein |
| Chr07 | 3353965 | 3358684 | + | gene | ID=gene:MD07G1039600;Name=MD07G1039600;Note=Major facilitator superfamily protein |
| Chr07 | 6158513 | 6161294 | - | gene | ID=gene:MD07G1065000;Name=MD07G1065000;Note=alpha/beta-Hydrolases superfamily protein |
| Chr07 | 6168967 | 6172815 | - | gene | ID=gene:MD07G1065200;Name=MD07G1065200;Note=alpha/beta-Hydrolases superfamily protein |
| Chr07 | 8014938 | 8018932 | - | gene | ID=gene:MD07G1081900;Name=MD07G1081900;Note=Arabidopsis thaliana protein of unknown function (DUF794) |
| Chr07 | 8046930 | 8048831 | + | gene | ID=gene:MD07G1082100;Name=MD07G1082100;Note=UDP-glucose pyrophosphorylase 2 |
| Chr07 | 9282355 | 9287192 | + | gene | ID=gene:MD07G1088800;Name=MD07G1088800;Note=Protein of unknown function |
| Chr07 | 10145121 | 10148129 | + | gene | ID=gene:MD07G1094500;Name=MD07G1094500 |
| Chr07 | 11670804 | 11675543 | - | gene | ID=gene:MD07G1103100;Name=MD07G1103100;Note=ARP2/3 complex 16 kDa subunit (p16-Arc) |
| Chr07 | 13464103 | 13466839 | + | gene | ID=gene:MD07G1113600;Name=MD07G1113600;Note=expansin B2 |
| Chr07 | 13817937 | 13818553 | + | gene | ID=gene:MD07G1114900;Name=MD07G1114900;rfam_acc=RF02541;rfam_id=LSU_rRNA_bacteria |
| Chr07 | 21034817 | 21040378 | + | gene | ID=gene:MD07G1144600;Name=MD07G1144600;Note=Mitochondrial substrate carrier family protein |
| Chr07 | 21736041 | 21738957 | - | gene | ID=gene:MD07G1148900;Name=MD07G1148900;Note=RING/U-box superfamily protein |
| Chr07 | 25875094 | 25882462 | + | gene | ID=gene:MD07G1179100;Name=MD07G1179100;Note=ureidoglycolate hydrolases |
| Chr07 | 28090852 | 28093190 | - | gene | ID=gene:MD07G1203000;Name=MD07G1203000;Note=sec7 domain-containing protein |
| Chr07 | 28650055 | 28651782 | + | gene | ID=gene:MD07G1207300;Name=MD07G1207300;Note=Encodes a protein involved in salt tolerance |
| Chr07 | 28845824 | 28849297 | + | gene | ID=gene:MD07G1209200;Name=MD07G1209200;Note=UDP-Glycosyltransferase superfamily protein |
| Chr07 | 31109718 | 31110384 | - | gene | ID=gene:MD07G1239700;Name=MD07G1239700;Note=Bifunctional inhibitor/lipid-transfer protein/seed storage 2S albumin superfamily protein |
| Chr07 | 32134528 | 32135359 | + | gene | ID=gene:MD07G1254400;Name=MD07G1254400 |
| Chr07 | 32724434 | 32729961 | + | gene | ID=gene:MD07G1261600;Name=MD07G1261600;Note=Disease resistance protein (TIR-NBS-LRR class) family |
| Chr07 | 35014717 | 35018576 | + | gene | ID=gene:MD07G1289400;Name=MD07G1289400;Note=O-acyltransferase (WSD1-like) family protein |
| Chr07 | 35038424 | 35041872 | + | gene | ID=gene:MD07G1289700;Name=MD07G1289700;Note=CCT motif family protein |
| Chr08 | 44061 | 44136 | + | gene | ID=gene:MD08G1000600;Name=MD08G1000600;anti_codon=GTT;product=tRNA-Asn |
| Chr08 | 317160 | 333837 | - | gene | ID=gene:MD08G1003000;Name=MD08G1003000;Note=Transducin/WD40 repeat-like superfamily protein |
| Chr08 | 342576 | 346324 | + | gene | ID=gene:MD08G1003100;Name=MD08G1003100;Note=Protein of unknown function |
| Chr08 | 655705 | 656071 | - | gene | ID=gene:MD08G1008600;Name=MD08G1008600;Note=RING/U-box superfamily protein |

| Chr08 | 678732 | 684320 | - | gene | ID=gene:MD08G1009200;Name=MD08G1009200;Note=cinnamyl alcohol dehydrogenase 9 |
| --- | --- | --- | --- | --- | --- |
| Chr08 | 747856 | 749731 | - | gene | ID=gene:MD08G1010000;Name=MD08G1010000;Note=SBP (S-ribonuclease binding protein) family protein |
| Chr08 | 1277467 | 1279224 | - | gene | ID=gene:MD08G1016500;Name=MD08G1016500;Note=Protein of unknown function |
| Chr08 | 2147258 | 2150424 | - | gene | ID=gene:MD08G1029600;Name=MD08G1029600;Note=NmrA-like negative transcriptional regulator family protein |
| Chr08 | 3783894 | 3788914 | + | gene | ID=gene:MD08G1048700;Name=MD08G1048700;Note=glutamate receptor 2.7 |
| Chr08 | 4076824 | 4078425 | + | gene | ID=gene:MD08G1051600;Name=MD08G1051600;Note=translocon at the inner envelope membrane of chloroplasts 55-II |
| Chr08 | 4825098 | 4825284 | - | gene | ID=gene:MD08G1060700;Name=MD08G1060700;Note=ATPase |
| Chr08 | 4825406 | 4825494 | - | gene | ID=gene:MD08G1060800;Name=MD08G1060800;rfam_acc=RF00029;rfam_id=Intron_gpII |
| Chr08 | 5032860 | 5042040 | - | gene | ID=gene:MD08G1063900;Name=MD08G1063900 |
| Chr08 | 5732362 | 5746419 | + | gene | ID=gene:MD08G1072000;Name=MD08G1072000;Note=myosin |
| Chr08 | 5889157 | 5892532 | - | gene | ID=gene:MD08G1072800;Name=MD08G1072800;Note=glycosyl hydrolase 9A4 |
| Chr08 | 6895030 | 6897548 | + | gene | ID=gene:MD08G1083100;Name=MD08G1083100 |
| Chr08 | 9561338 | 9565425 | + | gene | ID=gene:MD08G1108600;Name=MD08G1108600;Note=RNApolymerase sigma-subunit C |
| Chr08 | 9889181 | 9890762 | - | gene | ID=gene:MD08G1111900;Name=MD08G1111900 |
| Chr08 | 10128302 | 10132669 | - | gene | ID=gene:MD08G1113800;Name=MD08G1113800;Note=carotenoid cleavage dioxygenase 8 |
| Chr08 | 10425959 | 10430006 | + | gene | ID=gene:MD08G1115100;Name=MD08G1115100;Note=carotenoid cleavage dioxygenase 1 |
| Chr08 | 10729842 | 10731898 | + | gene | ID=gene:MD08G1117200;Name=MD08G1117200;Note=Tetraspanin family protein |
| Chr08 | 11850273 | 11866687 | + | gene | ID=gene:MD08G1126100;Name=MD08G1126100;Note=UDP-N-acetylglucosamine (UAA) transporter family |
| Chr08 | 17189519 | 17189707 | - | gene | ID=gene:MD08G1154800;Name=MD08G1154800;rfam_acc=RF01419;rfam_id=IsrR |
| Chr08 | 17195341 | 17196172 | - | gene | ID=gene:MD08G1154900;Name=MD08G1154900;Note=ribosomal protein L14 |
| Chr08 | 19220728 | 19223905 | - | gene | ID=gene:MD08G1163500;Name=MD08G1163500;Note=ARM repeat superfamily protein |
| Chr08 | 19216985 | 19220701 | - | gene | ID=gene:MD08G1163400;Name=MD08G1163400;Note=ARM repeat superfamily protein |
| Chr08 | 20067784 | 20072887 | + | gene | ID=gene:MD08G1168500;Name=MD08G1168500 |
| Chr08 | 21432772 | 21443326 | - | gene | ID=gene:MD08G1176600;Name=MD08G1176600;Note=Ribosomal protein L7Ae/L30e/S12e/Gadd45 family protein |
| Chr08 | 22686775 | 22689724 | - | gene | ID=gene:MD08G1183000;Name=MD08G1183000;Note=peptidases |
| Chr08 | 23979793 | 23980086 | + | gene | ID=gene:MD08G1190100;Name=MD08G1190100 |
| Chr08 | 26466214 | 26472901 | - | gene | ID=gene:MD08G1204400;Name=MD08G1204400;Note=Rhamnogalacturonate lyase family protein |
| Chr08 | 27872382 | 27882016 | - | gene | ID=gene:MD08G1216000;Name=MD08G1216000;Note=AGAMOUS-like 12 |
| Chr08 | 28672410 | 28672623 | + | gene | ID=gene:MD08G1223200;Name=MD08G1223200;Note=CemA-like proton extrusion protein-related |
| Chr08 | 30646333 | 30649309 | + | gene | ID=gene:MD08G1240200;Name=MD08G1240200;Note=Cox19 family protein (CHCH motif) |
| Chr09 | 3632348 | 3638432 | - | gene | ID=gene:MD09G1054800;Name=MD09G1054800;Note=glycine-rich protein |
| Chr09 | 4453562 | 4455262 | - | gene | ID=gene:MD09G1065400;Name=MD09G1065400;Note=UDP-Glycosyltransferase superfamily protein |
| Chr09 | 8245187 | 8249994 | - | gene | ID=gene:MD09G1110900;Name=MD09G1110900;Note=Ankyrin repeat family protein |
| Chr09 | 8584404 | 8590382 | - | gene | ID=gene:MD09G1113100;Name=MD09G1113100;Note=glycosyl hydrolase 9B8 |
| Chr09 | 9448808 | 9451321 | + | gene | ID=gene:MD09G1122700;Name=MD09G1122700 |
| Chr09 | 10172897 | 10174596 | + | gene | ID=gene:MD09G1132500;Name=MD09G1132500 |
| Chr09 | 11315847 | 11319739 | + | gene | ID=gene:MD09G1145500;Name=MD09G1145500;Note=wall associated kinase-like 2 |
| Chr09 | 11387966 | 11393675 | - | gene | ID=gene:MD09G1146100;Name=MD09G1146100;Note=NAD(P)-linked oxidoreductase superfamily protein |
| Chr09 | 13211208 | 13215313 | - | gene | ID=gene:MD09G1162600;Name=MD09G1162600;Note=23 kDa jasmonate-induced protein |
| Chr09 | 17321754 | 17331850 | - | gene | ID=gene:MD09G1193400;Name=MD09G1193400;Note=Root hair defective 3 GTP-binding protein (RHD3) |
| Chr09 | 19090462 | 19096965 | + | gene | ID=gene:MD09G1203400;Name=MD09G1203400;Note=Pectin lyase-like superfamily protein |
| Chr09 | 19104441 | 19114708 | - | gene | ID=gene:MD09G1203500;Name=MD09G1203500;Note=pleiotropic drug resistance 1 |
| Chr09 | 19174685 | 19179144 | - | gene | ID=gene:MD09G1203800;Name=MD09G1203800;Note=ABC-2 and Plant PDR ABC-type transporter family protein |
| Chr09 | 28299776 | 28300847 | + | gene | ID=gene:MD09G1229200;Name=MD09G1229200;Note=ribosomal protein S2 |
| Chr09 | 29345278 | 29349415 | - | gene | ID=gene:MD09G1233600;Name=MD09G1233600;Note=TRICHOME BIREFRINGENCE-LIKE 43 |
| Chr09 | 29602806 | 29603142 | - | gene | ID=gene:MD09G1235700;Name=MD09G1235700;Note=photosystem II reaction center protein D |
| Chr09 | 32051093 | 32054230 | + | gene | ID=gene:MD09G1250300;Name=MD09G1250300 |
| Chr09 | 32597382 | 32605957 | + | gene | ID=gene:MD09G1254500;Name=MD09G1254500;Note=plastidic GLC translocator |
| Chr09 | 33237694 | 33240226 | - | gene | ID=gene:MD09G1259400;Name=MD09G1259400 |
| Chr09 | 35077930 | 35088248 | - | gene | ID=gene:MD09G1274100;Name=MD09G1274100;Note=ATP binding |
| Chr09 | 37567557 | 37576985 | - | gene | ID=gene:MD09G1293000;Name=MD09G1293000;Note=Transducin family protein / WD-40 repeat family protein |
| Chr10 | 1919364 | 1921890 | - | gene | ID=gene:MD10G1014200;Name=MD10G1014200;Note=alcohol dehydrogenase 1;Alias=MD10G0010900 |
| Chr10 | 4598853 | 4604465 | - | gene | ID=gene:MD10G1035600;Name=MD10G1035600;Note=Pyridoxal phosphate (PLP)-dependent transferases superfamily protein |
| Chr10 | 6595794 | 6595875 | + | gene | ID=gene:MD10G1049700;Name=MD10G1049700;anti_codon=ATC;product=tRNA-Asp |
| Chr10 | 7643760 | 7648582 | + | gene | ID=gene:MD10G1055800;Name=MD10G1055800;Note=dynamin-related protein 3A |
| Chr10 | 13348984 | 13349164 | - | gene | ID=gene:MD10G1089600;Name=MD10G1089600;Note=ribosomal protein S2 |
| Chr10 | 15587329 | 15594868 | + | gene | ID=gene:MD10G1097900;Name=MD10G1097900 |
| Chr10 | 16766401 | 16768863 | + | gene | ID=gene:MD10G1103100;Name=MD10G1103100;Note=Erv1/Alr family protein |
| Chr10 | 16971714 | 16971948 | + | gene | ID=gene:MD10G1104000;Name=MD10G1104000;Note=CemA-like proton extrusion protein-related |
| Chr10 | 17117054 | 17118157 | + | gene | ID=gene:MD10G1104700;Name=MD10G1104700 |
| Chr10 | 17576073 | 17576135 | - | gene | ID=gene:MD10G1107200;Name=MD10G1107200;rfam_acc=RF00005;rfam_id=tRNA |
| Chr10 | 19944336 | 19950079 | - | gene | ID=gene:MD10G1122400;Name=MD10G1122400;Note=Major facilitator superfamily protein |
| Chr10 | 21414016 | 21422513 | - | gene | ID=gene:MD10G1132800;Name=MD10G1132800 |
| Chr10 | 22296545 | 22303467 | + | gene | ID=gene:MD10G1138300;Name=MD10G1138300;Note=MATE efflux family protein |
| Chr10 | 26183003 | 26189311 | - | gene | ID=gene:MD10G1169700;Name=MD10G1169700;Note=NB-ARC domain-containing disease resistance protein |
| Chr10 | 26583570 | 26590774 | - | gene | ID=gene:MD10G1173400;Name=MD10G1173400;Note=global transcription factor group B1 |
| Chr10 | 28764518 | 28768185 | - | gene | ID=gene:MD10G1190500;Name=MD10G1190500;Note=Protein of unknown function |
| Chr10 | 29455189 | 29459892 | + | gene | ID=gene:MD10G1196500;Name=MD10G1196500;Note=glutathione S-transferase TAU 19 |
| Chr10 | 29492440 | 29499355 | - | gene | ID=gene:MD10G1196900;Name=MD10G1196900;Note=glutathione S-transferase TAU 19 |
| Chr10 | 29623581 | 29631938 | + | gene | ID=gene:MD10G1198300;Name=MD10G1198300;Note=photosystem II subunit R |
| Chr10 | 29906851 | 29909700 | - | gene | ID=gene:MD10G1200800;Name=MD10G1200800 |
| Chr10 | 30678017 | 30682119 | + | gene | ID=gene:MD10G1207300;Name=MD10G1207300;Note=cysteine-rich RLK (RECEPTOR-like protein kinase) 10 |
| Chr10 | 31372047 | 31372131 | + | gene | ID=gene:MD10G1215300;Name=MD10G1215300;anti_codon=CAT;product=tRNA-Met |
| Chr10 | 31772153 | 31789342 | + | gene | ID=gene:MD10G1219900;Name=MD10G1219900;Note=ABC transporter family protein |
| Chr10 | 31858837 | 31863419 | + | gene | ID=gene:MD10G1220400;Name=MD10G1220400;Note=ABC transporter family protein |
| Chr10 | 32833605 | 32837507 | + | gene | ID=gene:MD10G1231900;Name=MD10G1231900;Note=20S proteasome alpha subunit E1 |
| Chr10 | 33327388 | 33331968 | + | gene | ID=gene:MD10G1237000;Name=MD10G1237000;Note=TRAM |
| Chr10 | 34164258 | 34170421 | + | gene | ID=gene:MD10G1248500;Name=MD10G1248500;Note=wall-associated kinase 2 |
| Chr10 | 35740568 | 35745562 | + | gene | ID=gene:MD10G1263000;Name=MD10G1263000;Note=Protein of unknown function |
| Chr10 | 36111144 | 36118637 | - | gene | ID=gene:MD10G1268400;Name=MD10G1268400;Note=P-glycoprotein 9 |
| Chr10 | 36237268 | 36239433 | - | gene | ID=gene:MD10G1270300;Name=MD10G1270300;Note=subtilase family protein |
| Chr10 | 36308234 | 36313635 | + | gene | ID=gene:MD10G1270900;Name=MD10G1270900;Note=GDSL-like Lipase/Acylhydrolase superfamily protein |
| Chr10 | 37437986 | 37440258 | + | gene | ID=gene:MD10G1284200;Name=MD10G1284200 |
| Chr10 | 39151883 | 39154599 | + | gene | ID=gene:MD10G1305200;Name=MD10G1305200;Note=Cyclin D6 |
| Chr10 | 40635328 | 40640989 | + | gene | ID=gene:MD10G1326000;Name=MD10G1326000;Note=STRUBBELIG-receptor family 8 |
| Chr10 | 40641313 | 40645040 | + | gene | ID=gene:MD10G1326100;Name=MD10G1326100;Note=O-fucosyltransferase family protein |
| Chr10 | 41190593 | 41193804 | + | gene | ID=gene:MD10G1334500;Name=MD10G1334500;Note=Leucine-rich repeat transmembrane protein kinase |
| Chr10 | 41195169 | 41201868 | + | gene | ID=gene:MD10G1334600;Name=MD10G1334600;Note=Leucine-rich repeat transmembrane protein kinase |
| Chr11 | 351845 | 354820 | - | gene | ID=gene:MD11G1004100;Name=MD11G1004100;Note=Peroxidase superfamily protein |
| Chr11 | 1654495 | 1659712 | - | gene | ID=gene:MD11G1019200;Name=MD11G1019200;Note=UDP-Glycosyltransferase superfamily protein |
| Chr11 | 4076869 | 4083726 | - | gene | ID=gene:MD11G1047000;Name=MD11G1047000;Note=NB-ARC domain-containing disease resistance protein |
| Chr11 | 4307106 | 4313016 | + | gene | ID=gene:MD11G1050400;Name=MD11G1050400;Note=NB-ARC domain-containing disease resistance protein |
| Chr11 | 5210967 | 5213634 | + | gene | ID=gene:MD11G1059900;Name=MD11G1059900;Note=cytochrome P450 |
| Chr11 | 5894308 | 5894614 | + | gene | ID=gene:MD11G1068400;Name=MD11G1068400;Note=YCF9 |
| Chr11 | 5895569 | 5896543 | - | gene | ID=gene:MD11G1068500;Name=MD11G1068500;Note=Photosystem I |
| Chr11 | 7260685 | 7262436 | + | gene | ID=gene:MD11G1086100;Name=MD11G1086100 |
| Chr11 | 7328614 | 7329976 | + | gene | ID=gene:MD11G1087400;Name=MD11G1087400 |
| Chr11 | 8083323 | 8086651 | + | gene | ID=gene:MD11G1097500;Name=MD11G1097500;Note=Haloacid dehalogenase-like hydrolase (HAD) superfamily protein |
| Chr11 | 9903455 | 9911792 | - | gene | ID=gene:MD11G1112000;Name=MD11G1112000 |
| Chr11 | 11284714 | 11290091 | + | gene | ID=gene:MD11G1122500;Name=MD11G1122500;Note=Major facilitator superfamily protein |
| Chr11 | 11941501 | 11953006 | - | gene | ID=gene:MD11G1132000;Name=MD11G1132000;Note=general transcription factor II H2 |
| Chr11 | 12774324 | 12774489 | - | gene | ID=gene:MD11G1138700;Name=MD11G1138700;Note=photosynthetic electron transfer B |
| Chr11 | 14546837 | 14549979 | + | gene | ID=gene:MD11G1152000;Name=MD11G1152000;Note=Serine carboxypeptidase S28 family protein |
| Chr11 | 15123004 | 15127001 | - | gene | ID=gene:MD11G1157100;Name=MD11G1157100;Note=MORN (Membrane Occupation and Recognition Nexus) repeat-containing protein |
| Chr11 | 18400417 | 18403916 | + | gene | ID=gene:MD11G1170400;Name=MD11G1170400;Note=methyl-CPG-binding domain 6 |
| Chr11 | 18771467 | 18775500 | - | gene | ID=gene:MD11G1171600;Name=MD11G1171600;Note=fatty acid reductase 5 |
| Chr11 | 22184070 | 22192798 | + | gene | ID=gene:MD11G1180900;Name=MD11G1180900;Note=C2H2 zinc-finger protein SERRATE (SE) |
| Chr11 | 30214409 | 30221232 | + | gene | ID=gene:MD11G1207500;Name=MD11G1207500;Note=fimbrin 1 |
| Chr11 | 32044128 | 32044202 | - | gene | ID=gene:MD11G1219400;Name=MD11G1219400;anti_codon=TTG;product=tRNA-Gln |
| Chr11 | 37133606 | 37136942 | - | gene | ID=gene:MD11G1258200;Name=MD11G1258200;Note=Basic-leucine zipper (bZIP) transcription factor family protein |
| Chr11 | 38917315 | 38927797 | - | gene | ID=gene:MD11G1272500;Name=MD11G1272500;Note=senescence-related gene 1 |
| Chr11 | 39873627 | 39873870 | - | gene | ID=gene:MD11G1281100;Name=MD11G1281100 |
| Chr11 | 39942997 | 39946350 | - | gene | ID=gene:MD11G1281600;Name=MD11G1281600;Note=cupin family protein |
| Chr11 | 40972410 | 40979560 | - | gene | ID=gene:MD11G1290000;Name=MD11G1290000;Note=nodulin MtN21 /EamA-like transporter family protein |
| Chr11 | 41186617 | 41188974 | + | gene | ID=gene:MD11G1292200;Name=MD11G1292200;Note=Mitochondrial transcription termination factor family protein |
| Chr11 | 41228037 | 41234112 | + | gene | ID=gene:MD11G1292900;Name=MD11G1292900;Note=DNAse I-like superfamily protein |
| Chr11 | 41324372 | 41324689 | - | gene | ID=gene:MD11G1294200;Name=MD11G1294200 |
| Chr11 | 41326104 | 41326254 | - | gene | ID=gene:MD11G1294300;Name=MD11G1294300;Note=ribosomal protein L20 |
| Chr11 | 41327283 | 41328305 | - | gene | ID=gene:MD11G1294400;Name=MD11G1294400;Note=plastid-encoded CLP P |

| Chr11 | 41328327 | 41329405 | + | gene | ID=gene:MD11G1294500;Name=MD11G1294500 |
| --- | --- | --- | --- | --- | --- |
| Chr11 | 41342572 | 41342836 | - | gene | ID=gene:MD11G1294800;Name=MD11G1294800;Note=acetyl-CoA carboxylase carboxyl transferase subunit beta |
| Chr11 | 41342952 | 41344486 | - | gene | ID=gene:MD11G1294900;Name=MD11G1294900;Note=ribulose-bisphosphate carboxylases |
| Chr11 | 41342952 | 41344486 | - | gene | ID=gene:MD11G1294900;Name=MD11G1294900;Note=ribulose-bisphosphate carboxylases |
| Chr11 | 41348193 | 41348268 | - | gene | ID=gene:MD11G1295400;Name=MD11G1295400;anti_codon=CAT;product=tRNA-Met |
| Chr11 | 41348448 | 41350158 | + | gene | ID=gene:MD11G1295500;Name=MD11G1295500 |
| Chr11 | 41350913 | 41351127 | + | gene | ID=gene:MD11G1295600;Name=MD11G1295600;Note=NADH:ubiquinone/plastoquinone oxidoreductase |
| Chr11 | 41351173 | 41351563 | + | gene | ID=gene:MD11G1295700;Name=MD11G1295700;Note=photosystem II reaction center protein G |
| Chr11 | 41888259 | 41889527 | - | gene | ID=gene:MD11G1303500;Name=MD11G1303500 |
| Chr11 | 42780269 | 42786684 | + | gene | ID=gene:MD11G1315900;Name=MD11G1315900;Note=beta-xylosidase 2 |
| Chr12 | 156420 | 157210 | - | gene | ID=gene:MD12G1000600;Name=MD12G1000600 |
| Chr12 | 966982 | 971554 | + | gene | ID=gene:MD12G1009200;Name=MD12G1009200;Note=Protein of unknown function (DUF1637) |
| Chr12 | 5089974 | 5096627 | - | gene | ID=gene:MD12G1045900;Name=MD12G1045900;Note=ABC-2 type transporter family protein |
| Chr12 | 5478687 | 5480352 | + | gene | ID=gene:MD12G1048200;Name=MD12G1048200 |
| Chr12 | 5478687 | 5480352 | + | gene | ID=gene:MD12G1048200;Name=MD12G1048200 |
| Chr12 | 6884631 | 6889366 | - | gene | ID=gene:MD12G1059100;Name=MD12G1059100;Note=DNA-binding bromodomain-containing protein |
| Chr12 | 11878624 | 11881357 | + | gene | ID=gene:MD12G1092600;Name=MD12G1092600;Note=cytochrome P450 |
| Chr12 | 15259916 | 15266153 | - | gene | ID=gene:MD12G1096900;Name=MD12G1096900;Note=Pyruvate kinase family protein |
| Chr12 | 19390075 | 19390187 | - | gene | ID=gene:MD12G1120900;Name=MD12G1120900;rfam_acc=RF00029;rfam_id=Intron_gpII |
| Chr12 | 22273924 | 22278145 | - | gene | ID=gene:MD12G1143700;Name=MD12G1143700;Note=NAD(P)-binding Rossmann-fold superfamily protein |
| Chr12 | 23215483 | 23226811 | + | gene | ID=gene:MD12G1152200;Name=MD12G1152200;Note=Ubiquitin-like superfamily protein |
| Chr12 | 23280865 | 23281013 | + | gene | ID=gene:MD12G1152700;Name=MD12G1152700;rfam_acc=RF02541;rfam_id=LSU_rRNA_bacteria |
| Chr12 | 24419466 | 24426371 | - | gene | ID=gene:MD12G1162900;Name=MD12G1162900;Note=terpene synthase 21 |
| Chr12 | 24641674 | 24644144 | - | gene | ID=gene:MD12G1165400;Name=MD12G1165400;Note=light harvesting complex photosystem II |
| Chr12 | 25053069 | 25062743 | - | gene | ID=gene:MD12G1169100;Name=MD12G1169100;Note=Leucine-rich repeat protein kinase family protein |
| Chr12 | 25457212 | 25467840 | + | gene | ID=gene:MD12G1174400;Name=MD12G1174400;Note=receptor like protein 6 |
| Chr12 | 26030044 | 26040842 | - | gene | ID=gene:MD12G1180400;Name=MD12G1180400;Note=NB-ARC domain-containing disease resistance protein |
| Chr12 | 26893255 | 26895623 | - | gene | ID=gene:MD12G1186800;Name=MD12G1186800;Note=expansin A8 |
| Chr12 | 28788690 | 28790442 | + | gene | ID=gene:MD12G1208400;Name=MD12G1208400 |
| Chr12 | 30114032 | 30124177 | - | gene | ID=gene:MD12G1225600;Name=MD12G1225600;Note=Major facilitator superfamily protein |
| Chr12 | 30114032 | 30124177 | - | gene | ID=gene:MD12G1225600;Name=MD12G1225600;Note=Major facilitator superfamily protein |
| Chr12 | 30150002 | 30166216 | - | gene | ID=gene:MD12G1225900;Name=MD12G1225900;Note=Major facilitator superfamily protein |
| Chr12 | 30252650 | 30254431 | + | gene | ID=gene:MD12G1226800;Name=MD12G1226800;Note=histidine-containing phosphotransmitter 1 |
| Chr12 | 30308629 | 30314219 | - | gene | ID=gene:MD12G1227600;Name=MD12G1227600;Note=Calcium-dependent phospholipid-binding Copine family protein |
| Chr12 | 31126523 | 31126799 | + | gene | ID=gene:MD12G1237700;Name=MD12G1237700;Note=chloroplast ribosomal protein S15 |
| Chr13 | 627656 | 627843 | - | gene | ID=gene:MD13G1009900;Name=MD13G1009900;rfam_acc=RF01419;rfam_id=IsrR |
| Chr13 | 666150 | 668317 | - | gene | ID=gene:MD13G1011000;Name=MD13G1011000;Note=Peroxidase superfamily protein |
| Chr13 | 1529239 | 1534892 | - | gene | ID=gene:MD13G1021200;Name=MD13G1021200;Note=Protein kinase superfamily protein |
| Chr13 | 1675295 | 1679094 | + | gene | ID=gene:MD13G1023300;Name=MD13G1023300;Note=Polyketide cyclase/dehydrase and lipid transport superfamily protein |
| Chr13 | 1928537 | 1932287 | - | gene | ID=gene:MD13G1026600;Name=MD13G1026600;Note=Cupredoxin superfamily protein |
| Chr13 | 2543284 | 2544471 | + | gene | ID=gene:MD13G1036600;Name=MD13G1036600 |
| Chr13 | 3100341 | 3105261 | + | gene | ID=gene:MD13G1044700;Name=MD13G1044700;Note=hAT dimerisation domain-containing protein |
| Chr13 | 3100341 | 3105261 | + | gene | ID=gene:MD13G1044700;Name=MD13G1044700;Note=hAT dimerisation domain-containing protein |
| Chr13 | 3742077 | 3744604 | - | gene | ID=gene:MD13G1052800;Name=MD13G1052800;Note=F-box/RNI-like superfamily protein |
| Chr13 | 4303108 | 4307234 | - | gene | ID=gene:MD13G1061200;Name=MD13G1061200;Note=serine carboxypeptidase-like 20 |
| Chr13 | 4420752 | 4427100 | + | gene | ID=gene:MD13G1064200;Name=MD13G1064200;Note=beta glucosidase 40 |
| Chr13 | 6585026 | 6596595 | + | gene | ID=gene:MD13G1093500;Name=MD13G1093500;Note=Domain of unknown function (DUF220) |
| Chr13 | 6790954 | 6803106 | - | gene | ID=gene:MD13G1096200;Name=MD13G1096200;Note=cysteine-rich RLK (RECEPTOR-like protein kinase) 2 |
| Chr13 | 6833835 | 6838261 | - | gene | ID=gene:MD13G1096600;Name=MD13G1096600;Note=cysteine-rich RLK (RECEPTOR-like protein kinase) 3 |
| Chr13 | 6871523 | 6877575 | + | gene | ID=gene:MD13G1097000;Name=MD13G1097000;Note=Protein of Unknown Function (DUF239) |
| Chr13 | 8799026 | 8803742 | - | gene | ID=gene:MD13G1120000;Name=MD13G1120000 |
| Chr13 | 9661780 | 9668167 | + | gene | ID=gene:MD13G1127800;Name=MD13G1127800;Note=Tetratricopeptide repeat (TPR)-like superfamily protein |
| Chr13 | 10200805 | 10206915 | + | gene | ID=gene:MD13G1133200;Name=MD13G1133200;Note=high affinity K+ transporter 5 |
| Chr13 | 12122408 | 12125844 | + | gene | ID=gene:MD13G1155600;Name=MD13G1155600;Note=HIT zinc finger |
| Chr13 | 13675702 | 13678597 | + | gene | ID=gene:MD13G1169000;Name=MD13G1169000;Note=alanine-tRNA ligases |
| Chr13 | 17030245 | 17034992 | - | gene | ID=gene:MD13G1195700;Name=MD13G1195700;Note=pinoresinol reductase 1 |
| Chr13 | 17842765 | 17843775 | - | gene | ID=gene:MD13G1200600;Name=MD13G1200600;Note=Chlorophyll A-B binding family protein |
| Chr13 | 18993182 | 19006638 | - | gene | ID=gene:MD13G1207400;Name=MD13G1207400;Note=Polyketide cyclase/dehydrase and lipid transport superfamily protein |
| Chr13 | 19587186 | 19587314 | - | gene | ID=gene:MD13G1210500;Name=MD13G1210500;rfam_acc=RF00548;rfam_id=U11 |
| Chr13 | 19785509 | 19789425 |  | gene | ID=gene:MD13G1212100;Name=MD13G1212100;Note=1-aminocyclopropane-1-carboxylate oxidase homolog 1-like |
| Chr13 | 21518791 | 21518871 | - | gene | ID=gene:MD13G1222300;Name=MD13G1222300;anti_codon=ACG;product=tRNA-Arg |
| Chr13 | 21750619 | 21752918 | - | gene | ID=gene:MD13G1223300;Name=MD13G1223300;Note=Adenine nucleotide alpha hydrolases-like superfamily protein |
| Chr13 | 22492073 | 22495997 | - | gene | ID=gene:MD13G1229500;Name=MD13G1229500;Note=Core-2/I-branching beta-1 |
| Chr13 | 22934028 | 22939046 | + | gene | ID=gene:MD13G1231200;Name=MD13G1231200;Note=potassium channel beta subunit 1 |
| Chr13 | 36310763 | 36316803 | - | gene | ID=gene:MD13G1271300;Name=MD13G1271300;Note=ribosomal protein 1 |
| Chr13 | 43203446 | 43206049 | + | gene | ID=gene:MD13G1285100;Name=MD13G1285100;Note=Chalcone and stilbene synthase family protein |
| Chr14 | 3420111 | 3420747 | - | gene | ID=gene:MD14G1037200;Name=MD14G1037200 |
| Chr14 | 4357624 | 4361254 | - | gene | ID=gene:MD14G1045700;Name=MD14G1045700;Note=SPX (SYG1/Pho81/XPR1) domain-containing protein / zinc finger (C3HC4-type RING finger) protein-related |
| Chr14 | 8530507 | 8532527 | + | gene | ID=gene:MD14G1075200;Name=MD14G1075200;Note=heat shock transcription factor B3 |
| Chr14 | 8530507 | 8532527 | + | gene | ID=gene:MD14G1075200;Name=MD14G1075200;Note=heat shock transcription factor B3 |
| Chr14 | 9657675 | 9662014 | + | gene | ID=gene:MD14G1082600;Name=MD14G1082600;Note=dicer-like 2 |
| Chr14 | 10496855 | 10496937 | - | gene | ID=gene:MD14G1085900;Name=MD14G1085900;anti_codon=CAT;product=tRNA-Met |
| Chr14 | 14106702 | 14112445 | - | gene | ID=gene:MD14G1094400;Name=MD14G1094400 |
| Chr14 | 14681300 | 14683101 | + | gene | ID=gene:MD14G1096700;Name=MD14G1096700;Note=cytochrome P450 |
| Chr14 | 21200165 | 21206111 | - | gene | ID=gene:MD14G1133700;Name=MD14G1133700 |
| Chr14 | 24020719 | 24024183 | - | gene | ID=gene:MD14G1147400;Name=MD14G1147400;Note=DCD (Development and Cell Death) domain protein |
| Chr14 | 24162064 | 24166538 | - | gene | ID=gene:MD14G1148900;Name=MD14G1148900;Note=serine carboxypeptidase-like 45 |
| Chr14 | 24776374 | 24776599 | + | gene | ID=gene:MD14G1153600;Name=MD14G1153600;Note=structural constituent of ribosome |
| Chr14 | 25253782 | 25261312 | + | gene | ID=gene:MD14G1158400;Name=MD14G1158400;Note=NRAMP metal ion transporter 6 |
| Chr14 | 25570732 | 25574105 | - | gene | ID=gene:MD14G1161300;Name=MD14G1161300;Note=AMP-dependent synthetase and ligase family protein |
| Chr14 | 28949737 | 28953384 | + | gene | ID=gene:MD14G1200100;Name=MD14G1200100;Note=myb domain protein 94 |
| Chr14 | 29651351 | 29656826 | - | gene | ID=gene:MD14G1211000;Name=MD14G1211000;Note=heat shock protein 101 |
| Chr14 | 31406339 | 31411555 | - | gene | ID=gene:MD14G1234200;Name=MD14G1234200 |
| Chr14 | 31941869 | 31947077 | + | gene | ID=gene:MD14G1240500;Name=MD14G1240500;Note=Homeodomain-like superfamily protein |
| Chr15 | 238036 | 241299 | + | gene | ID=gene:MD15G1003900;Name=MD15G1003900;Note=cold-regulated 47 |
| Chr15 | 1321594 | 1325159 | + | gene | ID=gene:MD15G1022800;Name=MD15G1022800;Note=HXXXD-type acyl-transferase family protein |
| Chr15 | 2443464 | 2447458 | - | gene | ID=gene:MD15G1034600;Name=MD15G1034600;Note=Transmembrane amino acid transporter family protein |
| Chr15 | 2838940 | 2840919 | - | gene | ID=gene:MD15G1040300;Name=MD15G1040300;Note=ATP-dependent caseinolytic (Clp) protease/crotonase family protein |
| Chr15 | 3882462 | 3886258 | - | gene | ID=gene:MD15G1057900;Name=MD15G1057900;Note=Mitochondrial substrate carrier family protein |
| Chr15 | 8400845 | 8409411 | - | gene | ID=gene:MD15G1117800;Name=MD15G1117800;Note=pleiotropic drug resistance 12 |
| Chr15 | 9743183 | 9743429 | + | gene | ID=gene:MD15G1134000;Name=MD15G1134000;Note=ATP synthase subunit C family protein |
| Chr15 | 9745431 | 9749828 | + | gene | ID=gene:MD15G1134100;Name=MD15G1134100;Note=signal recognition particle binding |
| Chr15 | 9950516 | 9959971 | + | gene | ID=gene:MD15G1136300;Name=MD15G1136300;Note=alpha/beta-Hydrolases superfamily protein |
| Chr15 | 10387176 | 10398105 | + | gene | ID=gene:MD15G1140800;Name=MD15G1140800;Note=Protein of unknown function |
| Chr15 | 10542093 | 10551688 | + | gene | ID=gene:MD15G1142600;Name=MD15G1142600;Note=Poly (ADP-ribose) glycohydrolase (PARG) |
| Chr15 | 10920299 | 10923980 | + | gene | ID=gene:MD15G1147000;Name=MD15G1147000;Note=Thioesterase/thiol ester dehydrase-isomerase superfamily protein |
| Chr15 | 11087601 | 11093966 | - | gene | ID=gene:MD15G1150300;Name=MD15G1150300;Note=Cellulose synthase family protein |
| Chr15 | 12957416 | 12962609 | - | gene | ID=gene:MD15G1168600;Name=MD15G1168600;Note=Ubiquitin-like superfamily protein |
| Chr15 | 13518474 | 13525235 | + | gene | ID=gene:MD15G1173200;Name=MD15G1173200;Note=Zinc-binding dehydrogenase family protein |
| Chr15 | 17473182 | 17476688 | + | gene | ID=gene:MD15G1216600;Name=MD15G1216600;Note=Mob1/phocein family protein |
| Chr15 | 18952361 | 18955044 | - | gene | ID=gene:MD15G1232100;Name=MD15G1232100;Note=Ankyrin repeat family protein |
| Chr15 | 19520406 | 19529013 | - | gene | ID=gene:MD15G1237200;Name=MD15G1237200;Note=RNA polymerase II large subunit |
| Chr15 | 20532733 | 20532807 | + | gene | ID=gene:MD15G1247500;Name=MD15G1247500;rfam_acc=RF00005;rfam_id=tRNA |
| Chr15 | 20532975 | 20533045 | - | gene | ID=gene:MD15G1247600;Name=MD15G1247600;anti_codon=GCC;product=tRNA-Gly |
| Chr15 | 23404098 | 23409680 | - | gene | ID=gene:MD15G1270100;Name=MD15G1270100;Note=PQ-loop repeat family protein / transmembrane family protein |
| Chr15 | 24038287 | 24038356 | - | gene | ID=gene:MD15G1274000;Name=MD15G1274000;anti_codon=GTT;product=tRNA-Asn |
| Chr15 | 27103196 | 27104384 | - | gene | ID=gene:MD15G1292600;Name=MD15G1292600;Note=Protein of unknown function |
| Chr15 | 27758354 | 27758935 | + | gene | ID=gene:MD15G1295700;Name=MD15G1295700;rfam_acc=RF00177;rfam_id=SSU_rRNA_bacteria |
| Chr15 | 29180039 | 29182443 | + | gene | ID=gene:MD15G1302200;Name=MD15G1302200;Note=1-amino-cyclopropane-1-carboxylate synthase 8 |
| Chr15 | 29450456 | 29453737 | + | gene | ID=gene:MD15G1303100;Name=MD15G1303100;Note=zinc ion binding |
| Chr15 | 31075483 | 31076207 | - | gene | ID=gene:MD15G1309600;Name=MD15G1309600;Note=Sec-independent periplasmic protein translocase |
| Chr15 | 31233452 | 31237339 | + | gene | ID=gene:MD15G1310700;Name=MD15G1310700;Note=Protein kinase superfamily protein |
| Chr15 | 35399954 | 35407469 | - | gene | ID=gene:MD15G1327400;Name=MD15G1327400;Note=DNAse I-like superfamily protein |
| Chr15 | 37117505 | 37125487 | + | gene | ID=gene:MD15G1333700;Name=MD15G1333700 |
| Chr15 | 40464198 | 40468845 | - | gene | ID=gene:MD15G1345300;Name=MD15G1345300;Note=replication factor C subunit 3 |
| Chr15 | 41327110 | 41332181 | + | gene | ID=gene:MD15G1348800;Name=MD15G1348800;Note=GDSL-like Lipase/Acylhydrolase superfamily protein |
| Chr15 | 41345117 | 41347925 | + | gene | ID=gene:MD15G1348900;Name=MD15G1348900;Note=alpha-fucosidase 1 |
| Chr15 | 44128939 | 44129098 | - | gene | ID=gene:MD15G1364300;Name=MD15G1364300;Note=Chloroplast Ycf2 |
| Chr15 | 46177960 | 46187897 | + | gene | ID=gene:MD15G1377300;Name=MD15G1377300;Note=disease resistance protein (TIR-NBS-LRR class) |

| Chr15 | 47766604 | 47767842 | - | gene | ID=gene:MD15G1386400;Name=MD15G1386400;rfam_acc=RF02543;rfam_id=LSU_rRNA_eukarya |
| --- | --- | --- | --- | --- | --- |
| Chr15 | 48080989 | 48081229 | - | gene | ID=gene:MD15G1388400;Name=MD15G1388400;Note=unfolded protein binding |
| Chr15 | 48485936 | 48493687 | - | gene | ID=gene:MD15G1390400;Name=MD15G1390400;Note=oligopeptide transporter 2 |
| Chr15 | 49591418 | 49593493 | - | gene | ID=gene:MD15G1396400;Name=MD15G1396400;Note=late embryogenesis abundant domain-containing protein / LEA domain-containing protein |
| Chr15 | 51627723 | 51633604 | + | gene | ID=gene:MD15G1415200;Name=MD15G1415200;Note=cellulose synthase like G2 |
| Chr15 | 51627723 | 51633604 | + | gene | ID=gene:MD15G1415200;Name=MD15G1415200;Note=cellulose synthase like G2 |
| Chr15 | 51988669 | 51990943 | - | gene | ID=gene:MD15G1418100;Name=MD15G1418100;Note=plantacyanin |
| Chr15 | 53816619 | 53816954 | + | gene | ID=gene:MD15G1438600;Name=MD15G1438600;rfam_acc=RF01959;rfam_id=SSU_rRNA_archaea |
| Chr15 | 53874438 | 53874643 | + | gene | ID=gene:MD15G1438900;Name=MD15G1438900;Note=maturase K |
| Chr16 | 957084 | 957252 | - | gene | ID=gene:MD16G1012400;Name=MD16G1012400;Note=ribosomal protein S12C |
| Chr16 | 1284234 | 1284504 | - | gene | ID=gene:MD16G1017300;Name=MD16G1017300;Note=photosynthetic electron transfer B |
| Chr16 | 1423525 | 1427485 | + | gene | ID=gene:MD16G1019600;Name=MD16G1019600;Note=Protein of unknown function |
| Chr16 | 1443620 | 1447293 | - | gene | ID=gene:MD16G1020100;Name=MD16G1020100;Note=2-oxoglutarate (2OG) and Fe(II)-dependent oxygenase superfamily protein |
| Chr16 | 1560327 | 1562233 | + | gene | ID=gene:MD16G1021900;Name=MD16G1021900;Note=Plant invertase/pectin methylesterase inhibitor superfamily |
| Chr16 | 1609217 | 1612142 | + | gene | ID=gene:MD16G1022200;Name=MD16G1022200;Note=Plant invertase/pectin methylesterase inhibitor superfamily |
| Chr16 | 1999255 | 2001955 | + | gene | ID=gene:MD16G1028300;Name=MD16G1028300;Note=TPX2 (targeting protein for Xklp2) protein family |
| Chr16 | 2887136 | 2894182 | + | gene | ID=gene:MD16G1040700;Name=MD16G1040700;Note=glutamine-tRNA ligase |
| Chr16 | 4775221 | 4779764 | + | gene | ID=gene:MD16G1068000;Name=MD16G1068000;Note=EXS (ERD1/XPR1/SYG1) family protein |
| Chr16 | 4792507 | 4810064 | + | gene | ID=gene:MD16G1068400;Name=MD16G1068400;Note=histidine kinase 3 |
| Chr16 | 4895512 | 4899433 | - | gene | ID=gene:MD16G1069400;Name=MD16G1069400;Note=exocyst complex component sec15A |
| Chr16 | 5372738 | 5376047 | - | gene | ID=gene:MD16G1076400;Name=MD16G1076400;Note=protein kinase 2B |
| Chr16 | 5438695 | 5444408 | - | gene | ID=gene:MD16G1077700;Name=MD16G1077700;Note=WRKY DNA-binding protein 72 |
| Chr16 | 6949557 | 6950542 | + | gene | ID=gene:MD16G1100100;Name=MD16G1100100;Note=ATPase |
| Chr16 | 6949557 | 6950542 | + | gene | ID=gene:MD16G1100100;Name=MD16G1100100;Note=ATPase |
| Chr16 | 7180040 | 7185152 | - | gene | ID=gene:MD16G1102700;Name=MD16G1102700;Note=MATE efflux family protein |
| Chr16 | 7180040 | 7185152 | - | gene | ID=gene:MD16G1102700;Name=MD16G1102700;Note=MATE efflux family protein |
| Chr16 | 7789752 | 7791177 | - | gene | ID=gene:MD16G1110800;Name=MD16G1110800 |
| Chr16 | 8401567 | 8407573 | + | gene | ID=gene:MD16G1118200;Name=MD16G1118200;Note=caffeoyl-CoA 3-O-methyltransferase |
| Chr16 | 8472258 | 8476744 | + | gene | ID=gene:MD16G1118800;Name=MD16G1118800;Note=SCP1-like small phosphatase 5 |
| Chr16 | 9453792 | 9461355 | - | gene | ID=gene:MD16G1129400;Name=MD16G1129400;Note=Pentatricopeptide repeat (PPR) superfamily protein |
| Chr16 | 11350732 | 11383385 | + | gene | ID=gene:MD16G1145600;Name=MD16G1145600;Note=calcium-dependent lipid-binding family protein |
| Chr16 | 12204543 | 12211349 | - | gene | ID=gene:MD16G1153800;Name=MD16G1153800;Note=phytochrome B |
| Chr16 | 12667476 | 12672350 | + | gene | ID=gene:MD16G1158200;Name=MD16G1158200;Note=acyl-activating enzyme 7 |
| Chr16 | 14070827 | 14079134 | + | gene | ID=gene:MD16G1169300;Name=MD16G1169300;Note=RNA polymerase subunit beta |
| Chr16 | 17772505 | 17777585 | + | gene | ID=gene:MD16G1198200;Name=MD16G1198200;Note=Tetratricopeptide repeat (TPR)-like superfamily protein |
| Chr16 | 19424307 | 19429007 | + | gene | ID=gene:MD16G1207500;Name=MD16G1207500;Note=Protein of unknown function |
| Chr16 | 19839766 | 19840366 | + | gene | ID=gene:MD16G1209700;Name=MD16G1209700 |
| Chr16 | 19847390 | 19847555 | + | gene | ID=gene:MD16G1210100;Name=MD16G1210100;Note=Ycf1 protein |
| Chr16 | 20995727 | 20998705 | + | gene | ID=gene:MD16G1215500;Name=MD16G1215500;Note=2-oxoglutarate (2OG) and Fe(II)-dependent oxygenase superfamily protein |
| Chr16 | 20998749 | 20998910 | - | gene | ID=gene:MD16G1215600;Name=MD16G1215600;rfam_acc=RF01960;rfam_id=SSU_rRNA_eukarya |
| Chr16 | 22560049 | 22566514 | - | gene | ID=gene:MD16G1224300;Name=MD16G1224300;Note=Protein of unknown function (DUF810) |
| Chr16 | 22560049 | 22566514 | - | gene | ID=gene:MD16G1224300;Name=MD16G1224300;Note=Protein of unknown function (DUF810) |
| Chr16 | 22637764 | 22657646 | + | gene | ID=gene:MD16G1224400;Name=MD16G1224400;Note=Cyclopropane-fatty-acyl-phospholipid synthase |
| Chr16 | 22637764 | 22657646 | + | gene | ID=gene:MD16G1224400;Name=MD16G1224400;Note=Cyclopropane-fatty-acyl-phospholipid synthase |
| Chr16 | 27662310 | 27666947 | + | gene | ID=gene:MD16G1247600;Name=MD16G1247600;Note=basic helix-loop-helix (bHLH) DNA-binding superfamily protein |
| Chr16 | 32952498 | 32953476 | - | gene | ID=gene:MD16G1263300;Name=MD16G1263300;Note=ATPase |
| Chr16 | 35367305 | 35368132 | - | gene | ID=gene:MD16G1271600;Name=MD16G1271600;Note=ATP synthase subunit alpha |
| Chr16 | 35392220 | 35392308 | - | gene | ID=gene:MD16G1272000;Name=MD16G1272000;rfam_acc=RF00029;rfam_id=Intron_gpII |
| Chr16 | 35392374 | 35393510 | - | gene | ID=gene:MD16G1272100;Name=MD16G1272100;Note=ATPase |
| Chr16 | 38914227 | 38920342 | + | gene | ID=gene:MD16G1283000;Name=MD16G1283000 |
| Chr16 | 38914227 | 38920342 | + | gene | ID=gene:MD16G1283000;Name=MD16G1283000 |
| Chr16 | 38914227 | 38920342 | + | gene | ID=gene:MD16G1283000;Name=MD16G1283000 |
| Chr17 | 221938 | 229860 | - | gene | ID=gene:MD17G1003100;Name=MD17G1003100;Note=somatic embryogenesis receptor-like kinase 1 |
| Chr17 | 1152447 | 1158430 | - | gene | ID=gene:MD17G1013800;Name=MD17G1013800;Note=Myzus persicae-induced lipase 1 |
| Chr17 | 2061052 | 2065498 | + | gene | ID=gene:MD17G1029100;Name=MD17G1029100;Note=Ankyrin repeat family protein |
| Chr17 | 2593768 | 2596853 | + | gene | ID=gene:MD17G1036500;Name=MD17G1036500;Note=UDP-Glycosyltransferase superfamily protein |
| Chr17 | 4924234 | 4929267 | + | gene | ID=gene:MD17G1060900;Name=MD17G1060900 |
| Chr17 | 6305230 | 6316181 | + | gene | ID=gene:MD17G1076000;Name=MD17G1076000;Note=Ankyrin repeat family protein |
| Chr17 | 6637485 | 6642437 | - | gene | ID=gene:MD17G1080300;Name=MD17G1080300;Note=terpene synthase 04 |
| Chr17 | 7236829 | 7238476 | + | gene | ID=gene:MD17G1087800;Name=MD17G1087800 |
| Chr17 | 8093266 | 8094647 | - | gene | ID=gene:MD17G1095600;Name=MD17G1095600;Note=Transmembrane protein 97 |
| Chr17 | 8453193 | 8456269 | + | gene | ID=gene:MD17G1099500;Name=MD17G1099500;Note=cellulose synthase-like B3 |
| Chr17 | 8562250 | 8569413 | - | gene | ID=gene:MD17G1101100;Name=MD17G1101100;Note=Ankyrin repeat family protein |
| Chr17 | 8805389 | 8808337 | + | gene | ID=gene:MD17G1104000;Name=MD17G1104000;Note=O-Glycosyl hydrolases family 17 protein |
| Chr17 | 8823703 | 8826348 | - | gene | ID=gene:MD17G1104200;Name=MD17G1104200;Note=Ankyrin repeat family protein |
| Chr17 | 9017266 | 9020267 | - | gene | ID=gene:MD17G1105900;Name=MD17G1105900;Note=fatty acid desaturase 5 |
| Chr17 | 10070267 | 10076426 | - | gene | ID=gene:MD17G1117000;Name=MD17G1117000;Note=5'-AMP-activated protein kinase beta-2 subunit protein |
| Chr17 | 10473590 | 10477981 | + | gene | ID=gene:MD17G1120200;Name=MD17G1120200;Note=Cytochrome P450 superfamily protein |
| Chr17 | 10683014 | 10689101 | + | gene | ID=gene:MD17G1123100;Name=MD17G1123100;Note=cation exchanger 5 |
| Chr17 | 10920306 | 10922289 | - | gene | ID=gene:MD17G1125500;Name=MD17G1125500;Note=zinc knuckle (CCHC-type) family protein |
| Chr17 | 12052952 | 12057563 | - | gene | ID=gene:MD17G1134500;Name=MD17G1134500;Note=Pleckstrin homology (PH) domain-containing protein |
| Chr17 | 12999699 | 13002071 | + | gene | ID=gene:MD17G1144200;Name=MD17G1144200 |
| Chr17 | 15162387 | 15164489 | + | gene | ID=gene:MD17G1157300;Name=MD17G1157300;Note=HXXXD-type acyl-transferase family protein |
| Chr17 | 15195925 | 15198915 | + | gene | ID=gene:MD17G1157600;Name=MD17G1157600;Note=HXXXD-type acyl-transferase family protein |
| Chr17 | 16355069 | 16356235 | - | gene | ID=gene:MD17G1164900;Name=MD17G1164900 |
| Chr17 | 20004496 | 20013394 | - | gene | ID=gene:MD17G1176600;Name=MD17G1176600;Note=28s_rRNA |
| Chr17 | 20004496 | 20013394 | - | gene | ID=gene:MD17G1176600;Name=MD17G1176600;Note=28s_rRNA |
| Chr17 | 20579070 | 20583160 | - | gene | ID=gene:MD17G1179300;Name=MD17G1179300;Note=Leucine-rich repeat protein kinase family protein |
| Chr17 | 24612331 | 24616471 | + | gene | ID=gene:MD17G1203500;Name=MD17G1203500;Note=Protein of unknown function |
| Chr17 | 26755967 | 26760225 | + | gene | ID=gene:MD17G1219500;Name=MD17G1219500;Note=cytochrome P450 |
| Chr17 | 29422195 | 29428905 | + | gene | ID=gene:MD17G1245800;Name=MD17G1245800;Note=Terpenoid cyclases family protein |
| Chr17 | 29793636 | 29802662 | - | gene | ID=gene:MD17G1249600;Name=MD17G1249600;Note=Pathogenesis-related thaumatin superfamily protein |
| Chr17 | 32071571 | 32072128 | + | gene | ID=gene:MD17G1260300;Name=MD17G1260300 |
| Chr17 | 33580688 | 33583966 | + | gene | ID=gene:MD17G1274200;Name=MD17G1274200;Note=Disease resistance protein (TIR-NBS-LRR class) family |
| Chr17 | 33879059 | 33880907 | + | gene | ID=gene:MD17G1279000;Name=MD17G1279000;Note=NFU domain protein 1 |
| Chr17 | 34068970 | 34071214 | + | gene | ID=gene:MD17G1280800;Name=MD17G1280800;Note=Protein of unknown function |

| **Supplementary Table 4. Polymorphic bands around the 58-bp InDel detected in all tested accessio** | | | |
| --- | --- | --- | --- |
| **Sample ID** | **Accession name** | **Maturity date** | **size of band** |
| 1 | Fengcun fuji | 131 | 283 bp |
| 2 | Honghua | 139 | 283 bp |
| 3 | Gaojin 5 | 165 | 283 bp |
| 4 | Pingyinduanzhi | 138 | 283 bp |
| 5 | Xingcheng 7-7 | 129 | 283 bp |
| 6 | Fushan 1 | 138 | 283 bp |
| 7 | White Winter Pearmain | 164 | 283 bp |
| 8 | Stark Spur Golden Delicious | 138 | 283 bp |
| 10 | Saint Lawrence | 119 | 225bp/283bp |
| 11 | Xinhong | 120 | 225bp/283bp |
| 13 | 1951-3-1 | 151 | 283 bp |
| 14 | Nero 26 | 130 | 283 bp |
| 15 | Shinano Sweet | 111 | 225bp/283bp |
| 16 | Behene | 136 | 283 bp |
| 17 | Honeycrisp | 116 | 283 bp |
| 18 | Huangfushi | 157 | 283 bp |
| 19 | Zhangjiakouduanzhi | 138 | 283 bp |
| 20 | Sishuiduanzhi | 138 | 283 bp |
| 21 | Kelongxieertouming | 126 | 225bp/283bp |
| 22 | 132 | 138 | 283 bp |
| 23 | Huangjin | 121 | 225bp/283bp |
| 24 | Hardi Spur Delicious | 125 | 283 bp |
| 25 | Kahong | 116 | 225bp/283bp |
| 26 | Meize | NA | 283 bp |
| 27 | Laxton's superb | 151 | 283 bp |
| 28 | Atlas | 119 | 283 bp |
| 29 | Priam | 117 | 283 bp |
| 30 | Zhanxuan 4 | 164 | 283 bp |
| 31 | Prime Gold | 134 | 283 bp |
| 32 | Allington | 134 | 283 bp |
| 33 | Qinguan | 168 | 283 bp |
| 34 | Zhuoai 1 | 164 | 283 bp |
| 35 | Qianqiu | 134 | 283 bp |
| 36 | Cardinal | 134 | 283 bp |
| 37 | II10-15 | 81 | 225 bp |
| 39 | Xingcheng 4-21 | 165 | 283 bp |
| 40 | Huangpi | 159 | 283 bp |
| 41 | Bancroft | 170 | 283 bp |
| 42 | Wealthy | 121 | 283 bp |
| 43 | Changhong | 164 | 283 bp |
| 44 | Fenghuangluanhaitangguo | 120 | 225bp/283bp |
| 45 | Enqi | 120 | 283 bp |
| 46 | Qiheduanjinguan | 139 | 283 bp |
| 47 | Kaisaiweilian | 139 | 283 bp |
| 49 | Clapp | 164 | 283 bp |
| 50 | Fushan 5 | 138 | 283 bp |
| 51 | Miller Sturdy Spur Delicious | 112 | 283 bp |
| 52 | Blushing Golden | 164 | 283 bp |
| 53 | Prima | 116 | 225bp/283bp |
| 54 | Zaoqiangduanzhi | 139 | 283 bp |
| 55 | Youlixiang | 143 | 283 bp |
| 56 | Starkrimson(Holland) | 107 | 283 bp |
| 58 | Kangbingjinguan 51 | 134 | 283 bp |
| 59 | k12 | 116 | 283 bp |
| 60 | Changhong | 149 | 283 bp |
| 61 | Yanfu 1 | 165 | 283 bp |
| 62 | Xingcheng 9-23 | 121 | 283 bp |
| 63 | Guoguang_Ⅰ_11-2_ | 165 | 283 bp |
| 64 | Gaoqiu | NA | 283 bp |
| 65 | Mikilife-1 | 141 | 283 bp |
| 66 | Situonuowei | 78 | 225 bp |
| 67 | Topred fuji | 164 | 283 bp |
| 68 | Ningfeng | 165 | 283 bp |

| 69 | Hongxiezi | 164 | 283 bp |
| --- | --- | --- | --- |
| 70 | Yingjin | 125 | 225bp/283bp |
| 71 | Bo 8 | 134 | 283 bp |
| 72 | Michinoku | 116 | 283 bp |
| 73 | Stark Jumbo | 139 | 283 bp |
| 74 | Aihuang | 164 | 283 bp |
| 75 | Rongguan | 165 | 283 bp |
| 76 | Huamei | 121 | 283 bp |
| 77 | Riskin | 134 | 283 bp |
| 79 | Baumannova reneta | 130 | 283 bp |
| 80 | Royal | 121 | 225 bp |
| 81 | Zhanxuan 16 | 164 | 283 bp |
| 82 | Stark Spur Supreme Red Deliciou | 142 | 283 bp |
| 83 | Xinguang | 164 | 283 bp |
| 84 | Lambourne lord | 121 | 225bp/283bp |
| 85 | Dongchengguan 13 | 134 | 283 bp |
| 86 | Xingcheng 10-18 | 126 | 283 bp |
| 87 | Cloden | 164 | 283 bp |
| 88 | Zaosheng 16 | 113 | 225bp/283bp |
| 89 | Bo 19 | 130 | 283 bp |
| 90 | Shenglihongguan | 121 | 283 bp |
| 93 | Golden Delicious | 163 | 283 bp |
| 94 | Xiongyue 2 | 165 | 283 bp |
| 96 | Shinano RED | NA | 225bp/283bp |
| 97 | May Queen | 134 | 283 bp |
| 98 | Inflancki | 117 | 225bp/283bp |
| 99 | Hesetiaowen | 90 | 225 bp |
| 100 | Jinshayilamu | 134 | 225bp/283bp |
| 101 | Cortland | 85 | 225 bp |
| 102 | Guoqing | 165 | 283 bp |
| 103 | Dabinette | 155 | 283 bp |
| 105 | Xingcheng 27-2 | 165 | 283 bp |
| 106 | Szampion | 165 | 283 bp |
| 107 | Bailuosimalin | 134 | 283 bp |
| 108 | Nanchengaijinguan | 164 | 283 bp |
| 109 | Charles Ross | 134 | 283 bp |
| 110 | American summer pearmain | 117 | 283 bp |
| 112 | Generos | 155 | 283 bp |
| 113 | Meixiang | 146 | 225bp/283bp |
| 114 | Baldwin | 144 | 225bp/283bp |
| 115 | Hadibolaite | 136 | 283 bp |
| 116 | Baldwin | 116 | 225bp/283bp |
| 117 | Red Spur Delicious | 125 | 283 bp |
| 118 | Qindao 1 | 138 | 283 bp |
| 119 | Bianqiangzi 1 | 141 | 283 bp |
| 120 | Miyakiji | 165 | 283 bp |
| 121 | Well Spur Delicious | 138 | 283 bp |
| 123 | De 2 | 126 | 283 bp |
| 124 | Liuyu yabian | 84 | 225 bp |
| 125 | Dajin | 164 | 283 bp |
| 126 | Beifangxinabo | 121 | 283 bp |
| 127 | B-xijinguan | 155 | 283 bp |
| 128 | Bella Vista | 79 | 225 bp |
| 129 | Bismarck | 116 | 225bp/283bp |
| 130 | Juliana | 136 | 283 bp |
| 132 | Qingguang | 164 | 283 bp |
| 133 | Helasang | 134 | 283 bp |
| 134 | Shiai | 164 | 283 bp |
| 135 | Xingcheng 8-2 | 147 | 283 bp |
| 136 | Kelisike | 139 | 283 bp |
| 137 | Weiqinni | 130 | 225bp/283bp |
| 138 | Wangling | 143 | 283 bp |
| 139 | Napoleon | 165 | 225bp/283bp |
| 140 | Seedling Schytte | 134 | 283 bp |
| 142 | Jinshiji | 135 | 283 bp |
| 143 | Qihuyihao 1 | 143 | 283 bp |
| 144 | Yoshkee | NA | 225bp/283bp |

| 145 | Xinjiang 1-3 | 88 | 225 bp |
| --- | --- | --- | --- |
| 146 | Golden Spur Delicious | 165 | 283 bp |
| 147 | Peach | 121 | 225 bp |
| 149 | Delicious | 155 | 283 bp |
| 150 | Aiwq | 155 | 283 bp |
| 151 | Melrose | 134 | 283 bp |
| 152 | Doyle | 113 | 225bp/283bp |
| 153 | Kangtunduanzhi | 134 | 283 bp |
| 154 | xinjiang 327 | 138 | 283 bp |
| 155 | Bensuojinguanyouxi | 134 | 283 bp |
| 157 | Fuqiu | 133 | 283 bp |
| 158 | Doyl | 85 | 225bp/283bp |
| 159 | Starkrimson | 134 | 283 bp |
| 160 | Xinhua 1 | 141 | 225bp/283bp |
| 161 | Xinjiangmianpingguo | 134 | 283 bp |
| 162 | Mikilife-2 | 116 | 283 bp |
| 163 | Lvshuai | NA | 283 bp |
| 165 | Lodi | 164 | 225bp/283bp |
| 166 | Early red gala | 111 | 225bp/283bp |
| 167 | Danding | 117 | 225bp/283bp |
| 168 | Fujing | 165 | 283 bp |
| 169 | Herrnhut | 139 | 283 bp |
| 170 | Shaguo | 103 | 225bp/283bp |
| 172 | Fuhong | 95 | 225bp/283bp |
| 173 | Lobo | 134 | 225bp/283bp |
| 174 | Danxia | 165 | 283 bp |
| 175 | Shidonghaoji | 117 | 225bp/283bp |
| 176 | Early Red Bird | 85 | 225 bp |
| 177 | Simonffy Piros | 116 | 225bp/283bp |
| 178 | Holly | 126 | 283 bp |
| 179 | Jinyu | 134 | 283 bp |
| 180 | Miguo | 157 | 283 bp |
| 181 | Qinglong | 164 | 225bp/283bp |
| 182 | Jacques Lebe-l | NA | 283 bp |
| 183 | GS48 | 126 | 283 bp |
| 184 | Hongxue | 143 | 283 bp |
| 185 | Stark spur | 143 | 283 bp |
| 186 | Zaohongxia | 165 | 283 bp |
| 187 | K10 | 134 | 225bp/283bp |
| 188 | Beijing 0201 | 139 | 283 bp |
| 189 | Lijiangshandingzi | 149 | 283 bp |
| 190 | Megumi | 157 | 225bp/283bp |
| 191 | Judestar | 155 | 283 bp |
| 192 | Fuji_60-22-16 | 139 | 283 bp |
| 193 | Qingsenzaosheng | 116 | 225bp/283bp |
| 194 | Benika | 77 | 225bp/283bp |
| 195 | Judaine | 143 | 283 bp |
| 196 | Norsan | 136 | 283 bp |
| 197 | Red Baron | 76 | 225bp/283bp |
| 198 | Hongfushi TAC | 136 | 283 bp |
| 199 | Xinjiangyepingguo 20-9 | 164 | 283 bp |
| 200 | Qingsen 3 | 136 | 283 bp |
| 201 | Red Reinette Du Canada | 151 | 283 bp |
| 203 | Tianhongyu | 126 | 283 bp |
| 204 | Ⅲ19-13 | 164 | 283 bp |
| 205 | Fujin | 121 | 225 bp |
| 206 | Yanhongmi | 165 | 283 bp |
| 207 | Xinjiang 15-9 | 163 | 283 bp |
| 208 | Rome Beauty | 85 | 225bp/283bp |
| 209 | Huichaoduan | NA | 283 bp |
| 210 | Qingguan | 164 | 283 bp |
| 211 | Calville Rouge | 136 | 283 bp |
| 212 | De 6 | 120 | 283 bp |
| 213 | Jieba | 120 | 225bp/283bp |
| 214 | Xinhongyu | 163 | 283 bp |
| 215 | Hahong | 143 | 283 bp |
| 216 | Meiguihong | 143 | 283 bp |

| 217 | Hebeikangbingjinguan | 130 | 283 bp |
| --- | --- | --- | --- |
| 218 | Mianpingguo | 151 | 283 bp |
| 219 | Zhumaliya | 143 | 225bp/283bp |
| 220 | Lanpengwang | 143 | 283 bp |
| 221 | 60-15-30 | 115 | 283 bp |
| 222 | Xinjiang 30-0 | 117 | 225bp/283bp |
| 223 | Ingram | 164 | 283 bp |
| 224 | Qiujin | 164 | 283 bp |
| 225 | Shanglin | 165 | 283 bp |
| 226 | Koi Hime | 116 | 225bp/283bp |
| 227 | Avrolles | 165 | 283 bp |
| 228 | Qingsenduanzhi Fuji | 165 | 283 bp |
| 229 | Opalescent | 143 | 283 bp |
| 230 | K9 | 85 | 225 bp |
| 231 | Onieffnin | 155 | 283 bp |
| 232 | Xiboliyabaidian | 88 | 225 bp |
| 234 | Longdonghaitang | 165 | 283 bp |
| 235 | Fuji_80-1-70-3 | 165 | 283 bp |
| 236 | Florina | 136 | 283 bp |
| 237 | Chicheng | 120 | 283 bp |
| 238 | Suyisiliebo | 99 | 225bp/283bp |
| 240 | Weixisanye | 165 | 283 bp |
| 241 | Toko | 164 | 283 bp |
| 242 | Tianhuangkui | 98 | 225 bp |
| 243 | Xishuhaitang | 111 | 225 bp |
| 244 | Shengnong 2 | 116 | 225bp/283bp |
| 245 | Dalu 52 | 138 | 225bp/283bp |
| 246 | Xingcheng 1-14 | 112 | 283 bp |
| 247 | Xijinhaitang | 165 | 225bp/283bp |
| 248 | Huangguniang | 85 | 225bp/283bp |
| 249 | Huaguanhaitang | 171 | 283 bp |
| 250 | Zhaaishandingzi | 165 | 283 bp |
| 251 | Maoshanjingzi | 136 | 225bp/283bp |
| 252 | Hongsanye | 165 | 283 bp |
| 253 | Nai 2 | NA | 283 bp |
| 254 | Kuihua | 164 | 283 bp |
| 255 | Gudeboge | 138 | 283 bp |
| 258 | Haitanghua | 165 | 225bp/283bp |
| 259 | 60-4-4 | 103 | 225 bp |
| 263 | Jerseymac | 103 | 283 bp |
| 265 | Xingcheng 21-23 | 143 | 283 bp |
| 268 | Xingcheng 8-8 | 136 | 283 bp |
| 269 | Reinette | 103 | 225bp/283bp |
| 271 | Meiduan 1 | 136 | 225bp/283bp |
| 273 | Fa 5 | 163 | 283 bp |
| 275 | Apple of Commerce | 133 | 283 bp |
| 276 | Mantanghong | 136 | 283 bp |
| 277 | Kosttiq | 165 | 283 bp |
| 279 | Xingcheng 17-10 | 121 | 225bp/283bp |
| 280 | Xingcheng 23-1 | 136 | 283 bp |
| 281 | Jie 9 | 163 | 225bp/283bp |
| 284 | Early Ortley | 136 | 283 bp |
| 286 | Meixiang | 165 | 283 bp |
| 289 | Xingcheng 23-10 | 165 | 283 bp |
| 290 | Benoni | 136 | 225bp/283bp |
| 291 | Qihuyihao 2 | 158 | 283 bp |
| 292 | De 14 | 136 | 283 bp |
| 298 | Early Harvest | 121 | 225bp/283bp |
| 312 | Stark Spur Ultra Red Delicious 1 | 136 | 283 bp |
| 313 | Xingcheng 16-21 | 164 | 283 bp |
| 314 | Fuji_Ⅰ_11-2_ | 165 | 283 bp |
| 315 | Stark Spur Ultra Red Delicious 2 | 136 | 283 bp |
| 316 | Arkansas | 136 | 283 bp |
| 317 | Kogetsu | 136 | 283 bp |
| 318 | 1465 | 164 | 283 bp |
| 320 | Giant Jenifon | 139 | 283 bp |
| 321 | Jinhong | 112 | 225bp/283bp |

| 322 | Jiguan | 164 | 283 bp |
| --- | --- | --- | --- |
| 323 | Hongao | 126 | 283 bp |
| 325 | Qianhetiguoguang | 164 | 283 bp |
| 327 | Xindong | 136 | 283 bp |
| 328 | Ⅱ10-15 | 136 | 283 bp |
| 329 | Xingcheng 10-3 | 151 | 283 bp |
| 330 | Hongge | 110 | 225bp/283bp |
| 331 | Qunfu 1 | 164 | 283 bp |
| 332 | Qiulimeng | 136 | 283 bp |
| 333 | Zhaiteng 2 | 165 | 283 bp |
| 334 | Sakata Tsugaru | 130 | 283 bp |
| 335 | Ningguan | 165 | 283 bp |
| 336 | Alps Otome | 164 | 283 bp |
| 338 | Xinhua 2 | 136 | 283 bp |
| 339 | Yingqiu | 136 | 283 bp |
| 340 | Ningqiu | 121 | 225bp/283bp |
| 341 | Guoguang_60-12-23 | 136 | 283 bp |
| 342 | Akifu 1 | 136 | 283 bp |
| 343 | Xingcheng 25-16 | 165 | 283 bp |
| 344 | Chenango Strawberry | 130 | 283 bp |
| 345 | Shengli | 151 | 283 bp |
| 346 | Spartan | 165 | 283 bp |
| 347 | Xishan 1 | 139 | 283 bp |
| 348 | Fa 3 | 127 | 283 bp |
| 349 | Ben Davis | 164 | 283 bp |
| 350 | Rt du Mans | 164 | 283 bp |
| 351 | Stonetosh | 151 | 283 bp |
| 352 | Changye 1 | 164 | 283 bp |
| 353 | N2 | 136 | 283 bp |
| 354 | Xiaguang | 85 | 283 bp |
| 355 | Xingcheng 19-1 | 151 | 283 bp |
| 357 | Jincui | 130 | 283 bp |
| 358 | Bianqiangzi 2 | 125 | 225bp/283bp |
| 359 | Qiufu 6 | 143 | 283 bp |
| 360 | Pionier | 164 | 283 bp |
| 361 | Pinova | 141 | 283 bp |
| 362 | Wuxiujinguan | 133 | 283 bp |
| 363 | Pacific Rose | 165 | 283 bp |
| 365 | Qiufu 7 | 134 | 283 bp |
| 366 | Rizhiwan-1 | 164 | 283 bp |
| 367 | Cuiyu | 164 | 283 bp |
| 368 | Jie 15 | 136 | 283 bp |
| 369 | Jinhong | 136 | 283 bp |
| 370 | Zaocuilv | 155 | 283 bp |
| 371 | Antalue | 136 | 283 bp |
| 372 | Dailv | 116 | 225bp/283bp |
| 373 | Kufeigan | 165 | 225bp/283bp |
| 374 | Melton | 143 | 283 bp |
| 375 | Magu | 136 | 283 bp |
| 376 | Debaohantang | 169 | 283 bp |
| 377 | Yellow transparent | 81 | 225 bp |
| 378 | Qiufu 39 | 165 | 283 bp |
| 379 | Donghongguo | 153 | 283 bp |
| 380 | GuoguangⅡ_8-19_ | 164 | 283 bp |
| 381 | Liaofu | 85 | 225 bp |
| 382 | Xingcheng 3-2 | 116 | 225bp/283bp |
| 383 | Pingpoguo | 165 | 283 bp |
| 385 | Heiyushandingzi | 167 | 283 bp |
| 388 | Mosiketouming | 126 | 225bp/283bp |
| 389 | Bianyehaitang | 165 | 283 bp |
| 390 | Ruby | 163 | 283 bp |
| 391 | Linzhihaitang | 166 | 283 bp |
| 392 | Zaotaideman | 103 | 225bp/283bp |
| 393 | Lowtosh | 116 | 225bp/283bp |
| 394 | Daihong | 143 | 283 bp |
| 395 | Liberty | 143 | 283 bp |
| 396 | Qingxiang | 155 | 283 bp |

| 397 | Jiabukajinguan | 133 | 283 bp |
| --- | --- | --- | --- |
| 398 | Zhongxing | 116 | 225bp/283bp |
| 399 | Jacques Lebel-2 | 121 | 225bp/283bp |
| 400 | Ningmenghaitang | 177 | 283 bp |
| 401 | Xingcheng 3-19 | 121 | 225bp/283bp |
| 402 | Xingcheng 8-12 | 136 | 283 bp |
| 403 | Sichuanbianye | 136 | 283 bp |
| 404 | Huadao | 112 | 283 bp |
| 405 | Beauty of Bath | 79 | 225 bp |
| 406 | Summerland | 139 | 283 bp |
| 407 | Ruila | 165 | 283 bp |
| 408 | Shalatuoni | 138 | 283 bp |
| 409 | Youyimeigui | 85 | 225 bp |
| 410 | Xifuhaitang | 165 | 225bp/283bp |
| 411 | Macoun | 165 | 283 bp |
| 412 | Deqinhantang | 168 | 283 bp |
| 414 | Fushuai | 139 | 225bp/283bp |
| 416 | Stark Earlibaze | 116 | 225bp/283bp |
| 417 | Shajinhaitang | 165 | 283 bp |
| 418 | Hesefengli | 93 | 225bp/283bp |
| 419 | Xiaojinbianye | 165 | 283 bp |
| 420 | Mengpaisi | 173 | 283 bp |
| 421 | Wushanbianye | 165 | 283 bp |
| 422 | Yajiangbianyehaitang | 165 | 283 bp |
| 423 | Binzi | 132 | 225bp/283bp |
| 424 | Zhaojueshandingzi | 175 | 283 bp |
| 425 | Duohuahaitang | 165 | 225bp/283bp |
| 426 | Smith Cider | 150 | 283 bp |
| 427 | Female Guerrillas | 119 | 225bp/283bp |
| 431 | Houjiadianduanhongxin | 139 | 283 bp |
| 432 | Xingcheng 12-11 | 136 | 283 bp |
| 435 | Freyberg | 163 | 283 bp |
| 438 | Ralls Genet-1 | 163 | 283 bp |
| 442 | Ganhongyu | 121 | 225bp/283bp |
| 444 | Jinguan2 | 144 | 283 bp |
| 445 | Xinjiangyepingguo 5 | NA | 225bp/283bp |
| 448 | 60-17-17 | 121 | 225bp/283bp |
| 458 | 600T | 136 | 283 bp |
| 459 | Budai Domokos | 163 | 283 bp |
| 483 | Drumbo | 164 | 283 bp |
| 498 | Husveti Rosmaring | 164 | 283 bp |
| 499 | Qiufu 1 | 164 | 283 bp |
| 500 | Fengyan | NA | 225 bp |
| 501 | Ganhongyu | 136 | 283 bp |
| 502 | Shengfangfu 3a | 165 | 283 bp |
| 503 | Shengfangfu 1 | 136 | 283 bp |
| 504 | Beifangxinabo | 136 | 283 bp |
| 505 | Cox's Orange Pippin | 136 | 283 bp |
| 506 | Eraly red | 128 | 283 bp |
| 507 | Shizishan 2 | 136 | 283 bp |
| 508 | Hongguoguang | 136 | 283 bp |
| 509 | Sky spur | 164 | 283 bp |
| 510 | 52-6-7 | 141 | 283 bp |
| 511 | Himekami | 143 | 283 bp |
| 512 | Ningguang | 126 | 283 bp |
| 513 | Youyi | 143 | 283 bp |
| 514 | Bo 7 | 136 | 283 bp |
| 515 | Cuihong | NA | 283 bp |
| 516 | Qiufu 5 | 126 | 283 bp |
| 517 | Boiken | 164 | 283 bp |
| 518 | Ruby Red | 165 | 283 bp |
| 519 | Bensuotehao | 164 | 283 bp |
| 521 | Early McIntosh | 91 | 225 bp |
| 522 | Sunggold | 157 | 283 bp |
| 523 | Batul-Alma | 165 | 283 bp |
| 524 | Tsugaru | 120 | 283 bp |
| 525 | Hongxijinqing | 122 | 283 bp |

| 526 | Shizishan | 121 | 225bp/283bp |
| --- | --- | --- | --- |
| 527 | Melba | 112 | 225bp/283bp |
| 528 | Huashuai 1 | 138 | 283 bp |
| 529 | Hanfu | 165 | 283 bp |
| 530 | Black Gilliflower | 121 | 225bp/283bp |
| 531 | Zhongqiu | 136 | 225bp/283bp |
| 532 | Ⅰ8-5 | 138 | 283 bp |
| 533 | Pingzhiguoguang | 163 | 283 bp |
| 534 | Jinguang | 120 | 225bp/283bp |
| 535 | Enweierjinaisheng | 138 | 283 bp |
| 536 | Dongtian | 112 | 225bp/283bp |
| 537 | Sipadun | 139 | 283 bp |
| 538 | Black Ben Davis | 136 | 283 bp |
| 539 | Honey Gold | 120 | 283 bp |
| 540 | Babusijinuo | NA | 283 bp |
| 541 | Red Golden Gala | 143 | 283 bp |
| 542 | Zhanxuan 14 | 164 | 283 bp |
| 543 | Xiushuiguoguang | 130 | 283 bp |
| 544 | Beda | 165 | 283 bp |
| 545 | Rizhiwan-2 | 157 | 283 bp |
| 546 | Scarlet Spur | 116 | 225bp/283bp |
| 548 | Xingcheng 3-20 | 122 | 283 bp |
| 549 | Sanyehaitang | 166 | 283 bp |
| 550 | Michurina | 120 | 283 bp |
| 552 | Sharp Red | 133 | 283 bp |
| 554 | Evelyn | 113 | 225bp/283bp |
| 559 | Jinguan 16-20 | 163 | 283 bp |
| 560 | Qiufu 39 | 163 | 283 bp |
| 562 | Kelia | 116 | 225bp/283bp |
| 563 | Norland | 120 | 283 bp |
| 564 | Changhong 3 | 151 | 283 bp |
| 565 | Chuizhiguoguang | 165 | 283 bp |
| 566 | Sekaiichi | 164 | 283 bp |
| 567 | Chiefeain | 126 | 283 bp |
| 569 | Xingcheng 18-10 | 136 | 283 bp |
| 570 | Starkjambo | 133 | 283 bp |
| 571 | I12-10 | 78 | 225bp/283bp |
| 572 | xinguoguang | 165 | 283 bp |
| 573 | 60-1-59 | 120 | 225bp/283bp |
| 574 | Bo 5 | 163 | 225bp/283bp |
| 575 | De 8 | 165 | 225bp/283bp |
| 576 | Royal Red | 133 | 283 bp |
| 577 | Nagafu 7 | 164 | 225bp/283bp |
| 578 | Judeline | 133 | 283 bp |
| 579 | Xinlimei | 151 | 225bp/283bp |
| 580 | Calville Blanche | 144 | 283 bp |
| 581 | Meltosh | 164 | 283 bp |
| 582 | Xingcheng 18-18 | 110 | 225bp/283bp |
| 601 | Mianpingguo 2 | NA | 225bp/283bp |
| 602 | Xinping 1 | 156 | 283 bp |
| 603 | Kuiping 2 | 157 | 283 bp |
| 604 | Xinjiang 4-10 | NA | 225bp/283bp |
| 605 | Huahong | NA | 225 bp |
| 606 | Nai | NA | 225bp/283bp |
| 607 | Oregon Spur 9 | NA | 283 bp |
| 608 | Xingcheng 0-9 | NA | 225bp/283bp |
| 609 | Xinping 4 | NA | 283 bp |
| 610 | Zhumeihaitang | 136 | 283 bp |
| 620 | Winter Banana | 117 | 283 bp |
| 621 | Geneva Early | 95 | 225 bp |
| 622 | Meiguo 8 | 143 | 283 bp |
| 623 | Maigold | 130 | 283 bp |
| 624 | Daguoshandingzi | 169 | 283 bp |

| **Supplementary table 5. List of primer sequences used in this study.** | | |
| --- | --- | --- |
| **Primer name** | **Primer sequence (5'-3')** | **Purpose** |
| *qMdNAC18.1-F* | CATGTGGGCCACCATCAGAA | qRT-PCR |
| *qMdNAC18.1-R* | CCGTCGGTGCTGCTCATTAT | qRT-PCR |
| *qMdNAC72-F* | TTTGGGAAGACGGCGGAGGAG | qRT-PCR |
| *qMdNAC72-R* | TGAGAGTGTTGCTGGAAGAAGTTGG | qRT-PCR |
| *qMdACS1-F* | ACAGCCTCTCTAAGGATCTTGGTCT | qRT-PCR |
| *qMdACS1-R* | TTTGGTTCTCGGCTATGTAGTTCTT | qRT-PCR |
| *qMdACO1-like-F* | GATACGATGGCTGGAGTCAAAGGC | qRT-PCR |
| *qMdACO1-like-R* | CAGGAATTAGCAGGTTGGGTTGGG | qRT-PCR |
| *qMdMYC2-F* | CTGGACCTACGCCATCTTCT | qRT-PCR |
| *qMdMYC2-R* | CTTGCGGTACTCTTGTTCGG | qRT-PCR |
| *18s rRNA-F* | ACACGGGGAGGTAGTGACAA | qRT-PCR |
| *18s rRNA-R* | CCTCCAATGGATCCTCGTTA | qRT-PCR |

| *MdNAC18.1-pRI101-GFP-F* | ttgatacatatgcccgtcgacATGGAGTGCACCGACTCGTC | constructs in plant transformation |
| --- | --- | --- |
| *MdNAC18.1-pRI101-GFP-R* | tccggtacccccggggtcgacTCCCAAATTGGACTCAGAATACC | constructs in plant transformation |
| *MdNAC72-pRI101-F* | cccgtcgaccccgggggtaccAATGGGTGTGCCGGAAACC | constructs in plant transformation |
| *MdNAC72-pRI101-R* | agagttgttgattcagaattcTTACTGCCAATGCCCGAACC | constructs in plant transformation |
| *MdNAC18.1-TRV-F* | gtgagtaaggttaccgaattcATGGAGTGCACCGACTCGTC | constructs in plant transformation |
| *MdNAC18.1-TRV-R* | cgtgagctcggtaccggatccTAGTGGACGACTAGCTCTTCGTCC | constructs in plant transformation |
| *MdNAC72-TRV-F* | gtgagtaaggttaccgaattcATGGAACGTATTTATCCCCCTTC | constructs in plant transformation |
| *MdNAC72-TRV-R* | cgtgagctcggtaccggatccGAGAGAGAGATTTGAAATTTCTGAAGTT | constructs in plant transformation |
| *MdMYC2-TRV-F* | gtgagtaaggttaccgaattcCATTCGTTGAAGCCCGAATC | constructs in plant transformation |
| *MdMYC2-TRV-R* | cgtgagctcggtaccggatccTTTTTGCTGTTGTTATCCTCAGCG | constructs in plant transformation |

| *MdNAC18.1-HIS-F* | gctgatatcggatccgaattcATGGAGTGCACCGACTCGTC | Protein purification |
| --- | --- | --- |
| *MdNAC18.1-HIS-R* | tgcggccgcaagcttgtcgacTCCCAAATTGGACTCAGAATACC | Protein purification |
| *MdNAC72-HIS-F* | gctgatatcggatccgaattcAATGGGTGTGCCGGAAACC | Protein purification |
| *MdNAC72-HIS-R* | tgcggccgcaagcttgtcgacTTACTGCCAATGCCCGAACC | Protein purification |

| *probe-MdNAC72-F* | Tattgacccacatttgcacgtagccttgtaaacttg | EMSA |
| --- | --- | --- |
| *probe-MdNAC72-R* | CAAGTTTACAAGGCTACGTGCAAATGTGGGTCAATA | EMSA |
| *probe-MutantMdNAC72-F* | tattgacccacattaaaaaaaagccttgtaaacttg | EMSA |
| *probe-MutantMdNAC72-R* | CAAGTTTACAAGGCTtttttttAATGTGGGTCAATA | EMSA |
| *probe-MdACS1-F* | GTACACTTATAATCCCCACGCAAGTTTGTAGGTAAT | EMSA |
| *probe-MdACS1-R* | ATTACCTACAAACTTGCGTGGGGATTATAAGTGTAC | EMSA |
| *probe-MutantMdACS1-F* | GTACACTTATAATAAAAAAACAAGTTTGTAGGTAAT | EMSA |
| *probe-MutantMdACS1-R* | ATTACCTACAAACTTGTTTTTTTATTATAAGTGTAC | EMSA |
| *probe-MdACO1-like-F* | TCAAGTACCATAACTACACGTAACTTGCAAAGTTCC | EMSA |
| *probe-MdACO1-like-F* | GGAACTTTGCAAGTTACGTGTAGTTATGGTACTTGA | EMSA |
| *probe-MutantMdACO1-like-F* | TCAAGTACCATAACAAAAAATAACTTGCAAAGTTCC | EMSA |
| *probe-MutantMdACO1-like-R* | GGAACTTTGCAAGTTATTTTTTGTTATGGTACTTGA | EMSA |
| *probe-MdNAC18.1-F* | GACATACAATGTTCTGATTCGTATTCTAACACTTAA | EMSA |
| *probe-MdNAC18.1-R* | TTAAGTGTTAGAATACGAATCAGAACATTGTATGTC | EMSA |
| *probe-MutantMdNAC18.1-F* | GACATACAATGTTCTGATTCGTATGAGATAACTTAA | EMSA |
| *probe-MutantMdNAC18.1-R* | TTAAGTTATCTCATACGAATCAGAACATTGTATGTC | EMSA |
| *probe-MdMYC2-F* | TCTGGGATCCTGGTTTCACGGGTTTTCGAGGTTTTC | EMSA |
| *probe-MdMYC2-R* | GAAAACCTCGAAAACCCGTGAAACCAGGATCCCAGA | EMSA |
| *probe-MutantMdMYC2-F* | TCTGGGATCCTGGTTTTTTTGGTTTTCGAGGTTTTC | EMSA |
| *probe-MutantMdMYC2-R* | GAAAACCTCGAAAACCAAAAAAACCAGGATCCCAGA | EMSA |

| *MdNAC18.1-AD-F* | ccaaaaaaagagatcgaattcATGGAGTGCACCGACTCGTC | Y1H |
| --- | --- | --- |
| *MdNAC18.1-AD-R* | tcgacggatccccgggaattcCTATCCCAAATTGGACTCAGAATACC | Y1H |
| *MdNAC72-AD-F* | ccaaaaaaagagatcgaattcATGGAACGTATTTATCCCCCTTC | Y1H |
| *MdNAC72-AD-R* | catagatctctgcaggtcgacCTGCCAATGCCCGAACCC | Y1H |
| *proMdACO1-like-phis2-F* | gactcactatagggcgaattcTGGTAGGAGGAGAGCAAAATAAAGT | Y1H |
| *proMdACO1-like-phis2-R* | gattcgcgaacgcgtgagctcCAATTCAGACTCCACGTGTTTTCG | Y1H |
| *proMdACS1-phis2-F* | gactcactatagggcgaattcTTCAATTACCACGGTTACATGTAGATC | Y1H |
| *proMdACS1-phis2-R* | gattcgcgaacgcgtgagctcTTTGCAAATTTTATCTCCAAATTTAAT | Y1H |
| *proMdNAC72-phis2-F* | gactcactatagggcgaattcCCAAACCATTCAGCTGCCG | Y1H |
| *proMdNAC72-phis2-R* | gattcgcgaacgcgtgagctcAACGGTTACAATTATTATAATTTTTAAAAA | Y1H |
| *proMdMYC2-phis2-F* | gactcactatagggcgaattcGCTGAAAAGTCCACATTTACAACTACA | Y1H |
| *proMdMYC2-phis2-R* | gattcgcgaacgcgtgagctcACTCTTCAAACTAGCTTCATCTTACTCG | Y1H |

| *proMdNAC18.1-pGreenII 0800-LUC-F* | gggccccccctcgaggtcgacTCCAATTAATACTCTGAAAGCCAATAA | Dual luciferase reporter assay |
| --- | --- | --- |
| *proMdNAC18.1-pGreenII 0800-LUC-R* | caggaattcgatatcaagcttCCTTGGTTATTGGGTTCGGAG | Dual luciferase reporter assay |
| *proMdACO1-like-pGreenII 0800-LUC-F* | gggccccccctcgaggtcgacCGCGTGGGTCCACGAGCT | Dual luciferase reporter assay |
| *proMdACO1-like-pGreenII 0800-LUC-R* | caggaattcgatatcaagcttGAACTTTTCAATAGCAAAACTTGATCA | Dual luciferase reporter assay |
| *proMdACS1-pGreenII 0800-LUC-F* | gtcgacggtatcgataagcttAATTGTAGCAGAGATTGTAACCCTAAA | Dual luciferase reporter assay |
| *proMdACS1-pGreenII 0800-LUC-R* | cgctctagaactagtggatccTTTGGTTAATTTTCTACTGTATGGATACA | Dual luciferase reporter assay |
| *proMdNAC72-pGreenII 0800-LUC-F* | gtcgacggtatcgataagcttCCAAACCATTCAGCTGCCG | Dual luciferase reporter assay |
| *proMdNAC72-pGreenII 0800-LUC-R* | cgctctagaactagtggatccTATTCCGGTGACAAATTTCCG | Dual luciferase reporter assay |
| *proMdMYC2-pGreenII 0800-LUC-F* | gtcgacggtatcgataagcttTTCCTTGGTTCGTCCGAGTCC | Dual luciferase reporter assay |

| *proMdMYC2-pGreenII 0800-LUC-R* | cgctctagaactagtggatccGTGATACGCAGCGTTTTGGG | Dual luciferase reporter assay |
| --- | --- | --- |

| proMdNAC18.1-ChIP-F | CAATATGACAGGAACACAGAGCATAA | ChIP-PCR |
| --- | --- | --- |
| proMdNAC18.1-ChIP-R | ACATGATGAATTGGACGAGAGATTT | ChIP-PCR |
| proMdNAC72-ChIP-F | CTAGCACCCGCACGTGGC | ChIP-PCR |
| proMdNAC72-ChIP-R | CTTGGTCAGGAGGATTCTCACC | ChIP-PCR |
| proMdMYC2-ChIP-F | TATTACCTTAAAACTTGCTGAAAAGTCC | ChIP-PCR |
| proMdMYC2-ChIP-R | TTTGTGTTCCTTGTGAACTTAGGAG | ChIP-PCR |
| proMdACS1-ChIP-F | ATTGTAACCCTAAATTCATTAATACCAAT | ChIP-PCR |
| proMdACS1-ChIP-R | AATTAATTGGTAATATAAGAATAGCCT | ChIP-PCR |
| proMdACO1-like-ChIP-F | TTATTACAAGAGTTCCAGGTAATTAGAACTC | ChIP-PCR |
| proMdACO1-like-ChIP-R | AACATAGGTTCCCGGATATCACA | ChIP-PCR |

| *F1* | CAATATGACAGGAACACAGAGCA | 58-bp indel analysis |
| --- | --- | --- |
| *R1* | GTTGTCATGCTTTTATTGCGCA | 59-bp indel analysis |
